# Trastuzumab deruxtecan in patients from China with previously treated human epidermal growth factor receptor 2–positive locally advanced/metastatic gastric or gastroesophageal junction adenocarcinoma (DESTINY-Gastric06): results from a single-arm, multicenter, phase 2 trial

**DOI:** 10.1016/j.eclinm.2025.103404

**Published:** 2025-08-11

**Authors:** Zhi Peng, Ping Chen, Jin Lu, Yiye Wan, Yulong Zheng, Feng Ye, Jianwei Yang, Ying Liu, Hongming Pan, Meili Sun, Qingxia Fan, Ying Yuan, Kai Chen, Zhuoer Sun, Han Tian, Ye Xia, Lin Shen

**Affiliations:** a State Key Laboratory of Holistic Integrative Management of Gastrointestinal Cancers, Beijing Key Laboratory of Carcinogenesis and Translational Research, Department of GI Oncology, Peking University Cancer Hospital & Institute, 52 Fucheng Road, Haidian District, Beijing, 100142, China; b General Hospital of Ningxia Medical University, 692 Shengli St, Xingqing Qu, Yinchuan Shi Ningxia Huizuzizhiqu, Yinchuan, Ningxia, 750000, China; c Sichuan Cancer Hospital, 55 Renmin South Road, Section 4, Wuhou District, Chengdu, Sichuan, 610044, China; d Jiangxi Cancer Hospital, 519 East Beijing Road, 36, Nanchang, Jiangxi, 330029, China; e The First Affiliated Hospital Zhejiang University School of Medicine, 79 Qingchun Road, Hangzhou, Zhejiang, 310003, China; f The First Affiliated Hospital of Xiamen University, 55 Zhenhai Road, Siming District, Xiamen, 361003, China; g Fujian Cancer Hospital, 420 Fuma Road, Fuzhou, Fujian Province, 350014, China; h Henan Cancer Hospital, 127 Dongming Road, Jinshui District, Zhengzhou, Henan, China; i Sir Run Run Shaw Hospital, Zhejiang University School of Medicine, East Qingchun Road, Shangcheng District, Hangzhou, Zhejiang, 310016, China; j Jinan Central Hospital, 105 Jiefang Road, Lixia District, Jinan, Shandong, 250013, China; k The First Affiliated Hospital of Zhengzhou University, 1 Jianshe East Road, Erqi District, Zhengzhou, Henan, 45005, China; l Department of Medical Oncology, The Second Affiliated Hospital, Zhejiang University School of Medicine, 88 Jie Fang Rd, Shangcheng District, Hangzhou, Zhejiang, 310009, China; m The First Affiliated Hospital of Soochow University, 296 Shizi St, Cang Lang Qu, Suzhou, Jiangsu, 215005, China; n Oncology, Oncology R&D, AstraZeneca, No. 88, North Xizang Road, Jing'An District, Shanghai, 200071, China

**Keywords:** HER2+, T-DXd, Gastric cancer, Gastroesophageal junction adenocarcinoma

## Abstract

**Background:**

Trastuzumab deruxtecan (T-DXd; 6·4 mg/kg) is approved for metastatic human epidermal growth factor receptor 2 (HER2)–positive (HER2+) gastric or gastroesophageal junction (GEJ) adenocarcinoma after a trastuzumab-based regimen. We report the final analysis of DESTINY-Gastric06, evaluating T-DXd in pretreated patients from China with advanced HER2+ gastric cancers (GC).

**Methods:**

The single-arm, multicenter, phase 2 DESTINY-Gastric06 trial (NCT04989816) enrolled patients from China with HER2+ (immunohistochemistry [IHC] 3+ or IHC 2+; locally documented) advanced gastric or GEJ adenocarcinoma with two or more prior treatments. Patients received T-DXd 6·4 mg/kg intravenous infusion every 3 weeks. The primary endpoint was confirmed objective response rate in HER2+ (IHC 3+ or IHC 2+/in situ hybridization–positive) tumors (full analysis set) by independent central review. Secondary endpoints included investigator-assessed confirmed objective response rate, progression-free survival by independent central review, overall survival, and safety.

**Findings:**

Of 126 patients screened between August 20, 2021, and December 7, 2022, 95 were enrolled (intent-to-treat; 73 patients had centrally confirmed HER2+ tumors). Median follow up was 10·2 months. Among the 73 patients, confirmed objective response rate (95% confidence interval) by independent central review was 28·8% (18·8–40·6%) and by investigator assessment was 37·0% (26·0–49·1%). Median progression-free survival by independent central review was 5·7 months. Median overall survival was 11·1 months. The most common Grade 1–2 adverse event was white blood cell count decreased (53·7%; 51/95).

**Interpretation:**

Consistent with other GC trials, T-DXd showed durable benefit, with no new safety signals, in pretreated patients from China with HER2+ advanced GC; data support T-DXd as a third- or later-line therapeutic option in this population.

**Funding:**

AstraZeneca.

**Research in context**

**Evidence before this study**

Among patients from China with gastric cancers, approximately 13% have tumors that are human epidermal growth factor receptor 2 (HER2)–positive (HER2+). We searched PubMed articles for clinical trials evaluating HER2-directed therapies in patients with previously treated HER2+ advanced gastric or gastroesophageal junction (GEJ) adenocarcinoma. The search terms included “HER2 positive” OR “HER2 expressing” AND “gastric cancer” OR “gastroesophageal junction adenocarcinoma” OR “gastroesophageal junction cancer”; the search was restricted to English-language articles published between September 1, 2014, and December 31, 2024. Results of this search were used to contextualize findings from the DESTINY-Gastric06 trial. On June 9, 2021, disitamab vedotin received conditional approval in China as a treatment for patients with HER2+ advanced gastric or gastroesophageal junction cancer who have received at least two lines of systemic therapy. However, no reports of other HER2-directed therapies approved in China were identified. Trastuzumab deruxtecan (T-DXd) 6·4 mg/kg is approved in several countries worldwide for the treatment of advanced HER2+ gastric or GEJ adenocarcinoma in patients who have received a prior trastuzumab-based regimen, and in Japan for patients with HER2+ advanced gastric cancer that has progressed after chemotherapy. Clinical trials have demonstrated antitumor activity with T-DXd in previously treated patients with HER2+ advanced gastric or GEJ adenocarcinoma. On August 13, 2024, based on findings from the primary analysis of DESTINY-Gastric06, trastuzumab deruxtecan (T-DXd) was granted conditional approval in China for adult patients with locally advanced or metastatic HER2+ gastric or GEJ adenocarcinoma who have received two or more prior treatment regimens.

**Added value of this study**

This is the final analysis of the phase 2 DESTINY-Gastric06 trial of T-DXd in patients from China with HER2+ advanced gastric or GEJ adenocarcinoma who had received two or more prior anticancer regimens, including a fluoropyrimidine agent and a platinum agent. T-DXd demonstrated clinically meaningful antitumor activity and durable treatment responses in this patient population, with a safety profile generally consistent with previous T-DXd studies.

**Implications of all the available evidence**

The findings support the use of T-DXd as a therapeutic option in the third- and later-line settings for patients from China with HER2+ advanced gastric or GEJ adenocarcinoma.

## Introduction

In China, approximately 13% of patients with gastric cancers have tumors that are human epidermal growth factor receptor 2 (HER2)–positive (HER2+).^1^ The anti-HER2 antibody trastuzumab, in combination with chemotherapy, has improved clinical outcomes in HER2+ advanced gastric cancer,^2^ and trastuzumab is the recommended first-line therapy in combination with oxaliplatin or cisplatin, and 5-fluorouracil or capecitabine for patients with HER2+ metastatic gastric cancer.^3^ Until recently, there were no HER2-directed treatment options available after the first-line setting following trastuzumab-based treatment failure, with third-line therapies including apatinib, nivolumab, and the conditionally approved disitamab vedotin.^3^

Trastuzumab deruxtecan (T-DXd) is an antibody-drug conjugate composed of a humanized immunoglobulin G1 monoclonal antibody that specifically targets HER2 (trastuzumab), a tetrapeptide-based cleavable linker, and a potent topoisomerase I inhibitor payload (an exatecan derivative, DXd).^4^, ^5^, ^6^ T-DXd 6·4 mg/kg is approved in the US and EU for patients with advanced HER2+ gastric or gastroesophageal junction (GEJ) adenocarcinoma who have received a prior trastuzumab-based regimen,^7^^,^^8^ and in the Republic of Korea and Singapore for patients with advanced HER2+ gastric or GEJ adenocarcinoma who have previously received two or more prior regimens, including a HER2-directed therapy.^9^^,^^10^ In Japan, T-DXd 6·4 mg/kg is approved for patients with HER2+ advanced gastric cancer that has progressed after chemotherapy.^11^ On August 13, 2024, based on findings from the primary analysis of DESTINY-Gastric06,^12^ T-DXd monotherapy received conditional approval in China for adult patients with locally advanced or metastatic HER2+ gastric or GEJ adenocarcinoma who have received two or more prior treatment regimens.^13^

In the DESTINY-Gastric01 trial (NCT03329690), T-DXd 6·4 mg/kg demonstrated significant clinical benefit in patients from Japan or the Republic of Korea with HER2+ locally advanced or metastatic gastric or GEJ adenocarcinoma that had progressed after at least two prior lines of therapy.^14^ The confirmed objective response rate (95% confidence interval [CI]) by independent central review was 43% (34–52%) in the T-DXd group compared with 12% (5–24%) in the chemotherapy (irinotecan or paclitaxel) group.^14^ Here, we report final analysis results from the DESTINY-Gastric06 trial, which evaluated the antitumor activity and safety of T-DXd in patients from China with HER2+ (immunohistochemistry [IHC] 3+/IHC 2+) advanced gastric or GEJ adenocarcinoma who had received two or more prior anticancer regimens.

## Methods

### Study design and participants

DESTINY-Gastric06 was a single-arm, multicenter, phase 2 trial conducted in China across 22 sites (NCT04989816).^15^ Eligible patients were aged 18 years or older at the time of screening and had pathologically documented, locally advanced, unresectable or metastatic gastric or GEJ adenocarcinoma. Patients were included if they had disease progression on or after two or more prior regimens for advanced/metastatic disease, including a fluoropyrimidine agent and a platinum agent. In addition, patients must have had an Eastern Cooperative Oncology Group performance status score of 0 or 1 and documentation of HER2 IHC 3+ or IHC 2+ status, regardless of in situ hybridization (ISH) status, during screening (determined at site based on a documented pathology report obtained from a local laboratory or previous pathology report that had been confirmed by the investigator). If HER2 status had not been documented previously and the primary tumor was accessible by endoscopy or metastatic tissue could be biopsied, a newly acquired tumor sample was provided for local assessment of HER2 status for study eligibility. However, if a newly acquired sample could not be obtained, an archival sample was acceptable, based on the most recent archival tumor tissue sample. Patients also had to be willing and able to provide an adequate newly acquired tumor sample for confirmation of HER2 status by a central laboratory specified by the sponsor, provided that the primary tumor was accessible by endoscopy or that metastatic tissue could be biopsied. However, if a newly acquired tumor sample was unavailable, the most recent archived sample was acceptable.

Patients must have had at least one lesion that could be accurately measured at baseline as at least 10 mm in the longest diameter by computed tomography or magnetic resonance imaging and was suitable for accurate repeated measurements per Response Evaluation Criteria in Solid Tumours (RECIST) 1·1. In addition, patients were required to have had adequate organ and bone marrow function within 14 days of study enrollment, left ventricular ejection fraction of at least 50% within 28 days before enrollment, minimum life expectancy of 12 weeks and adequate treatment washout period(s) before initiation of study treatment.

Female patients had to have a negative serum pregnancy test if they were of childbearing potential or be 1 year post-menopausal, surgically sterile, or using a highly effective form of birth control. Male patients intending to be sexually active with a female partner of childbearing potential must have been surgically sterile or using an acceptable method of contraception from the time of screening, throughout the trial and until four months after the last dose of study treatment.

Patients were excluded if they had any evidence of diseases, such as severe or uncontrolled systemic diseases, as judged by the investigator, pleural effusion, ascites, or a pericardial effusion that required drainage, peritoneal shunt, or cell-free and concentrated ascites reinfusion therapy. Additionally, patients were excluded if they had a history of another primary malignancy (except for malignancy treated with curative intent with no known active disease in the 3 years before the first dose of T-DXd and of low potential risk for recurrence) and unresolved toxicities from previous anticancer therapy, defined as toxicities (other than alopecia) not yet resolved to Grade ≤1 or baseline. Other exclusion criteria included presence of spinal cord compression or clinically active central nervous system metastases (defined as untreated and symptomatic, or requiring therapy corticosteroids or anticonvulsants to control associated symptoms); active primary immunodeficiency, known uncontrolled human immunodeficiency virus infection, or active hepatitis B or C infection; or an uncontrolled infection requiring intravenous antibiotics, antivirals, or antifungals. However, patients with past or resolved hepatitis B virus (HBV) infection or inactive chronic HBV infection were eligible if they met specific criteria (see protocol for further details); such patients were required to be monitored for HBV reactivation.

Patients with a history of substance abuse or other medical conditions that, in the opinion of the investigator, could interfere with study participation or assessment of study outcomes were also ineligible. Those with a history of myocardial infarction within 6 months prior to enrollment were excluded, as well as those with one or more of the following: mean resting QT interval corrected using Fridericia's formula >470 ms in females or >450 ms in males; history of QT prolongation associated with other medications that required discontinuation; congenital long QT syndrome; family history of long QT syndrome; or unexplained sudden death before 40 years of age in a first-degree relative. Patients with a history of (non-infectious) interstitial lung disease (ILD)/pneumonitis that required steroid treatment, current ILD/pneumonitis, suspected ILD/pneumonitis that could not be ruled out by imaging at screening; clinically significant lung-specific intercurrent illnesses (including but not limited to any underlying pulmonary disorder); or prior pneumonectomy were also excluded. Autoimmune, connective tissue, or inflammatory disorders were also exclusionary.

Patients with prior exposure to therapy with an inadequate washout period before enrollment, receiving any concurrent anticancer treatment, who had undergone a major surgical procedure or experienced a significant traumatic injury within four weeks prior to the first dose of study treatment, or with an anticipated need for major surgery during the trial were ineligible. Those who had received palliative radiotherapy to a limited field within 2 weeks, or to a wide field or more than 30% of the bone marrow within 4 weeks before the first dose of study treatment were also excluded. Previous treatment or randomization in a T-DXd trial regardless of treatment assignment, concurrent enrollment in another clinical trial unless observational in nature, known allergy or hypersensitivity to study treatment, and a history of severe hypersensitivity to other monoclonal antibodies were also exclusionary. Finally, involvement in the planning and/or conduct of the trial, judgment by the investigator that the patient should not participate, and being pregnant or breastfeeding were additional exclusion criteria.

### Ethics

The trial was approved by the independent ethics committee and conducted in accordance with the protocol, the Declaration of Helsinki, the Council for International Organizations of Medical Sciences International Ethical Guidelines, and the International Council for Harmonisation Good Clinical Practice Guidelines, and applicable regulatory requirements. Written informed consent was provided by all patients before enrollment. Center numbers, and ethics committee names and addresses have been provided as a Supplementary Appendix.

### Procedures

Patients received T-DXd 6·4 mg/kg by intravenous infusion once every 3 weeks until disease progression according to RECIST 1·1, unacceptable toxicity, withdrawal of consent, or any other criterion for discontinuation. All patients had a follow-up visit at 40 (+7) days after their last dose of T-DXd, followed by long-term follow-up visits every 3 months (±14 days) until death, withdrawal of consent, or end of the study.

Two dose reductions of T-DXd were permitted (5·4 mg/kg or 4·4 mg/kg). After a dose reduction due to toxicity, all subsequent cycles were administered at the lower dose level unless further dose reduction was required. Patients requiring more than two dose reductions were withdrawn from treatment. In exceptional circumstances, such as adverse event (AE) management or medical intervention, a dose could be delayed for up to 18 weeks from the last T-DXd dose.

Scanning and tumor assessments were conducted by the investigator using computed tomography (preferred) or magnetic resonance imaging of the chest, abdomen, and pelvis during screening/baseline and every 6 weeks (±7 days) during trial intervention until defined radiological progression. Patients who discontinued study treatment due to reasons other than progressive disease or death (regardless of whether they had started subsequent anticancer therapy) continued to undergo scans every 6 weeks (±7 days) for the first 48 weeks after the date of the first dose of study treatment, then every 12 weeks (±7 days) thereafter, until RECIST 1·1-defined radiological progressive disease. Patients were assessed for survival every 3 months (±14 days) following objective disease progression or discontinuation of trial intervention.

Laboratory assessments, including clinical chemistry and hematology, were conducted within 14 days prior to enrollment, on Days 1, 8, and 15 of Cycle 1, then Day 1 of every cycle, thereafter, at end of treatment and 40 (+7) days after the last dose of T-DXd. Coagulation (performed during screening) and urinalysis were evaluated as clinical indicated. Analysis of clinical chemistry, hematology, and urinalysis samples was performed at a local laboratory.

AEs were detected, documented, and reported by the investigator, and graded according to the National Cancer Institute Common Terminology Criteria for AEs and coded using the most recent version of the Medical Dictionary for Regulatory Activities. AEs of special interest included ILD/pneumonitis and left ventricular ejection fraction decrease. Potential ILD/pneumonitis cases were reviewed by the independent ILD Adjudication Committee. AEs and serious adverse events were collected throughout the treatment period, and during the safety follow-up period (40 [+7] days after discontinuation of T-DXd). Any unresolved AEs at the last visit were followed up by the investigator for as long as medically indicated, but without further recording in the electronic case report form.

Blood samples for evaluating serum pharmacokinetic concentrations of T-DXd, total anti-HER2 antibody, and topoisomerase I inhibitor payload were collected for each patient at the following timepoints: on Day 1 of Cycle 1, within 8 h prior to T-DXd infusion, within 15 min of the end of T-DXd infusion, and within 5 (±2) h of the start of T-DXd infusion; on Day 1 of Cycles 2–4, within 8 h prior to infusion and within 15 min of the end of T-DXd infusion; and on Day 1 of Cycles 6 and 8, within 8 h prior to T-DXd infusion. Additional blood samples were collected for pharmacokinetic analyses if ILD/pneumonitis was suspected. Blood samples for immunogenicity evaluations were collected on Day 1 of Cycles 1, 2, and 4, then every four cycles thereafter within 8 h prior to T-DXd infusion.

The protocol did not specify how sex and/or gender should be defined.

### Outcomes

The primary endpoint was confirmed objective response rate, defined as the proportion of patients with HER2+ (IHC 3+ or IHC 2+/ISH+) tumors who had a confirmed complete or partial response as determined by independent central review according to RECIST 1·1. Secondary endpoints included: investigator-assessed objective response rate per RECIST 1·1; duration of response (time from the first documented response until documented progression or death in the absence of disease progression using RECIST 1·1 by independent central review and investigator assessment); disease control rate (percentage of patients who had a confirmed complete response/partial response or stable disease for at least 5 weeks after the date of enrollment per RECIST 1·1 by independent central review and investigator assessment); and assessment of tumor size change (best percentage change from baseline in the sum of diameters of target lesions by independent central review and investigator assessment). Other secondary endpoints included progression-free survival (time from the date of enrollment until the date of confirmed disease progression per RECIST 1·1 as determined by independent central review and investigator assessment, or death due to any cause) and overall survival (time from enrollment until death from any cause).

Antitumor activity-associated outcomes for the HER2-expressing (IHC 3+ or IHC 2+) population were prespecified in the study protocol; however, results are not reported herein to retain focus on patients with HER2+ advanced gastric or GEJ adenocarcinoma in line with the approved indication for T-DXd in China.

Safety outcomes included occurrence of AEs, vital signs, electrocardiograms, and laboratory parameters. Coronavirus disease 2019 (COVID-19)–related, pharmacokinetic, and immunogenicity data were also collected.

### Statistics

As this was an open-label, single-arm trial, no formal statistical hypothesis was tested and descriptive statistics were used to present the data. It was estimated that a sample size of 70 patients with HER2+ disease would provide approximately 99% probability for the lower bound of the 95% CI of the objective response rate to exclude the objective response rate of 10% achieved by the standard-of-care treatment.^16^^,^^17^

In the primary analysis, the data cutoff for objective response rate by independent central review occurred approximately 6 months after the last patient initiated study treatment, as previously published.^12^ The data cutoff for the final analysis of objective response rate by independent central review occurred approximately 14 months after the last patient initiated study treatment, to allow sufficient time for patients to reach a response and for duration of response to be determined for those with a response.

The full analysis set (FAS) comprised patients with HER2 status confirmed as IHC 3+ or IHC 2+/ISH+ by central laboratory testing, and the intent-to-treat analysis set included all patients who signed the informed consent form and enrolled in the trial. Assessment of both the primary and secondary endpoints was conducted on the FAS. A subgroup analysis was conducted for the primary endpoint, comparing confirmed objective response rates in the following subgroups of the FAS: lines of prior systemic therapy, age of enrollment, sex, Eastern Cooperative Oncology Group performance status, HER2 status by central laboratory, primary tumor location, histological subtype, number of metastatic sites, and previous total gastrectomy. For the progression-free survival analysis, patients alive and without disease progression at the time of analysis were censored at the date of their most recent evaluable RECIST assessment. However, if a patient had disease progression or had died immediately after two or more consecutive missed visits, they were censored at the date of the most recent evaluable RECIST assessment prior to the missed visits. For the overall survival analysis, any patient not known to have died at the time of data analysis was censored at the last recorded date on which the patient was known to be alive. Furthermore, a sensitivity analysis was performed to assess the impact of COVID-19 on overall survival. In this analysis, any patient who died owing to COVID-19 infection, either as the primary or secondary cause, was censored at the date of death.

The safety analysis set included patients who received at least one dose of T-DXd. Additionally, the pharmacokinetic set comprised patients who received at least one dose of T-DXd and had at least one post-dose measurable serum concentration of T-DXd. For all those in the pharmacokinetic set, pharmacokinetic concentrations were listed by patient and dosing day/time and tabulated using summary statistics. Lastly, the anti-drug antibody evaluable set included patients who received at least one dose of T-DXd with a non-missing baseline anti-drug antibody result and at least one non-missing post-baseline anti-drug antibody result. The number and percentage of patients who developed detectable anti-drug antibodies to T-DXd by anti-drug antibody categories were summarized based on the T-DXd anti-drug antibody evaluable set.

Exact 95% CIs were calculated using the Clopper–Pearson method. The distribution of time-to-event endpoints for duration of response, progression-free survival, and overall survival was estimated by the Kaplan–Meier method, and the medians with two-sided 95% CI were calculated using the Brookmeyer–Crowley method with log-log transformation. Statistical Analysis System version 9·4 or higher was used for all statistical analyses.

The redacted protocol is provided separately.

This study is registered with ClinicalTrials.gov (NCT04989816) and is available at the following link: https://clinicaltrials.gov/study/NCT04989816.

### Role of the funding source

This study is sponsored by AstraZeneca. In March 2019, AstraZeneca entered into a global development and commercialization collaboration agreement with Daiichi Sankyo for trastuzumab deruxtecan (T-DXd; DS-8201). In collaboration with the authors, AstraZeneca assisted with the trial design and conception, as well as with the acquisition, analysis, and interpretation of data.

## Results

**Fig. 1:**
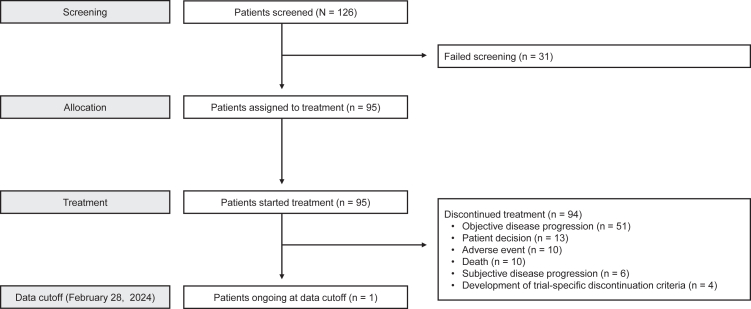
**Patient disposition**.

Between August 20, 2021, and December 7, 2022, 126 patients were screened; of these, 95 patients were enrolled and received at least one dose of T-DXd 6·4 mg/kg (Fig. 1). At the final data cutoff of February 28, 2024, a total of 94 patients had discontinued treatment; the most common reasons for treatment discontinuation were objective disease progression (54·3%), patient decision (13·8%), AEs, (10·6%), and death (10·6%).

As of the final data cutoff, 73 patients with centrally confirmed HER2+ (IHC 3+ or IHC 2+/ISH+) tumors were included in the FAS; 22 patients did not have tumors centrally confirmed as HER2+. Patients had a median (range) age of 60 (28–77) years, and 18 patients (24·7%) were female. The median (range) number of prior lines of therapy was two (two–six), and 67 patients (91·8%) had received a prior trastuzumab-based regimen. Patient demographics and disease characteristics are summarized in Table 1.

**Table 1:** Patient demographics and disease characteristics (FAS).

	N = 73
**Median age, years (range)**	60 (28–77)
**Sex, n (%)**	
Male	55 (75·3)
Female	18 (24·7)
**Eastern Cooperative Oncology Group performance status, n (%)**	
0	27 (37·0)
1	46 (63·0)
**HER2 status by central laboratory, n (%)**	
IHC 3+	53 (72·6)
IHC 2+/ISH+	20 (27·4)
**HER2 status by central laboratory (newly collected tumor biopsy),**^a^**n (%)**	
IHC 3+	4 (5·5)
IHC 2+/ISH+	0
**Primary tumor location, n (%)**	
Gastric	51 (69·9)
Gastroesophageal junction	22 (30·1)
**Sum of diameters of measurable tumors, n (%)**	
<5 cm	29 (39·7)
≥5 cm–<10 cm	30 (41·1)
≥10 cm	14 (19·2)
**Median prior lines of therapy, n (range)**	2 (2–6)
**Previous anti-HER2 therapy,**^b^**n (%)**	
Trastuzumab-containing regimen	67 (91·8)
RC48-containing regimen	11 (15·1)
KN026-containing regimen	6 (8·2)
ARX788-containing regimen	3 (4·1)
Pyrotinib maleate-containing regimen	3 (4·1)
Pyrotinib-containing regimen	2 (2·7)
**Previous immunotherapy, n (%)**	57 (60·0)
**Previous ramucirumab-containing regimen, n (%)**	2 (2·7)

FAS defined as patients with centrally confirmed HER2+ status (IHC 3+ or IHC 2+/ISH+).

FAS, full analysis set; HER2, human epidermal growth factor receptor 2; IHC, immunohistochemistry; ISH+, in situ hybridization–positive.

a Patients had a new tumor sample collected during the screening period.

b Only anti-HER2 therapies received by two or more patients are listed; therapies included anti-HER2 monoclonal and bispecific antibodies, anti-HER2 antibody-drug conjugates, and tyrosine kinase inhibitors.

**Fig. 2:**
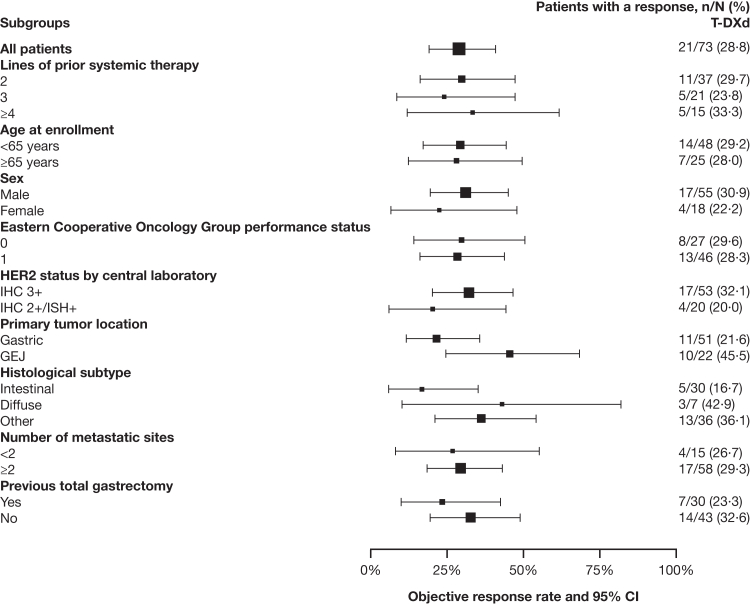
**Objective response rate analyses by subgroup (FAS)**. FAS defined as patients with centrally confirmed HER2+ status (IHC 3+ or IHC 2+/ISH+). Response assessed by independent central review as per RECIST 1·1; the size of the squares is proportional to the number of events. CI, confidence interval; FAS, full analysis set; GEJ, gastroesophageal junction; HER2, human epidermal growth factor receptor 2; HER2+, human epidermal growth factor receptor 2–positive; IHC, immunohistochemistry; ISH+, in situ hybridization–positive; RECIST, Response Evaluation Criteria in Solid Tumours; T-DXd, trastuzumab deruxtecan.

The median (quartile 1, quartile 3) duration of follow up was 10·2 (6·1, 16·9) months in the FAS. A summary of antitumor activity is shown in Table 2 and subgroup analyses in Fig. 2. In patients with centrally confirmed HER2+ tumors (N = 73), the confirmed objective response rate (95% CI) by independent central review was 28·8% (18·8–40·6%); a complete response occurred in one patient (1·4%) and a partial response occurred in 20 patients (27·4%). The objective response rate by independent central review was 32·1% (17/53) and 20·0% (4/20) in patients with HER2 IHC 3+ and IHC 2+/ISH+ tumors, respectively. Among patients who had a new tumor sample collected for HER2 status evaluation during the screening period (4/73), the confirmed objective response rate by independent central review was 50·0% (2/4). The confirmed investigator-assessed objective response rate (95% CI) was 37·0% (26·0–49·1%) in the FAS (N = 73); no patients had a complete response, and 27 patients (37·0%) had a partial response. Objective response subgroup analyses are shown in Fig. 2; a numerically higher proportion of patients with HER2 IHC 3+ tumors had an objective response by independent central review versus those with IHC 2+/ISH+ tumors (32·1% [17/53] versus 20·0% [4/20], respectively).

**Table 2:** Response to T-DXd as assessed by independent central review and investigator assessment (FAS).

	N = 73	
	Independent central review	Investigator assessment
**Objective response**		
n	21	27
Rate (95% CI), %	28·8 (18·8–40·6)	37·0 (26·0–49·1)
**Best objective response, n (%)**		
Complete response	1 (1·4)	0
Partial response	20 (27·4)	27 (37·0)
Stable disease	37 (50·7)	30 (41·1)
Progressive disease^a^	14 (19·2)	15 (20·5)
Not evaluable	1 (1·4)	1 (1·4)

FAS defined as patients with centrally confirmed HER2+ status (IHC 3+ or IHC 2+/ISH+).

CI, confidence interval; FAS, full analysis set; HER2+, human epidermal growth factor receptor 2–positive; IHC, immunohistochemistry; ISH+, in situ hybridization–positive; RECIST, Response Evaluation Criteria in Solid Tumours; T-DXd, trastuzumab deruxtecan.

a Included RECIST 1·1-defined disease progression and death ≤13 weeks without RECIST 1·1-defined disease progression.

**Fig. 3:**
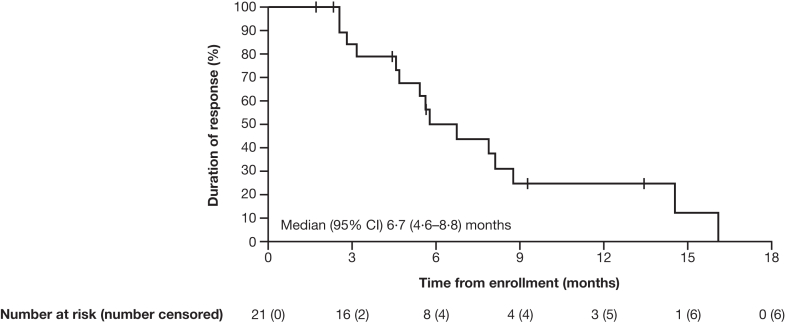
**Kaplan–Meier estimates of duration of response (FAS)**. FAS defined as patients with centrally confirmed HER2+ status (IHC 3+ or IHC 2+/ISH+). Response assessed by independent central review as per RECIST 1·1; vertical line indicates a censored observation; events that did not occur within two missed visits of the last evaluable assessment or first dose were censored; only patients who had a confirmed complete or partial response were included in the analysis. CI, confidence interval; FAS, full analysis set; HER2+, human epidermal growth factor receptor 2–positive; IHC, immunohistochemistry; ISH+, in situ hybridization–positive; RECIST, Response Evaluation Criteria in Solid Tumours.

**Fig. 4:**
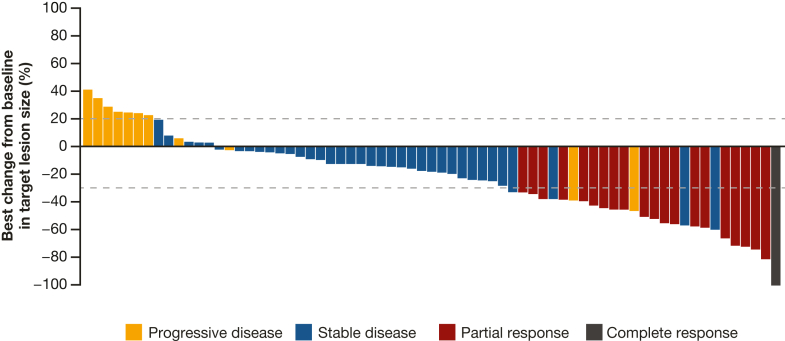
**Best percentage change from baseline in the sum of diameters of target lesions (FAS)**. FAS defined as patients with centrally confirmed HER2+ status (IHC 3+ or IHC 2+/ISH+). Assessed by independent central review as per RECIST 1·1. The best percentage change in the sum of diameters of measurable tumor was the largest decrease or smallest increase (in the absence of a decrease) from baseline. Only visits prior to subsequent antineoplastic therapy were included. Complete responses and partial responses were confirmed within 4 weeks of the visit at which the response was first observed. Best percentage change was calculated only for patients with available data at baseline and post-baseline. The dashed lines at −30% and 20% change in target lesion size indicate the thresholds for partial response and progressive disease, respectively. FAS, full analysis set; HER2+, human epidermal growth factor receptor 2–positive; IHC, immunohistochemistry; ISH+, in situ hybridization–positive; RECIST, Response Evaluation Criteria in Solid Tumours.

The median (95% CI) duration of response determined by independent central review and investigator assessment was 6·7 (4·6–8·8) months and 6·0 (4·4–8·6) months, respectively (Fig. 3). The disease control rate (95% CI) was 79·5% (68·4–88·0%) by independent central review and 78·1% (66·9–86·9%) by investigator assessment. Best percentage change in the sum of diameters of measurable tumors was evaluated for patients with both baseline and post-treatment tumor measurements; median best percentage change (range) by independent central review (n = 69) was −17·7% (−100·0 to 40·6; Fig. 4) and by investigator assessment (n = 67) was −25·0% (−100·0 to 33·7).

**Fig. 5:**
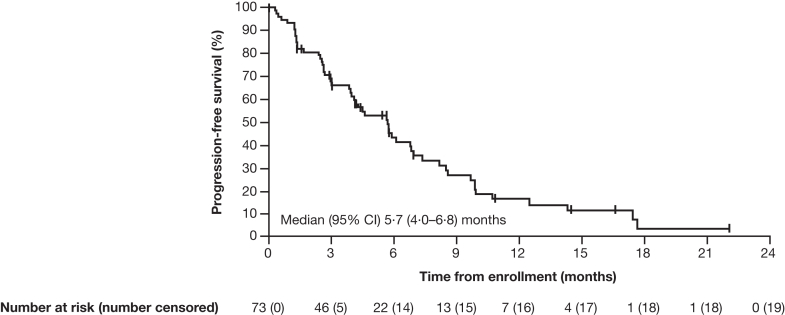
**Kaplan–Meier estimates of progression-free survival (FAS)**. FAS defined as patients with centrally confirmed HER2+ status (IHC 3+ or IHC 2+/ISH+). Assessed by independent central review as per RECIST 1·1; vertical line indicates a censored observation; events that did not occur within two missed visits of the last evaluable assessment or first dose were censored. CI, confidence interval; FAS, full analysis set; HER2+, human epidermal growth factor receptor 2–positive; IHC, immunohistochemistry; ISH+, in situ hybridization–positive; RECIST, Response Evaluation Criteria in Solid Tumours.

**Fig. 6:**
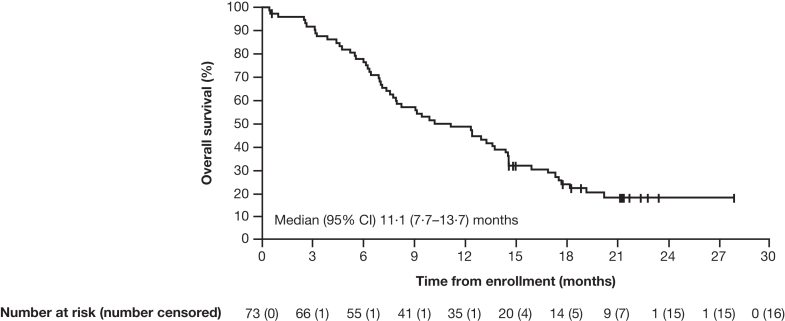
**Kaplan–Meier estimates of overall survival (FAS)**. FAS defined as patients with centrally confirmed HER2+ status (IHC 3+ or IHC 2+/ISH+). Vertical line indicates a censored observation; patients not known to have died at the time of analysis were censored at the last recorded date on which the patient was last known to be alive. CI, confidence interval; FAS, full analysis set; HER2+, human epidermal growth factor receptor 2–positive; IHC, immunohistochemistry; ISH+, in situ hybridization–positive.

Median (95% CI) progression-free survival by independent central review and investigator assessment was 5·7 (4·0–6·8) months and 5·7 (4·3–7·0) months, respectively (Fig. 5). The median (95% CI) overall survival was 11·1 (7·7–13·7) months (Fig. 6). A sensitivity analysis of COVID-19 was performed in the FAS, with 5·5% of patients censored owing to death caused by COVID-19. Results of the COVID-19 sensitivity analysis showed a median (95% CI) overall survival of 12·4 (7·9–14·5) months.

The median (range) duration of T-DXd exposure was 3·4 (0·4–22·3) months and the median (range) relative dose intensity was 97·7% (52·2–102·5%). A summary of safety data is presented in Table 3. One hundred percent of patients (N = 95) had at least one AE, and 71 patients (74·7%) had Grade 3 or worse AEs. The three most common Grade 1 or 2 AEs were white blood cell count decreased (53·7%, n = 51), anemia (50·5%, n = 48), and hypoalbuminemia (41·1%, n = 39; Table 4). The most common Grade 3 AEs (≥20%) were anemia (28·4%, n = 27) and neutrophil count decreased (22·1%, n = 21), and the only Grade 4 AE that occurred in at least 5% of patients was platelet count decreased (7·4%, n = 7). COVID-19, COVID-19 pneumonia, and death were the only Grade 5 events reported in more than one patient, each occurring in two patients each (2·1%). Drug-related AEs occurred in 94 patients (98·9%), with 64 patients (67·4%) having Grade 3 or worse events. The most common (≥20%) drug-related Grade 3 or worse AEs were neutrophil count decreased (25·3%, n = 24), anemia (24·2%, n = 23), and platelet count decreased (20·0%, n = 19).

**Table 3:** Safety summary (safety analysis set).

n (%)	N = 95
**AEs**	95 (100)
**Drug-related AEs**	94 (98·9)
**Grade ≥3 AEs**	71 (74·7)
**Drug-related Grade ≥3 AEs**	64 (67·4)
**Drug-related AEs leading to dose reductions**	25 (26·3)
**Drug-related AEs leading to treatment discontinuations**	3 (3·2)
**Drug-related SAEs leading to death**^a^	2 (2·1)
**COVID-19–associated AEs**	24 (25·3)
COVID-19	17 (17·9)
COVID-19 pneumonia	4 (4·2)
Coronavirus infection	5 (5·3)

Safety analysis set, defined as all patients who received at least one dose of T-DXd.

AE, adverse event; COVID-19, coronavirus disease 2019; SAE, serious adverse event; T-DXd, trastuzumab deruxtecan.

a Included deaths caused by pneumonia (n = 1) and pulmonary embolism (n = 1).

**Table 4:** Most common all-causality AEs in T-DXd–treated patients (safety analysis set).

n (%)	N = 95			
	Grade 1 or 2	Grade 3	Grade 4	Grade 5
White blood cell count decreased	51 (53·7)	16 (16·8)	2 (2·1)	0
Anemia	48 (50·5)	27 (28·4)	2 (2·1)	0
Hypoalbuminemia	39 (41·1)	0	0	0
Decreased appetite	38 (40·0)	0	0	0
Nausea	35 (36·8)	6 (6·3)	0	0
Neutrophil count decreased	34 (35·8)	21 (22·1)	3 (3·2)	0
Platelet count decreased	31 (32·6)	13 (13·7)	7 (7·4)	0
Aspartate aminotransferase increased	30 (31·6)	0	0	0
Vomiting	29 (30·5)	2 (2·1)	0	0
Weight decreased	27 (28·4)	0	0	0
Alanine aminotransferase increased	24 (25·3)	0	0	0
Hypocalcemia	20 (21·1)	3 (3·2)	0	0
Hyponatremia	18 (18·9)	1 (1·1)	1 (1·1)	0
Constipation	18 (18·9)	0	0	0
Hypokalemia	17 (17·9)	6 (6·3)	2 (2·1)	0
Fatigue	15 (15·8)	3 (3·2)	0	0
Asthenia	14 (14·7)	14 (14·7)	0	0
COVID-19	13 (13·7)	2 (2·1)	0	2 (2·1)
Lymphocyte count decreased	12 (12·6)	3 (3·2)	1 (1·1)	0
Diarrhea	12 (12·6)	2 (2·1)	0	0
Blood bilirubin increased	10 (10·5)	0	0	0
Gamma-glutamyltransferase increased	9 (9·5)	2 (2·1)	0	0
Blood alkaline increased	9 (9·5)	1 (1·1)	0	0
Abdominal distension	7 (7·4)	1 (1·1)	0	0
Pneumonia	6 (6·3)	1 (1·1)	0	1 (1·1)
Hepatic function abnormal	5 (5·3)	2 (2·1)	0	0
Thrombocytopenia	3 (3·2)	2 (2·1)	2 (2·1)	0
Blood creatinine increased	5 (5·3)	1 (1·1)	0	0
Abdominal pain	5 (5·3)	1 (1·1)	0	0
Cough	4 (4·2)	1 (1·1)	0	0
Back pain	4 (4·2)	1 (1·1)	0	0
Amylase increased	4 (4·2)	1 (1·1)	0	0
Electrolyte imbalance	3 (3·2)	1 (1·1)	0	0
Rash	3 (3·2)	1 (1·1)	0	0
Malaise	3 (3·2)	1 (1·1)	0	0
Gastrointestinal hemorrhage	1 (1·1)	2 (2·1)	0	1 (1·1)
COVID-19 pneumonia	1 (1·1)	1 (1·1)	0	2 (2·1)
Dysphagia	2 (2·1)	1 (1·1)	0	0
Ascites	1 (1·1)	2 (2·1)	0	0
Neutrophil count increased	1 (1·1)	1 (1·1)	0	0
Abdominal infection	1 (1·1)	1 (1·1)	0	0
Gastrointestinal infection	1 (1·1)	1 (1·1)	0	0
Leukopenia	1 (1·1)	1 (1·1)	0	0
Neutropenia	1 (1·1)	1 (1·1)	0	0
Malnutrition	1 (1·1)	1 (1·1)	0	0
Mental disorder	1 (1·1)	1 (1·1)	0	0
Hematuria	1 (1·1)	1 (1·1)	0	0
Jaundice cholestatic	0	1 (1·1)	1 (1·1)	0
Myelosuppression	0	1 (1·1)	1 (1·1)	0
Febrile neutropenia	0	0	2 (2·1)	0
Death	0	0	0	2 (2·1)
Muscular weakness	0	1 (1·1)	0	0
Renal failure	0	1 (1·1)	0	0
Pigmentation disorder	0	1 (1·1)	0	0
Hyperbilirubinemia	0	1 (1·1)	0	0
Upper gastrointestinal hemorrhage	0	1 (1·1)	0	0
Sepsis	0	1 (1·1)	0	0
Soft tissue infection	0	1 (1·1)	0	0
Cerebral infection	0	0	0	1 (1·1)
Hemorrhage intracranial	0	0	0	1 (1·1)
Vertigo	0	1 (1·1)	0	0
Deep vein thrombosis	0	1 (1·1)	0	0
Pulmonary embolism	0	0	0	1 (1·1)

Safety analysis set, defined as all patients who received at least one dose of T-DXd.

Grade 1 or 2 events in ≥10% of patients and all Grade 3, Grade 4, and Grade 5 events (with corresponding Grade 1 or 2 events) are shown. AEs by maximum Common Terminology Criteria for Adverse Events grade on preferred term level.

AE, adverse event; COVID-19, coronavirus disease 2019; T-DXd, trastuzumab deruxtecan.

Drug-related AEs leading to dose reduction were reported in 25 patients (26·3%), drug-related AEs leading to treatment discontinuation were reported in three patients (3·2%), and drug-related serious adverse events leading to death occurred in two patients (2·1%; pneumonia [n = 1] and pulmonary embolism [n = 1]).

Drug-related ILD/pneumonitis adjudicated by an independent committee occurred in three patients (3·2%; Grade 1, n = 2; Grade 2, n = 1). COVID-19–associated AEs occurred in 24 patients (25·3%), with six patients (6·3%) having Grade 3 or worse events; five patients (5·3%) discontinued treatment owing to COVID-19–associated AEs.

In the pharmacokinetic analysis set (N = 95), at the end of infusion for Cycle 1, mean (standard deviation [SD]) serum concentrations of T-DXd, total anti-HER2 antibody, and topoisomerase I inhibitor payload were 103·8 (25·8) μg/mL, 119·6 (28·5) μg/mL, and 4·0 (1·9) ng/mL, respectively (Table 5). Decreases in mean (SD) serum concentrations of total anti-HER2 antibody (109·8 [26·6] μg/mL) and topoisomerase I inhibitor payload (1·7 [0·7] ng/mL) were observed at the end of infusion for Cycle 4 (n = 57), and the mean (SD) serum concentration of T-DXd at the end of infusion for Cycle 4 was 104·3 (26·0) μg/mL.

**Table 5:** Serum concentrations of T-DXd, total anti-HER2 antibody, and topoisomerase I inhibitor payload over time.

Arithmetic mean (SD)	T-DXd, μg/mL	Total anti-HER2 antibody, μg/mL	Topoisomerase I inhibitor payload, ng/mL
End of Cycle 1 (N = 95^a^)	103·8 (25·8)	119·6 (28·5)	4·0 (1·9)
End of Cycle 2 (n = 86)	115·4 (99·4)	128·3 (107·1)	2·2 (1·1)
End of Cycle 3 (n = 66)	99·5 (31·1)	108·7 (33·9)	1·9 (0·9)
End of Cycle 4 (n = 57)	104·3 (26·0)	109·8 (26·6)	1·7 (0·7)

HER2, human epidermal growth factor receptor 2; SD, standard deviation; T-DXd, trastuzumab deruxtecan.

a Pharmacokinetic analysis set, defined as all patients who received at least one dose of T-DXd and had at least one post-dose measurable serum concentration of T-DXd.

Presence of anti-drug antibodies at baseline and/or after baseline was reported in 4·7% (4/86) of patients with an assessment available. In the anti-drug antibody evaluable set (n = 86), responses associated with treatment-induced (at and post baseline) and -boosted (at baseline and during the study period) anti-drug antibodies were observed in two patients (2·3%) and none, respectively, with an assessment available; no neutralizing anti-drug antibodies were observed.

## Discussion

Results from the DESTINY-Gastric06 trial demonstrated clinically meaningful antitumor activity for T-DXd in patients from China with HER2+ advanced gastric or GEJ adenocarcinoma whose disease had progressed after two or more prior anticancer regimens. The confirmed objective response rate by independent central review was 28·8%, and consistent T-DXd antitumor activity was observed across patient subgroups.

Survival data from this trial were generally consistent with those reported previously for T-DXd in gastric cancer.^14^ In the DESTINY-Gastric01 trial, T-DXd 6·4 mg/kg demonstrated significant clinical benefit in patients from Japan and the Republic of Korea with HER2+ locally advanced or metastatic gastric or GEJ adenocarcinoma that had progressed after at least two previous therapies, including trastuzumab. The confirmed objective response rate (95% CI) was 43% (34–52%) with T-DXd compared with 12% (5–24%) with physician's choice of irinotecan or paclitaxel.^14^ The confirmed objective response rate in DESTINY-Gastric06 was numerically lower than that observed in DESTINY-Gastric01, which may be attributable to multiple differences across the trials, including patient characteristics, prior use of HER2-directed therapies, disease burden, tumor size, and number of prior lines of therapy. In addition, the T-DXd treatment and follow-up periods for DESTINY-Gastric06 occurred during the peak of the COVID-19 pandemic, leading to delays in treatment and impacting attendance at follow-up clinic visits. Consequently, antitumor activity data reported in this trial may also be affected by the number of SARS-CoV-2 infections.

In the DESTINY-Gastric06 trial, of the four patients who had a new tumor sample collected for HER2 status evaluation during the study screening period, two (50·0%) had a confirmed response to T-DXd. A recent exploratory analysis of the DESTINY-Gastric01 trial showed an objective response rate of 48·8% (40/82) for patients treated with T-DXd who had a tumor sample collected any time before their first treatment with trastuzumab and 56·8% (21/37) for those who had a tumor sample collected during/after treatment with trastuzumab, demonstrating T-DXd activity irrespective of the timing of sample collection.^18^ Some studies have reported a decrease in HER2 expression following HER2-directed therapy, which may affect outcomes for patients receiving subsequent HER2-targeted treatment in later-line settings.^19^^,^^20^ However, owing to the small number of patients in DESTINY-Gastric06 who had a new tumor sample collected to determine HER2 status during the study screening period, further investigation is needed into the potential effect of prior HER2-directed therapies and HER2 expression.

Of note, findings from this trial showed a median (range) relative dose intensity of 97·7% (52·2–102·5%). Furthermore, the median (range) duration of T-DXd exposure was 3·4 (0·4–22·3) months; in DESTINY-Gastric01, median (range) duration of treatment was 4·6 (0·7–22·3) months in the T-DXd group and 2·8 (0·5–13·1) months in the physician's choice group, demonstrating a similar median duration of T-DXd treatment between the two trials.^14^ Taken together, these results suggest an appropriate level of T-DXd administration in DESTINY-Gastric06; thus, the numerically lower confirmed objective response rate in DESTINY-Gastric06 versus DESTINY-Gastric01 is unlikely owing to reduced T-DXd exposure.

Drug-related ILD/pneumonitis events remain important to monitor with T-DXd; in DESTINY-Gastric06, events occurred in three patients (3%), with two Grade 1 events and one Grade 2 event, and there were no ILD-related safety concerns in the trial. Indeed, the overall incidence of adjudicated ILD/pneumonitis was numerically lower than that reported in previous trials of T-DXd in gastric cancer; in DESTINY-Gastric01, 12 patients (10%) in the T-DXd group had adjudicated drug-related ILD/pneumonitis and in DESTINY-Gastric02, adjudicated drug-related ILD/pneumonitis events occurred in eight patients (10%).^14^^,^^21^ The discrepancies in incidence may be explained by differences in trial design, patient characteristics, and duration of treatment exposure.

The overall T-DXd safety profile in DESTINY-Gastric06 was consistent with the established profile, and no new signals were identified.^14^^,^^21^^,^^22^ Furthermore, although COVID-19–associated events occurred in 24 patients (25·3%) in DESTINY-Gastric06 and infection with SARS-CoV-2 may contribute to an increased risk of AEs,^23^ the overall safety profile was comparable with that seen in other studies.^14^^,^^21^ However, because non–drug-related AEs were not required to be collected after the safety follow-up period, the number of patients infected with SARS-CoV-2 may have been underestimated.

Pharmacokinetic analyses were as expected and generally aligned with data reported previously in a study of patients with pretreated metastatic HER2-expressing breast cancer, with T-DXd stable in circulation.^6^ Concentrations of T-DXd were similar to total anti-HER2 antibody, and the concentrations of topoisomerase I inhibitor payload were lower than those of T-DXd and total anti-HER2 antibody. Incidence of anti-drug antibodies was low, and no patients had neutralizing antibodies.

Limitations of the DESTINY-Gastric06 trial included the relatively small sample size and the absence of a comparator or placebo group. Nevertheless, the lower bound of the 95% CI (18·8%) for confirmed objective response rate observed in this trial was numerically higher than the objective response rate of 10% reported for the standard of care, which was calculated based on historical studies in Asia reporting objective response rates between 2·8% and 11·2% for apatinib- and nivolumab-based standard-of-care regimens.^3^^,^^16^^,^^17^ In addition, the DESTINY-Gastric01 trial evaluated T-DXd as a third- or later-line therapy in HER2+ gastric cancers in patients from Japan or the Republic of Korea compared with physician's choice of chemotherapy, demonstrating clinically meaningful and durable responses with T-DXd versus standard of care for this treatment setting.^14^ There were also challenges associated with the COVID-19 pandemic owing to disease control restrictions in place that affected treatment initiation and follow up in DESTINY-Gastric06; however, the overall safety profile was consistent with that previously observed for T-DXd treatment.^14^^,^^21^ Finally, the median follow-up duration in DESTINY-Gastric06 was 10·2 months, which was shorter than the median overall survival of 11·1 months. However, per the protocol and based on DESTINY-Gastric01, the data cutoff for the full final analysis of objective response rate by independent central review occurred approximately 14 months after the last patient initiated study treatment, allowing sufficient time for patients to reach a response and for duration of response to be determined for those with a response.

Recent results from the phase 3 DESTINY-Gastric04 trial (NCT04704934) demonstrated a statistically significant and clinically meaningful improvement in overall survival with T-DXd compared with ramucirumab and paclitaxel in patients with HER2+ unresectable and/or metastatic gastric or GEJ adenocarcinoma whose disease has progressed on or after a trastuzumab-containing regimen.^24^

In conclusion, consistent with findings from other trials of T-DXd in gastric cancers,^14^^,^^21^^,^^24^ T-DXd showed durable clinical benefit, with no new safety signals identified, in patients from China with previously treated HER2+ advanced gastric cancer or GEJ adenocarcinoma. These final analysis data from DESTINY-Gastric06 support T-DXd as a third- or later-line therapeutic option in this patient population.

## Contributors

All authors were involved in data collection, interpretation of data, and had full access to all data from the study. All authors were involved in drafting and reviewing the manuscript, approved the final version, and accept responsibility to submit for publication. ZP, YX, ZS, and HT have directly accessed and verified the underlying data reported in the manuscript.

## Data sharing statement

A description of the clinical study and summary of the main study results may be obtained from http://astrazenecaclinicaltrials.com, http://www.clinicaltrials.gov, and https://www.clinicaltrialsregister.eu/. The clinical study and/or summary of main study results may also be available from www.chinadrugtrials.org.cn according to the regulations of China, the country in which the study was conducted.

Data underlying the findings described in this manuscript may be obtained in accordance with AstraZeneca's data sharing policy described at https://www.astrazenecaclinicaltrials.com/our-transparency-commitments/.

Data for studies directly listed on Vivli can be requested through Vivli at www.vivli.org. Data for studies not listed on Vivli could be requested through Vivli at https://vivli.org/members/enquiries-about-studies-not-listed-on-the-vivli-platform/. AstraZeneca Vivli member page is also available outlining further details: https://vivli.org/ourmember/astrazeneca/.

## Declaration of interests

ZP reports consulting for AstraZeneca and BeiGene; PC, JL, YW, YZ, FY, JY, YL, HP, MS, QF, YY, and KC all report no conflict of interests; ZS, HT, and YX all report support for the present manuscript from AstraZeneca and stock or stock options for AstraZeneca; and LS reports grants or contracts from BeiGene and participation on a data safety monitoring board or advisory board with AstraZeneca, Boehringer Ingelheim, MSD, Servier, AstraZeneca, and Transcenta Holding Ltd.

## Appendix A Supplementary data

Redacted Protocol
