# Supplementary material for: Trastuzumab deruxtecan in patients from China with previously treated human epidermal growth factor receptor 2–positive locally advanced/metastatic gastric or gastroesophageal junction adenocarcinoma (DESTINY-Gastric06): results from a single-arm, multicenter, phase 2 trial
Source: eClinicalMedicine. 2025 Aug 11;87:103404. doi: 10.1016/j.eclinm.2025.103404 (PMC12359162; doi:10.1016/j.eclinm.2025.103404)
Supplement: Redacted Protocol [file mmc1.pdf]

Clinical Study Protocol

Study Intervention	Trastuzumab deruxtecan (T-DXd, AZD4552, DS-8201a)
Study Code	D9676C00002
Version	2.0
Date	20DEC2021

A Single-arm Study of Trastuzumab Deruxtecan (T-DXd) Monotherapy for Patients with HER2-expressing Locally Advanced or Metastatic Gastric or Gastroesophageal Junction (GEJ) Adenocarcinoma Who Have Received 2 or More Prior Regimens (DESTINY-Gastric06)

Sponsor Name:

AstraZeneca AB, 151 85 Södertälje, Sweden

Regulatory Agency Identifier Number(s)

This CSP has been subject to a peer review according to AstraZeneca Standard procedures. The CSP is publicly registered and the results are disclosed and/or published according to the AstraZeneca Global Policy on Bioethics and in compliance with prevailing laws and regulations.

Protocol Number: D9676C00002

Amendment Number: 1

Study Intervention: Trastuzumab deruxtecan (T-DXd; DS-8201a)

The non-proprietary name of T-DXd is trastuzumab deruxtecan except in the United States where it is fam-trastuzumab deruxtecan-nxki.

Study Phase: Phase II

Short Title:

Study of T-DXd Monotherapy in Patients with HER2-expressing Locally Advanced or Metastatic Gastric or GEJ Adenocarcinoma who have Received 2 or More Prior Regimens

Acronym: DESTINY-Gastric06

Study Physician Name and Contact Information will be provided separately

National Co-ordinating Investigator:

[redacted]

VERSION HISTORY

Version	Date
Amendment 1	20-Dec-2021
Original Protocol	17-Dec-2020

Amendment 1

Overall Rationale for the amendment

Section number and name	Description of change	Brief rationale	Substantial/Non-substantial
Study title	Change ‘Her2-positive’ to ‘Her2-expressing’.	Population expansion.	S
Short title			

1.1 Synopsis-Rationale	Throughout the CSP, the eligible patients is changed from HER2 IHC 3+ or IHC 2+/ISH+ to HER2 IHC 3+ or IHC 2+.<br>Add the background information relating to HER2 IHC2+ gastric cancer.<br>Add the updating therapy of anti-HER2 treatment in 3L+ GC.<br>Add the efficacy results of T-DXd in HER2 IHC2+/ISH- and newly approved RC48 in HER2 IHC2+ population.	Expand population to HER2 IHC 3+ or IHC 2+ (regardless of ISH status) reflects the expected change of anti-HER2 treatment, and the potential of T-DXd efficacy may extend to IHC2+/ISH- population.	S
1.2 Schema			
1.3.1 Screening and Enrolment Period			
2.1 Study Rationale			
2.2.2 Current Treatment Options for HER2-positive Gastric Cancer			
Table 4 Objectives and Endpoints			
4.1 Overall Design			
4.2.1 Rationale for Study Design			
4.2.2 Rationale for T-DXd in HER2 expressing Gastric and GEJ Adenocarcinoma			
5 STUDY POPULATION			
5.1 Inclusion Criteria			
9.2 [redacted]	[redacted]	Expand population to HER2 IHC 3+ or IHC 2+ (regardless of ISH status) reflects the expected change of anti-HER2 treatment, and the potential of T-DXd efficacy may extend to IHC2+/ISH- population.	S
9.3 Population for Analyses	Defined corresponding analysis populations for the HER2-expressing population		
9.4.2.4 Secondary Endpoints in HER2-expressing Population	Added planned analyses for endpoints in the HER2-expressing population, which will follow the same approach for endpoints in theHER2-positive population		

9.1 Statistical Hypotheses<br>9.4.5 Methods for Multiplicity Control	Added gated approach for the HER2-expressing population analyses, through which type 1 error will be controlled		
1.1 Synopsis<br>4.2.1 Rationale for Study Design<br>4.2.2 Rationale for T-Dxd in Her2-expressing Gastric and GEJ Adenocarcinoma<br>4.3 Justification for dose of TDxd<br>4.4 End of Study Definition<br>9.2 Sample Size Determination<br>9.4.1 General Considerations	Update study name DS8201-A-J202 to DESTINY-Gastric01.	Correction	NS
1.1 Synopsis-Objectives and Endpoints<br>3 OBJECTIVES AND ENDPOINTS<br>9.4.2.3.5 Disease Control Rate	Correct the definition of DCR	Correction	NS
1.1 Synopsis-Objectives and Endpoints<br>3 Objectives and Endpoints	Evaluate the efficacy and safety of the HER2-expessing (IHC 3+ or IHC 2+) patients as a secondary endpoint in addition to the primary population of the HER2-positive patients being studied.	HER2 IHC 3+ or IHC 2+ (regardless of ISH status) population is added in the study.	S
1.1 Synopsis-Objectives and Endpoints	Update definition of PFS	To be consistent with analysis population and ITT principle	NS

3 Objectives and Endpoints			
9.4.2.3.2 Progression-free Survival			
1.1 Synopsis-Objectives and Endpoints	Update definition of OS	To be consistent with analysis population and ITT principle	NS
3 Objectives and Endpoints			
9.4.2.3.3 Overall Survival			
2.2.3 Trastuzumab Deruxtecan	Trastuzumab Deruxtecan approval status updated.	Updated to reflect new indication approval of Trastuzumab Deruxtecan.	NS
4.1.1 Study Duration	Study duration is updated.	Enrolment period is extended due to this CSP amendment.	NS
Table 1 Schedule of Activities: Screening and Enrolment	Change the timing of collecting the Tissue sample for central laboratory confirmation of HER2 status	To be more reasonable for actual site practice.	NS
4.1 Overall Design			
8.6.1 Collection of Mandatory Samples for Central Biomarker Analysis			
Table 1 Schedule of Activities: Screening and Enrolment	To widen the inclusion of resolved or inactive HBV subjects, updated the exclusion criteria, added lab tests monitoring requirement and clarifications for eligible HBV infection subjects, add requirement for permitted/rescue therapies and reporting HBV reactivation case within 24 hours. TMG is updated to add management of HBV reactivation.	Patients with chronic Hepatitis B (CHB) infection are typically excluded from oncology trials, this study refined the existing exclusion criteria and allow resolved or inactive HBV subjects participate the study and to develop recommendations for screening, monitoring, and management.	S
Table 2 Schedule of Activities: Study Intervention Period			
2.3.1.4 Anti-cancer treatments and immunosuppressants in eligible patients with Hepatitis B infection.			
5.2 Exclusion Criteria for past or resolved HBV or inactive chronic HBV infection.			

6.5.2 Other Protocol Restrictions or Supportive Treatments			
7.1.1 Follow-up of Participants Post Discontinuation of Study Intervention			
8.2.5.5 Safety assessment for Eligible HBV infection only			
8.3.1 Time Period and Frequency for Collecting AE and SAE Information			
Appendix K Toxicity Management Guideline			
Table 2<br>Schedule of Activities: Study Intervention Period	Wording updated following PSSR V6	Updated to reflect latest safety information.	S
2.3.1.1 Potential Risks of T-DXd			
Table 6<br>Adequate Treatment Washout Period			
5.1 Inclusion Criteria			
5.2 Exclusion Criteria for unresolved toxicities and for spinal cord compression or active central nervous system metastases.			
8.2.3 Electrocardiograms			
8.3.11<br>Adverse Events of Special Interest			
Appendix D2<br>Definitions for potential Hy's law and Hy's law case			
Appendix D3<br>Identification of Potential Hy's Law Cases			

Appendix F Contraception requirement<br>Table 18 For Highly Effective Methods of Contraception<br>Appendix J Guidance for Management of Participants with Drug induced ILD/Pneumonitis<br>Appendix K Toxicity Management Guideline			
6.2.1 T-DXd Preparation, Administration and Storage	Clarification on dose recalculation.	Clarification.	NS
6.6 Dose Modification	Removal of 49 day discontinuation criteria, updated as [redacted] to be the time frame for discontinuation, the criteria for drug resuming is added.	To allow longer time frame for drug discontinuation.	S
1.1 Synopsis-Follow-up of participants post discontinuation of study intervention<br>8.1.1 Imaging Tumour Assessments	Continue treatment is based on INV's tumour assessment, not 'by ICR assessment'.	Typo correction.	NS
8.6.1 Collection of Mandatory Samples for Central Biomarker Analysis	To revise the wording more properly to reflect the actual requirement of tumour submission to meet regulatory requirement.<br>To add CDx filing related wording to support potential CDx requirement.	NMPA CDx policies evolve rapidly, CDx filing might be required in the future.	NS
8.6.1 Collection of Mandatory Samples for Central Biomarker Analysis	Add computer-aided analysis to explore clinical response-associated biomarkers.	Develop digital algorithm to better select patient in future.	NS
Table 2 Schedule of Activities: Study Intervention Period	Delete Tumour imaging (RECIST 1.1) in C1D1	Clarify that tumour imaging (RECIST 1.1) no need to be done on Cycle 1 day 1.	NS

Table 9<br>Laboratory Safety Variables	Remove the duplicate testing items, add percentage of Leukocyte differential count and Urea	Typo correction of Laboratory Safety Variables.	NS
Table 2<br>Schedule of Activities: Study Intervention Period	Remove requirement of Pharmacokinetic Assessment(s) if Chloroquine or Hydroxychloroquine is Administered	It is not feasible to collect PK sample if patient is diagnosed as COVID19 in China.	NS
Appendix H<br>Instructions Related to Severe Acute Respiratory Syndrome Coronavirus 2 (COVID 19)			
Table 3<br>Schedule of Activities: Post-study Intervention Follow-up and Survival	Remove requirement of ADA sample collection at Long- term Follow-Up period	Immunogenicity of T-DXd is considered to be low risk.	NS
8.5.2 Immunogenicity Assessments			
Appendix D 8<br>Laboratory Tests	Clarify that local laboratory will be used for safety tests and the Hy's Law Laboratory Kit is not applicable.	Clarification added to reflect China practice.	NS
Table 10 Spirometry Components	Update FEV to FEV1.	Typo	NS
Appendix L<br>Abbreviations	New abbreviations was added, as required.	Minor updates to reflect changes to the protocol.	NS
11 Reference	New references were added, as required.	Add references to reflect changes to the protocol.	NS

TABLE OF CONTENTS

TITLE PAGE 1
VERSION HISTORY 2
TABLE OF CONTENTS 9
LIST OF FIGURES 12
LIST OF TABLES 12
1 PROTOCOL SUMMARY 14
1.1 Synopsis 14
1.2 Schema 21
1.3 Schedule of Activities 21
1.3.1 Screening and Enrolment Period 22
1.3.2 Study Intervention Period 23
1.3.3 Post-study Intervention Follow-up Period 23
2 INTRODUCTION 33
2.1 Study Rationale 33
2.2 Background 35
2.2.1 HER2-positive Gastric Cancer 35
2.2.2 Current Treatment Options for HER2-positive Gastric Cancer 36
2.2.3 Trastuzumab Deruxtecan 37
2.3 Benefit/Risk Assessment 38
2.3.1 Risk Assessment 38
2.3.1.1 Potential Risks of T-DXd 38
2.3.1.2 Potential Risks of HER2-targeted Agents 39
2.3.1.3 Potential Risks of Topoisomerase I Inhibitors 40
2.3.1.4 Anti-cancer treatments and immunosuppressants in eligible patients with Hepatitis B infection. 41
2.3.2 Benefit Assessment 41
2.3.3 Overall Benefit: Risk Conclusion 42
3 OBJECTIVES AND ENDPOINTS 42
4 STUDY DESIGN 46
4.1 Overall Design 46
4.1.1 Study Duration 47
4.1.2 Duration of Study Participation 47
4.1.3 Study Conduct Mitigation During Study Disruptions Due to Cases of Civil Crisis, Natural Disaster, or Public Health Crisis 47
4.2 Scientific Rationale for Study Design 48
4.2.1 Rationale for Study Design 48
4.2.2 Rationale for T-DXd in HER2-expressing Gastric and GEJ Adenocarcinoma 49
4.2.3 Rationale for Study Endpoints 50
4.3 Justification for Dose of T-DXd 50

4.4 End of Study Definition 52
5 STUDY POPULATION 52
5.1 Inclusion Criteria 53
5.2 Exclusion Criteria 56
5.3 Lifestyle Considerations 60
5.4 Screen Failures 61
6 STUDY INTERVENTION 61
6.1 Study Interventions Administered 61
6.1.1 Investigational Products 61
6.2 Preparation/Handling/Storage/Accountability of Interventions 62
6.2.1 T-DXd Preparation, Administration and Storage 63
6.3 Measures to Minimise Bias 64
6.4 Study Intervention Compliance 66
6.5 Concomitant Therapy 66
6.5.1 Prohibited Concomitant Medications 67
6.5.2 Other Protocol Restrictions or Supportive Treatments 68
6.6 Dose Modification 69
6.7 Intervention after the End of the Study 72
7 DISCONTINUATION OF STUDY INTERVENTION AND PARTICIPANT DISCONTINUATION/WITHDRAWAL 73
7.1 Discontinuation of Study Intervention 73
7.1.1 Follow-up of Participants Post Discontinuation of Study Intervention 74
7.1.2 Follow-up for Survival 74
7.2 Participant Withdrawal from the Study 74
7.3 Lost to Follow-up 75
8 STUDY ASSESSMENTS AND PROCEDURES 76
8.1 Efficacy Assessments 77
8.1.1 Imaging Tumour Assessments 77
8.1.2 Central Reading of Scans 78
8.1.3 Overall Survival 78
8.1.4 Clinical Outcome Assessments 78
8.2 Safety Assessments 78
8.2.1 Physical Examinations 79
8.2.2 Vital Signs 79
8.2.3 Electrocardiograms 80
8.2.4 Clinical Safety Laboratory Assessments 81
8.2.5 Other Safety Assessments 83
8.2.5.1 Echocardiogram/Multigated Acquisition Scan 83
8.2.5.2 Pulmonary Assessments 83
8.2.5.3 ECOG Performance Status 85
8.2.5.4 Ophthalmological Assessments 86

8.2.5.5 Safety assessment for Eligible HBV infection only ... 86
8.3 Adverse Events and Serious Adverse Events ... 86
8.3.1 Time Period and Frequency for Collecting AE and SAE Information ... 87
8.3.2 Follow-up of AEs and SAEs ... 88
8.3.3 Causality Collection ... 89
8.3.4 Adverse Events Based on Signs and Symptoms ... 89
8.3.5 Adverse Events Based on Examinations and Tests ... 89
8.3.6 Hy's Law ... 90
8.3.7 Disease Progression ... 90
8.3.8 Disease Under Study ... 90
8.3.9 New Cancers ... 90
8.3.10 Deaths ... 91
8.3.11 Adverse Events of Special Interest ... 91
8.3.12 Safety Data to be Collected Following Final Study Data Cutoff ... 92
8.3.13 Reporting of Serious Adverse Events ... 93
8.3.14 Pregnancy ... 93
8.3.14.1 Maternal Exposure ... 93
8.3.14.2 Paternal Exposure ... 94
8.3.15 Medication Error ... 94
8.4 Overdose ... 95
8.5 Human Biological Samples ... 95
8.5.1 Pharmacokinetics ... 95
8.5.1.1 Determination of Drug Concentration ... 96
8.5.2 Immunogenicity Assessments ... 96
8.6 Human Biological Sample Biomarkers ... 96
8.6.1 Collection of Mandatory Samples for Central Biomarker Analysis ... 96
9 STATISTICAL CONSIDERATIONS ... 97
9.1 Statistical Hypotheses ... 98
9.2 Sample Size Determination ... 98
9.3 Populations for Analyses ... 99
9.4 Statistical Analyses ... 100
9.4.1 General Considerations ... 100
9.4.2 Efficacy ... 102
9.4.2.1 Calculation or Derivation of Tumour Response Variable ... 102
9.4.2.2 Primary Endpoint: Confirmed ORR by ICR per RECIST 1.1 ... 102
9.4.2.3 Secondary Endpoints in HER2 Positive Population ... 103
9.4.2.4 Secondary Endpoints in HER2-expressing Population ... 107
9.4.3 Safety ... 107
9.4.4 Other Analyses ... 109
9.4.4.1 Pharmacokinetics ... 109
9.4.4.2 Immunogenicity Data ... 109
9.4.4.3 COVID-19 ... 109
9.4.5 Methods for Multiplicity Control ... 110
9.5 Interim Analyses ... 110

9.6 Data Monitoring Committees ... 110
9.6.1 ILD Adjudication Committee ... 110
10 SUPPORTING DOCUMENTATION AND OPERATIONAL CONSIDERATIONS ... 110
11 REFERENCES ... 165

LIST OF FIGURES

Figure 1 Study Design ... 21

LIST OF TABLES

Table 1 Schedule of Activities: Screening and Enrolment ... 24
Table 2 Schedule of Activities: Study Intervention Period ... 27
Table 3 Schedule of Activities: Post-study Intervention Follow-up and Survival ... 31
Table 4 Objectives and Endpoints ... 42
Table 5 Parameters for Adequate Organ and Bone Marrow Function ... 54
Table 6 Adequate Treatment Washout Period ... 55
Table 7 Study Intervention: T-DXd ... 62
Table 8 Dose Reduction Levels of T-DXd ... 69
Table 9 Laboratory Safety Variables ... 82
Table 10 Spirometry Components ... 84
Table 11 Populations for Analysis ... 99
Table 12 Summary of Outcome Variables and Analysis Populations ... 101
Table 13 Hy's Law Laboratory Kit for Central Laboratories ... 128
Table 14 Summary of Imaging Modalities for Tumour Assessment ... 130
Table 15 RECIST 1.1 Evaluation of Target Lesions ... 137
Table 16 RECIST 1.1 Evaluation of Non-target Lesions ... 138
Table 17 RECIST 1.1 Overall Visit Response ... 139
Table 18 Highly Effective Methods of Contraception (< 1% Failure Rate) ... 143
Table 19 Restricted Medications/Therapies ... 144
Table 20 Prohibited Medications/Therapies ... 145
Table 21 Supportive Medications/Therapies ... 146
Table 22 COVID-19 Dose Modification Criteria ... 149
Table 23 Toxicity Management Guidelines for T-DXd ... 153

LIST OF APPENDICES

Appendix A Regulatory, Ethical, and Study Oversight Considerations 111
Appendix B Adverse Events: Definitions and Procedures for Recording, Evaluating, Follow-up, and Reporting 116
Appendix C Handling of Human Biological Samples 121
Appendix D Actions Required in Cases of Increases in Liver Biochemistry and Evaluation of Hy's Law 124
Appendix E Guidelines for Evaluation of Objective Tumour Response Using RECIST 1.1 Criteria (Response Evaluation Criteria in Solid Tumours).. 130
Appendix F Contraception Requirements 141
Appendix G Concomitant Medications 144
Appendix H Instructions Related to Severe Acute Respiratory Syndrome Coronavirus 2 (COVID-19) 148
Appendix I Changes Related to Mitigation of Study Disruptions Due to Cases of Civil Crisis, Natural Disaster, or Public Health Crisis 151
Appendix J Guidance for Management of Participants with Drug-induced ILD/Pneumonitis 152
Appendix K Toxicity Management Guidelines 153
Appendix L Abbreviations 161

1 PROTOCOL SUMMARY

1.1 Synopsis

Protocol Title: A Single-arm Study of Trastuzumab Deruxtecan (T-DXd) Monotherapy for Patients with HER2-expressing Locally Advanced or Metastatic Gastric or Gastroesophageal Junction (GEJ) Adenocarcinoma Who Have Received 2 or More Prior Regimens (DESTINY-Gastric06)

Short Title: Study of T-DXd Monotherapy in Patients with HER2-expressing Locally Advanced or Metastatic Gastric or GEJ Adenocarcinoma who have Received 2 or More Prior Regimens

Rationale:

Human epidermal growth factor receptor 2 (HER2) is an important target protein for gastric cancer (GC) therapy. HER2-expression initiates a strong pro-tumorigenic signaling cascade and HER2-positive status is associated with decreased survival and several clinicopathological features of tumor progression. HER2 testing is recommended for all patients with gastric adenocarcinoma at the time of diagnosis if metastatic disease is documented or suspected. Hofmann et al refined HER2-positive as cases that have an immunohistochemistry (IHC) score of 3+ or an IHC score 2+ and in situ hybridization (ISH) positive. In China, HER2-positivity is reported in 12% to 13% of patients with GC. A score of IHC 0 or 1+ is considered to be HER2-negative. A score of IHC 2+ is considered equivocal and should be additionally examined by ISH methods. However, the testing rate of ISH in the IHC 2+ population in China is very low (<10%). Regardless of ISH results, there are 22% to 24% of GC patients in China have HER2 expressing (defined as IHC 3+ or IHC 2+ in this study) status.

Metastatic GC has a poor prognosis. In China, approved treatment options after failure of trastuzumab are limited to single-agent chemotherapy, followed by third-line therapy (regardless of HER2 status) with apatinib, nivolumab or single agent chemotherapy, or with a recently (June 2021) NMPA conditionally approved (not recommended in guidelines) anti-HER2 antibody drug conjugate (ADC) RC-48.

Given that most patients with HER2-positive advanced GC eventually acquire resistance or show intolerance to approved first- or second-line therapies and no HER2-targeting treatment recommended beyond the first-line setting in China, HER2-positive advanced GC is a serious, life-threatening disease with a high unmet medical need. Trastuzumab deruxtecan (T-DXd) is a HER2-targeting ADC being developed as a treatment for several HER2-expressing tumours, including GC. The current study aims to evaluate the efficacy and safety of T-DXd monotherapy in patients in China with HER2-positive locally advanced or metastatic gastric or gastroesophageal junction (GEJ) adenocarcinoma who have received 2 or more prior regimens in order to assess efficacy and safety in a Chinese population. The study population closely follows that of a recent study in Japan and Korea (Study DESTINY-Gastric01) in

which the efficacy and safety of T-DXd (6.4 mg/kg) were evaluated in participants with HER2-positive advanced gastric or GEJ adenocarcinoma who had progressed on at least 2 prior regimens including a fluoropyrimidine agent, a platinum agent, and trastuzumab compared with the physician's choice of treatment (irinotecan or paclitaxel monotherapy). At the data cutoff (DCO) for the primary analysis, the confirmed objective response rate (ORR) per Response Evaluation Criteria in Solid Tumours, Version 1.1 (RECIST 1.1) by independent central review (ICR) was 40.5% (95% confidence interval [CI]: 31.8, 49.6) in the T-DXd group with 51/126 participants having a best overall response of complete response (CR) (10 [7.9%] participants) or partial response (PR) (41 [32.0%] participants) and significantly higher than that for the comparator group where the ORR was 11.3% (95% CI: 4.7, 21.9) with 7/62 participants having a best overall response of PR (7 [11.3%] participants) ($p < 0.0001$). The unconfirmed ORR per RECIST 1.1 by ICR was 51.3% (95% CI: 41.9, 60.5) in the T-DXd group with 61/119 participants having a best overall response of CR (11 [9.2%] participants) or PR (50 [42.0%] participants), significantly higher than for the comparator group where the ORR was 14.3% (95% CI: 6.4, 26.2) with 8/56 participants having a best overall response of PR (8 [14.3%] participants) ($p < 0.0001$).

Primary analysis of another Phase 2 single arm trial (Study DESTINY-Gastric02) which investigated T-DXd (6.4 mg/kg) in western patients with HER2 positive unresectable or metastatic gastric or GEJ cancer who progressed on or after a Trastuzumab containing regimen showed comparable efficacy in US and European populations in addition to Korean and Japanese populations. The confirmed ORR was 38% (95% CI, 27.3-49.6) with 30/79 participants having a best overall response of CR (3 [3.8%] participants) or PR (27 [34.2%] participants).

Given that no anti-HER2 therapy is recommended for HER2-positive GC patients in China who have failed a trastuzumab-containing regimen and that the ORR for the current standard of care (SoC) in the third-line setting in China is approximately [redacted] (consistent with the findings of Study DESTINY-Gastric01), there is a clear rationale for evaluating the potential of T-DXd in the treatment of HER2-positive GC patients in China who have received 2 or more prior regimens, including a fluoropyrimidine agent and a platinum agent.

Results from Study [redacted] also showed T-DXd has benefit in [redacted] population, confirmed ORR by ICR (FAS) in [redacted] [95% CI: [redacted]])was comparable to that in [redacted] population ([redacted] [95% CI: [redacted]]). These data suggest that the antitumor activity of T-DXd may also extend to [redacted] population and this warrants further evaluation.

The similar efficacy results in HER2 IHC 2+ population were seen in another anti-HER2 ADC, RC-48, which is a novel recombinant human anti-HER2 monoclonal antibody conjugated with a microtubule inhibitor (monomethyl auristatin E) via a cleavable linker. In a

single-arm, open-label, phase II trial, which enrolled 125 HER2 IHC 3+ or IHC 2+ (regardless of ISH status) locally advanced or metastatic gastric or GEJ adenocarcinoma subjects who have had progression or intolerance following receipt of at least two systemic chemotherapy, the ORR of RC-48 was 24.8% (95% CI: 17.5%-33.3%), and showed similar ORR (FAS) in IHC 2+ (23.0% [95% CI: 13.2, 35.5]) and IHC 3+ population (26.6% [95% CI: 16.3, 36.1]).

On the above basis, this study will also evaluate the efficacy and safety of the HER2-expessing (IHC 3+ or IHC 2+) patients as a secondary endpoint in addition to the primary population of the HER2-positive patients being studied.

Objectives and Endpoints

Objectives	Endpoints
Primary in HER2-positive (IHC 3+ or IHC 2+/ISH +) population	
To evaluate the efficacy of T-DXd by assessment of confirmed ORR by ICR in participants with HER2-positive advanced gastric or GEJ adenocarcinoma who have received at least 2 prior regimens including a fluoropyrimidine agent and a platinum agent	Confirmed ORR is defined as the proportion of participants who have a confirmed CR or confirmed PR, as determined by ICR per RECIST 1.1.<br>The analysis will include enrolled participants with HER2-positive status (defined as IHC 3+ or IHC 2+/ISH +) confirmed by central laboratory.<br>Data obtained from enrolment up until progression, or the last evaluable assessment in the absence of progression, will be included in the assessment of ORR, regardless of whether the participant withdraws from therapy. Participants who discontinue study intervention without a response or progression, receive a subsequent anticancer therapy, and then respond will not be included as responders in the ORR.<br>The measure of interest is the estimate of ORR.
Secondary in HER2-expressing (IHC 3+ or IHC 2+) population	
To evaluate the efficacy of T-DXd by assessment of confirmed ORR by ICR in participants with HER2-expressing (IHC 3+ or IHC 2+) advanced gastric or GEJ adenocarcinoma who have received at least 2 prior regimens including a fluoropyrimidine agent and a platinum agent	Confirmed ORR is defined as the proportion of participants who have a confirmed CR or confirmed PR, as determined by ICR per RECIST 1.1.<br>The analysis will include enrolled participants with HER2-expressing status (defined as IHC 3+ or IHC 2+) confirmed by central laboratory.

Objectives	Endpoints
	Data obtained from enrolment up until progression, or the last evaluable assessment in the absence of progression, will be included in the assessment of ORR, regardless of whether the participant withdraws from therapy. Participants who discontinue study intervention without a response or progression, receive a subsequent anticancer therapy, and then respond will not be included as responders in the ORR.<br>The measure of interest is the estimate of ORR.
Secondary in HER2-positive (IHC 3+ or IHC 2+/ISH +) / HER2-expressing (IHC 3+ or IHC 2+) population	
To evaluate the efficacy of T-DXd by assessment of confirmed ORR by investigator in participants with HER2-positive / HER2-expressing advanced gastric or GEJ adenocarcinoma who have received at least 2 prior regimens including a fluoropyrimidine agent and a platinum agent	Confirmed ORR is defined as the proportion of participants who have a confirmed CR or confirmed PR, as determined by the investigator at local site per RECIST 1.1
To evaluate the efficacy of T-DXd by assessment of PFS in participants with HER2-positive / HER2-expressing advanced gastric or GEJ adenocarcinoma who have received at least 2 prior regimens including a fluoropyrimidine agent and a platinum agent	PFS is defined as time from date of enrolment until progression per RECIST 1.1 as assessed by ICR or death due to any cause.<br>PFS will be evaluated based on ICR and on investigator assessment.
To evaluate the efficacy of T-DXd by assessment of DCR in participants with HER2-positive / HER2-expressing advanced gastric or GEJ adenocarcinoma who have received at least 2 prior regimens including a fluoropyrimidine agent and a platinum agent	DCR is defined as the percentage of participants who have a confirmed CR/PR or SD (without subsequent anticancer therapy) after date of enrolment.<br>DCR will be evaluated based on ICR and on investigator assessment.
To evaluate the efficacy of T-DXd by assessment of DoR in participants with HER2-positive / HER2-expressing advanced gastric or GEJ adenocarcinoma who have received at least 2 prior regimens including a fluoropyrimidine agent and a platinum agent	DoR is defined as the time from the date of first documented response until date of documented progression or death in the absence of disease progression.<br>DoR will be evaluated based on ICR and on investigator assessment.
To evaluate the efficacy of T-DXd by assessment of OS in participants with HER2-positive / HER2-expressing advanced gastric or GEJ adenocarcinoma who have received at least 2 prior regimens including a fluoropyrimidine agent and a platinum agent	OS is defined as time from date of enrolment until the date of death due to any cause
To evaluate efficacy of T-DXd by assessment of tumour size change at each DCO in participants with HER2-positive /	Tumour size change is defined as the best percentage change from baseline in the

Objectives	Endpoints
HER2-expressing advanced gastric or GEJ adenocarcinoma who have received at least 2 prior regimens including a fluoropyrimidine agent and a platinum agent	sum of diameters of target lesions at each DCO.<br>Tumour size change will be evaluated based on ICR and investigator assessment.
PK and immunogenicity	
To assess the PK of T-DXd in participants with HER2 expressing advanced gastric or GEJ adenocarcinoma who have received at least 2 prior regimens including a fluoropyrimidine agent and a platinum agent	Concentrations of T-DXd, total anti-HER2 antibody, and MAAA-1181a in serum and evaluation of appropriate PK parameters
To investigate the immunogenicity of T-DXd in participants with HER2 expressing advanced gastric or GEJ adenocarcinoma who have received at least 2 prior regimens including a fluoropyrimidine agent and a platinum agent	Presence of ADAs against T-DXd in serum during treatment (before infusion on Day 1 of Cycles 1, 2, and 4, and every 4 cycles thereafter) and at follow-up. Neutralising ADAs will also be assessed
Safety	
To assess the safety and tolerability of T-DXd in participants with HER2-expressing advanced gastric or GEJ adenocarcinoma who have received at least 2 prior regimens including a fluoropyrimidine agent and a platinum agent	Safety and tolerability will be evaluated in terms of SAEs, TEAEs, AESI, (including discontinuations due to AEs and due to AESI), vital signs, clinical laboratory results, ECGs, physical examination findings, elevated troponin levels, ECHO/MUGA findings, ophthalmologic findings, and ADAs

ADAs = anti-drug antibodies; AE = adverse event; AESI = adverse event of special interest; CR = complete response; DCO = data cut off; DCR = disease control rate; DoR = duration of response; ECG = electrocardiogram; ECHO = echocardiogram; GEJ = gastroesophageal junction; HER2 = human epidermal growth factor receptor 2; ICR = independent central review; IHC = immunohistochemistry; ISH = in situ hybridisation; MUGA = multigated acquisition scan; ORR = objective response rate; OS = overall survival; PFS = progression-free survival; PK = pharmacokinetics; PR = partial response; RECIST 1.1 = Response Evaluation Criteria in Solid Tumours version 1.1; SAE = serious adverse event; SD = stable disease; T-DXd = trastuzumab deruxtecan; TEAE = treatment-emergent adverse event.

Overall Design

Disclosure Statement: This is a Phase II, open-label, single-arm, multicenter, study in China assessing the efficacy and safety of T-DXd in participants with HER2-expressing advanced gastric or GEJ adenocarcinoma who have received at least 2 prior regimens including a fluoropyrimidine agent and a platinum agent.

Participant Population:

The target population of interest in this study is participants with HER2-positive (defined as IHC 3+ or IHC 2+/ISH +) advanced gastric or GEJ adenocarcinoma who have received at least 2 prior regimens including a fluoropyrimidine agent and a platinum agent. The study will enroll participants who have HER2-expressing (defined as IHC 3+ or IHC 2+) status, in which

efficacy and safety will be evaluated as secondary endpoints. Participant eligibility with respect to HER2 status will be determined locally, however, the analyses of primary and secondary efficacy endpoints will be performed on participants whose HER2-positive/expressing status is confirmed by central laboratory.

Number of Participants:

Approximately [redacted] will be screened in order to enroll approximately [redacted] participants with HER2-expressing (IHC 3+ or IHC 2+, based on local laboratory report) advanced gastric or GEJ adenocarcinoma who have received at least 2 prior regimens including a fluoropyrimidine agent and a platinum agent to study intervention. This is to ensure that approximately [redacted] participants enrolled in the study have HER2-positive (IHC3+, or IHC2+/ISH +) status confirmed by central laboratory as these participants will form the basis of the primary analysis population. This also provides opportunity for the secondary objective of the study which is to assess efficacy using confirmed ORR as endpoint in approximately [redacted] participants who have HER2-expressing (IHC 3+ or IHC 2+) status confirmed by central laboratory.

Assuming the true ORR (by ICR) of T-DXd is [redacted] approximately [redacted] participants with HER2-positive (defined as IHC 3+ and IHC 2+/ISH + by central laboratory) advanced gastric or GEJ adenocarcinoma will provide approximately [redacted] probability for the [redacted] of the study intervention ORR to [redacted] For the ORR in the HER2-expressing population the [redacted] and therefore the [redacted] Refer to Section 9.2 for further information.

Note: Potential participants who are screened for the purpose of determining eligibility for the study but are not enrolled, are considered "screen failures", unless otherwise specified by the protocol.

Intervention Groups and Duration:

Following confirmation of eligibility during screening (28-day screening period), all enrolled participants will be assigned to treatment with open-label T-DXd 6.4 mg/kg as an intravenous infusion once every 3 weeks, on Day 1 of each 3-week cycle. Study intervention may continue until RECIST 1.1-defined progressive disease (PD) or any other criterion for discontinuation of study intervention is met.

Study intervention (T-DXd) beyond RECIST 1.1-defined PD is not permitted in this study.

Follow-up of participants post discontinuation of study intervention:

After study intervention discontinuation, all participants will undergo an end of treatment visit (within 7 days after discontinuation) and will be followed up for safety assessments 40 (+ 7)

days after their last dose of study intervention (ie, the safety follow-up visit).

Participants who have discontinued study intervention in the absence of RECIST 1.1 defined radiological progression confirmed by investigator will be followed up with tumour assessments according to the Schedule of Activities (SoA) until RECIST 1.1 defined PD or death regardless of whether or not they start a subsequent anticancer therapy, unless they have withdrawn all consent to study-related assessments.

In addition, all participants who discontinue study intervention will be followed up for long-term safety, including survival status, every 3 months (± 14 days) from the date of the safety follow-up visit until death, withdrawal of consent, or the end of the study, as per the SoA.

See Section 6.7 for a description of assessments following study DCO.

Statistical methods

This is a single-arm, Phase II open-label study in which all participants will receive the same study intervention. The primary objective of the study is to evaluate the efficacy of T-DXd by assessing the confirmed ORR by ICR in participants with HER2-positive (defined as IHC 3+ and IHC 2+/ISH + by central laboratory) advanced gastric or GEJ adenocarcinoma who have received at least 2 prior treatment regimens. A secondary objective of the study is to evaluate the efficacy of T-DXd by assessing the confirmed ORR by ICR in participants with HER2-expressing (defined as IHC 3+ and IHC 2+ by central laboratory) advanced gastric or GEJ adenocarcinoma who have received at least 2 prior treatment regimens.

The primary endpoint of the study is confirmed ORR, defined as the number (%) of participants with confirmed CR or PR as determined by ICR per RECIST 1.1. ORR by ICR will be estimated with a 2-sided 95% exact CI.

To ensure strong control of type 1 error amongst the primary and secondary ORR endpoints in the HER2-positive and HER2-expressing populations, [redacted] will be applied to the analysis of ORR in the [redacted]. The analysis of ORR in the [redacted] See Section 9.4.5 for details.

The DCO for the primary analysis of ORR by ICR will occur approximately [redacted] months after the last participant has initiated study intervention. Duration of response (DoR), disease control rate (DCR), tumour size change, and available safety, tolerability, and pharmacokinetic (PK) data will also be summarised at this time.

The DCO for the full final analysis of ORR by ICR will occur approximately [redacted] months after the last participant has initiated study intervention. Based on data from Study DESTINY-

Gastric01, [redacted] months is sufficient time for participants to reach a response and to allow DoR to be determined for responders. The full final analysis will report the analyses of all primary and secondary endpoints, including updated ORR and DoR, DCR, tumour size change, progression-free survival (PFS), overall survival, PK, and safety.

Safety data will be summarised descriptively and will not be formally analysed unless otherwise specified.

1.2 Schema

Figure 1 Study Design

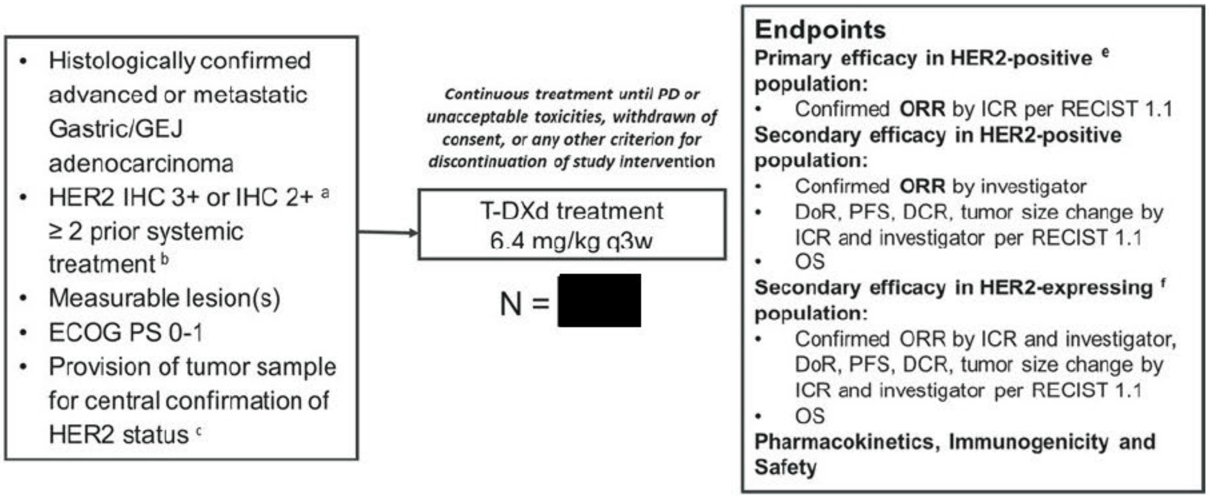

a For the purposes of eligibility, HER2-positivity will be determined locally.
b Prior systemic treatment must contain fluorouracil and platinum.
c If the primary tumour is accessible by endoscopy or if metastatic tissue can be biopsied, a newly-acquired tumour tissue sample should be provided. If a newly acquired sample is not available, an archived sample is acceptable and should be based on the most recent archived tumour tissue sample.
d N = [redacted] participants who are HER2 IHC 3+ or IHC 2+ based on local laboratory report will be enrolled, to ensure that approximately [redacted] participants enrolled in the study have HER2-positive status confirmed by central laboratory as these participants will form the basis of the primary analysis population. This also provides that approximately [redacted] participants enrolled in the study have HER2-expressing status confirmed by central laboratory as these participants will form the basis of the analysis population for the secondary objectives in this population.
e HER2-positive is defined as IHC 3+ or IHC 2+/ISH +.
f HER2-expressing is defined as IHC 3+ or IHC 2+ (regardless of ISH status).

DCR = disease control rate; DoR = duration of response; ECOG PS = Eastern Cooperative Oncology Group Performance Status; GEJ = gastroesophageal junction; HER2 = human epidermal growth factor receptor 2; IHC = immunohistochemistry; ISH = in situ hybridisation; ICR = independent central review; ORR = objective response rate; OS = overall survival; PD = progressive disease; PFS = progression-free survival; q3w = every 3 weeks; RECIST 1.1 = Response Evaluation Criteria in Solid Tumours, Version 1.1; T-DXd = trastuzumab deruxtecan.

1.3 Schedule of Activities

Schedules of activities (SoA) are provided in Table 1 (screening and enrolment), Table 2 (study intervention), and Table 3 (post-study intervention follow-up and survival). Additional information is provided in Sections 1.3.1, 1.3.2, and 1.3.3, respectively. Additional general information is provided below:

- If vital signs and blood draws are scheduled for the same nominal time, vital signs should be assessed before blood draws.
- If electrocardiograms (ECGs), vital signs, and blood draws are scheduled for the same nominal time, the assessments should occur in the following order: ECG, vital signs, blood draws. The timing of the first 2 assessments should be such that it allows the blood draw to occur at the timepoints indicated in the SoA.
- On visits where participants do not receive study intervention, they should undergo a physical examination and vital signs assessment with additional assessments at the discretion of the investigator.

1.3.1 Screening and Enrolment Period

Screening and enrolment procedures are presented in the SoA (Table 1).

After signing the main informed consent form (ICF), participants will begin screening procedures. Screening will take place in the 28 days prior to enrolment. The SoA (Table 1) indicates which screening procedures should be completed in the 14 days prior to enrolment.

Eligible participants must have documented HER2 status as IHC 3+ or IHC 2+. For the purposes of determining study eligibility during screening, HER2-positivity should be determined at site based on a documented pathology result obtained from either a local laboratory or from a previous pathology report that has been confirmed by the investigator.

HER2 status will subsequently be confirmed by a central laboratory but the result of central HER2 testing will not determine study eligibility. To enable confirmation of HER2 status by central laboratory, an adequate newly acquired tumour sample should be provided during screening for all participants where the primary tumour is accessible by endoscopy or if metastatic tissue can be biopsied. If a newly acquired sample is not available, an archived sample is acceptable and should be based on the most recent archived tumour tissue sample.

Following completion of all screening procedures and documentation of all baseline assessments, eligible participants will be enrolled in the study. The date of enrolment is defined as the date the participant is confirmed as eligible in the interactive response technology (IRT). Thus, a treatment assignment number should be obtained from the IRT on the day the participant's eligibility is confirmed and they are enrolled in the study.

Participants who fail to meet the eligibility criteria will be termed "screen failures". Screen

failures may be rescreened once (see Section 5.4).

Every effort should be made to minimize the time between the completion of screening/baseline assessments and the first dose of study intervention (no more than 3 days). If it is anticipated that dosing cannot occur within 3 days, a discussion with the AstraZeneca study physician is required.

1.3.2 Study Intervention Period

Procedures to be conducted during the study intervention period until the end of treatment are presented in the SoA (Table 2). Participants must not receive study intervention unless all eligibility criteria have been met.

1.3.3 Post-study Intervention Follow-up Period

Procedures to be conducted during the post-study intervention follow-up period (including survival assessments) are presented in the SoA (Table 3).

Table 1 Schedule of Activities: Screening and Enrolment

Procedures	Screening/Baseline (Days -28 to -1)	Notes	For Details, see Section or Appendix:
Written informed consent [a]	X		5.1 and A 3
Contact IRT to obtain E-code	X	Obtain E-code once participant signs the ICF	6.3
**Study Procedures and Assessments**			
Documentation of HER2-positivity for eligibility [b]	X	HER2-positivity should be determined at site based on a documented pathology result obtained from either a local laboratory or from a previous pathology report that has been confirmed by the investigator	5.1 and 8.6.1
Provision of mandatory tumour sample for confirmation of HER2 status by central laboratory [c]	X	Tissue sample for central laboratory confirmation of HER2 status must be provided after enrolment and prior to first dose of study intervention	5.1 and 8.6.1
Inclusion and exclusion criteria	X	Re-check prior to first dose of study intervention	5.1 and 5.2
Demography	X		5.1
Full physical examination	X [d]		8.2.1
Height and weight	X [d]		8.2.1
Medical history (includes substance usage and family history of premature cardiovascular disease)	X	Include: drugs, alcohol, caffeine, and tobacco (including history [dates], type, and frequency of tobacco use, e-cigarette use, vaping)	5.1, 5.2 and 5.3
Primary cancer history	X	Include prior anticancer therapy	5.1 and 5.2
**Tumour Assessment**			
Tumour imaging (RECIST 1.1)	X		8.1.1
**Safety Assessments**			
ECOG performance status	X [d]		8.2.5.3
12-lead ECG	X [d]	Performed in triplicate completed within 5 minutes	8.2.3
Vital signs	X [d]		8.2.2
ECHO or MUGA	X		8.2.5.1
**Lung Function**			
Pulmonary function tests	X		8.2.5.2
SpO_2	X		8.2.5.2

Procedures	Screening/Baseline (Days -28 to -1)	Notes	For Details, see Section or Appendix:
High-resolution computed tomography scan	X		8.2.5.2
**Laboratory Assessments**			
Serum pregnancy test (WOCBP only)	X	Negative serum pregnancy test must be available within 72 hours prior to enrolment for WOCBP	5.1, 5.2, and 8.2.4
Hepatitis B/C serology[f]	X		5.2 and 8.2.4
HIV antibody test (as required by local regulations or IEC)	X		5.2 and 8.2.4
Clinical safety laboratory assessments (clinical chemistry, haematology)	X [d]	Clinical laboratory assessments must be completed within 14 days prior to enrolment. See inclusion criterion 8	8.2.4
Coagulation	X [d]	Coagulation tests performed only at screening include PT/INR and PTT/aPTT	8.2.4
Urinalysis	X [d]		8.2.4
Troponin	X [d]		8.2.3 and 8.2.4
**Other Safety Assessments**			
Ophthalmologic assessments	X		8.2.5.4
Adverse events	X	Collect AEs and SAEs from provision of informed consent	8.3
Concomitant therapy	X		6.5
**Treatment Assignment**			
Contact IRT to obtain treatment assignment number on the day the participant is enrolled (ie, confirmed as eligible)	X [e]	Once a participant has completed screening and been confirmed as eligible	6.3

[a] Written informed consent and any locally required privacy act document authorisation must be obtained prior to performing any protocol-specific procedures, including screening/baseline evaluations.

[b] If the participant's HER2 status has not been documented previously, a tissue sample must be provided for local HER2 status testing. If the primary tumour is accessible by endoscopy or if metastatic tissue can be biopsied, this should be a newly-acquired tumour tissue sample. If a newly acquired sample is not available, an archived sample is acceptable and should be based on the most recent archived tumour tissue sample. Providing the sample should be given the highest priority so eligibility can be confirmed within the 28-day screening period. See Section 8.6.1.

[c] All participants must provide a tumour sample for confirmation of HER2 status by central laboratory. If the primary tumour is accessible by endoscopy or if metastatic tissue can be biopsied, this should be a newly-acquired tumour tissue sample. If a newly acquired sample is not available, an archived sample is acceptable and should be based on the most recent archived tumour tissue sample. The results of central laboratory testing are not used to determine participant eligibility. See Section 8.6.1.

[d] Assessment must be completed within the 14 days prior to enrolment, ie, Days -14 to -1.

[e] Participants may only be confirmed as eligible and enrolled into the study once all screening procedures have been completed.

[f] HBsAg; anti-HBc (IgG or total Ig) for all patients. HBeAg and HBV DNA are additionally required if HBsAg positive; anti-HBs and HBV DNA are additionally required if HBsAg negative, anti-HBc positive, at screening (See Section 8.2.4 Clinical Safety Laboratory Assessments)

AE = adverse event; aPTT = activated partial thromboplastin time; ECG = electrocardiogram; ECHO = echocardiogram; ECOG = Eastern Cooperative Oncology Group; HER2 = human epidermal growth factor receptor 2; HIV = human immunodeficiency virus; ICF = informed consent form; IEC = independent ethics committee; INR = international normalised ratio; IRT = interactive response technology; MUGA = multigated acquisition; PT = prothrombin time; PTT = partial thromboplastin time; RECIST 1.1 = Response Evaluation Criteria in Solid Tumours, Version 1.1; SAE = serious adverse event; SpO_2 = pulse oximetry; WOCBP = women of childbearing potential.

Table 2 Schedule of Activities: Study Intervention Period

Procedure	Study Intervention						EOT or ED	Notes	For Details, see Section or Appendix
	Cycle 1			Cycle 2	Cycle 3	Cycle 4 and subsequent cycles until PD			
**Day**	**1**	**8**	**15**	**1**	**1**	**1**			
**Visit Window (days)**		**± 1**	**± 1**	**± 2**	**± 2**	**± 2**	**+ 7**		
**Study Procedures and Assessments**									
Inclusion and exclusion criteria	X							Re-check clinical status before first dose of study intervention	5.1 and 5.2
Targeted physical examination	X [a]	X	X	X [a]	X [a]	X [a]	X		8.2.1
Weight	X [a]			X [a]	X [a]	X [a]	X		8.2.1
**Tumour Assessment**									
Tumour imaging (RECIST 1.1)	Every 6 weeks (± 7 days) from first dose of study intervention until RECIST 1.1-defined radiological PD								8.1.1
**Safety Assessments**									
ECOG performance status	X [a]			X [a]	X [a]	X [a]	X		8.2.5.3
12-lead ECG[d]	X [a]					X [a] (C5 and every 4 cycles thereafter)	X	Single ECG trace, unless abnormalities noted	8.2.3
Vital signs	X [a]	X	X	X [a]	X [a]	X [a]	X	Before and at end of infusion on Day 1 of each cycle	8.2.2
ECHO or MUGA[e]						X [a] (C5 and every 4 cycles thereafter)	X		8.2.5.1

Table 2 Schedule of Activities: Study Intervention Period

Procedure	Study Intervention						EOT or ED	Notes	For Details, see Section or Appendix
	Cycle 1			Cycle 2	Cycle 3	Cycle 4 and subsequent cycles until PD			
**Day**	**1**	**8**	**15**	**1**	**1**	**1**			
**Visit Window (days)**		**± 1**	**± 1**	**± 2**	**± 2**	**± 2**	**+ 7**		
**Lung Function**									
SpO_2	X [a]	X	X	X [a]	X [a]	X [a]	X	Before and at end of infusion on Day 1 of each cycle	8.2.5.2
Pulmonary function tests	If ILD/pneumonitis is suspected								8.2.5.2
High-resolution computed tomography [c]	If ILD/pneumonitis is suspected								8.2.5.2
ILD/pneumonitis investigation	If ILD/pneumonitis is suspected								8.2.5.2.1
**Laboratory Assessments**									
Serum or urine pregnancy test (WOCBP only)	X			X	X	X	X	Negative pregnancy test required within 72 hours prior to each dose of study intervention	5.1, 5.2, and 8.2.4
Clinical safety laboratory assessments (clinical chemistry, haematology)	X [a]	X	X	X [a]	X [a]	X [a]	X		8.2.4
Laboratory tests<br>Eligible HBV infection only [g]				X		X(alternate cycles)	X	**X q3 mo F/U**<br>(± 14 days)	8.2.5.5
Coagulation	As clinically indicated								8.2.4
Urinalysis	As clinically indicated								8.2.4
Troponin	Whenever a participant reports signs or symptoms suggesting CHF, MI, or other causes of myocyte necrosis						X		8.2.3 and 8.2.4
**Other Safety Assessments**									
Ophthalmologic assessments	As clinically indicated						X		8.2.5.4

Table 2 Schedule of Activities: Study Intervention Period

Procedure	Study Intervention						EOT or ED	Notes	For Details, see Section or Appendix
	Cycle 1			Cycle 2	Cycle 3	Cycle 4 and subsequent cycles until PD			
**Day**	**1**	**8**	**15**	**1**	**1**	**1**			
**Visit Window (days)**		**± 1**	**± 1**	**± 2**	**± 2**	**± 2**	**+ 7**		
Adverse events [f]	At every visit. May be conducted by phone if not tied to a visit							Collect AEs/SAEs throughout study intervention, until completion of safety follow-up visit (see Table 3)	8.3
Concomitant therapy	At every visit and may be conducted by phone if not tied to a visit								6.5
**Pharmacokinetic and Immunogenicity Assessments**									
Pre-dose blood sample for T-DXd PK testing	X			X	X	X (Cycles 4, 6, and 8)		Take samples within 8 hours before infusion	8.5.1
End of infusion blood sample for T-DXd PK testing	X			X	X	X (Cycle 4)		Take samples within 15 minutes after end of infusion	8.5.1
5 hours post-dose blood sample for T-DXd PK testing	X							Take sample within 5 (± 2) hours after start of infusion (C1 D1)	8.5.1
Additional blood sample for T-DXd PK testing (if feasible)	If ILD/pneumonitis is suspected								8.5.1
Pre-dose blood sample for immunogenicity testing	X			X		X (C4 and every 4 cycles thereafter, ie, Cycles 4, 8, 12, etc)		Take samples within 8 hours before infusion at time points indicated	8.5.2
**Study Intervention Administration [b]**									

Table 2 Schedule of Activities: Study Intervention Period

Procedure	Study Intervention						EOT or ED	Notes	For Details, see Section or Appendix
	Cycle 1			Cycle 2	Cycle 3	Cycle 4 and subsequent cycles until PD			
**Day**	**1**	**8**	**15**	**1**	**1**	**1**			
**Visit Window (days)**		± 1	± 1	± 2	± 2	± 2	+ 7		
T-DXd [b]	6.4 mg/kg intravenous infusion on Day 1 of each 3-week cycle							Do not co-administer other drugs through the same infusion line	6 and 7

[a] Within 3 days prior to administration of study intervention.

[b] Every effort should be made to minimise the time between enrolment (confirmation of eligibility) and starting study intervention (ie, within 3 days).

[c] HRCT is preferred and recommended, CT Scan should be used only when HRCT is not available.

[d] ECG will be taken in triplicate before infusion on C1D1. Subsequent ECGs will be performed in triplicate in close succession only if an abnormality is noted. ECGs will be taken while in a supine/semi-recumbent position. ECGs will then be taken at every 4th cycle (i.e. C5, 9, 13…) If ECG is abnormal follow institutional guidelines.

[e] ECHO or MUGA scan assessments (note: the same test must be used for the subject throughout the study) will be performed before infusion on Day 1 of Cycle 5 and then every 4 cycles (within7 days of IMP administration) (e.g. Cycle 5, 9, 13…)

[f] All adverse events and SAEs (other than ILD/pneumonitis and reactivation of Hepatitis B) will be collected from the time of signature of the ICF throughout the treatment period and including the safety follow-up (which is 40 +7 days after the discontinuation of all IPs or until initiation of another therapy). For ILD/pneumonitis or Hepatitis B reactivation, safety follow up will be continued until resolution of ILD/pneumonitis or Hepatitis B reactivation. If an event that starts post the defined safety follow up period noted above is considered to be due to a late onset toxicity to study treatment, including reactivation in eligible patients with Hepatitis B infection, then it should be reported as an AE or SAE as applicable.

[g] For eligible patients with HBsAg (-), anti-HBc (+) (IgG or total Ig) serology: HBsAg, ALT, and HBV DNA should be measured within 7 days before administration of cycle 2 and alternate cycles thereafter. For eligible patients with HBsAg (+), and HBV DNA <2000IU: ALT and HBV DNA should be measured within 7 days before administration of cycle 2 and alternate cycles thereafter. For all eligible HBV patients, if study medication is interrupted monitoring should be continued at least 3–monthly or if study medication is discontinued, monitoring should be continued at least 3-monthly for at least 12 months. (See Section 8.2.5.5 Safety assessment for Eligible HBV infection only)

Note: All assessments on study intervention days are to be performed prior to study intervention administration, unless otherwise indicated. Data collection following study analysis until the end of the study is described in Section 8.
AE = adverse event; C = cycle; CHF = congestive heart failure; D = day; ECG = electrocardiogram; ECHO = echocardiogram; ECOG = Eastern Cooperative Oncology Group; ED = Early Study Intervention/Discontinuation; EOT = End of Treatment; ILD = interstitial lung disease; MI = myocardial infarction; MUGA = multigated acquisition; PD = progressive disease; PK = pharmacokinetic; RECIST 1.1 = Response Evaluation Criteria in Solid Tumours, Version 1.1; SAE = serious adverse event; SpO_2 = pulse oximetry; T-DXd = trastuzumab deruxtecan; WOCBP = women of childbearing potential.

Table 3 Schedule of Activities: Post-study Intervention Follow-up and Survival

Procedures	Post-intervention Follow-up Period		Notes	For Details, see Section
	Safety Follow-up	Long-term Follow-up		
**Days**	**40 days after last dose**	**Every 3 months**		
**Window**	**+ 7 days**	**± 14 days**		
Targeted physical examination	X			8.2.1
Weight	X			8.2.1
Subsequent anticancer therapy, including surgery and radiation therapy	X	X		7.1.1
**Tumour Assessment**				
Tumour imaging (RECIST 1.1)	Every 6 weeks (± 7 days) from first dose of treatment until RECIST 1.1-defined PD		All participants should have IV contrast-enhanced CT or MRI of the chest, abdomen, and pelvis every 6 weeks (± 7 days) until RECIST 1.1-defined radiological PD<br>Participants who discontinue study intervention due to reasons other than PD or death (regardless of whether they have started subsequent anticancer therapy): IV contrast-enhanced CT or MRI of the chest, abdomen, and pelvis every 6 weeks (± 7 days) for the first 48 weeks after the date of the first dose of study intervention, then every 12 weeks (± 7 days) thereafter, until RECIST 1.1-defined radiological PD	8.1.1
**Safety Assessments**				
ECOG performance status	X			8.2.5.3
Vital signs	X			8.2.2
SpO_2	X			8.2.5.2
**Laboratory Assessments**				
Serum or urine pregnancy test (WOCBP only)	X			8.2.4
Clinical safety laboratory assessments (clinical chemistry, haematology)	X			8.2.4
Coagulation	As clinically indicated			8.2.4
Urinalysis	As clinically indicated			8.2.4

Procedures	Post-intervention Follow-up Period		Notes	For Details, see Section
	Safety Follow-up	Long-term Follow-up		
**Days**	**40 days after last dose**	**Every 3 months**		
**Window**	**+ 7 days**	**± 14 days**		
**Other Safety Assessments**				
Adverse events	At every visit. May be conducted by phone if not tied to a visit		Collect AEs/SAEs until completion of SFU visit. Follow-up events of ILD/pneumonitis to resolution. If an event that starts after the SFU visit is considered due to a late onset toxicity to study intervention, report as an AE/SAE, as applicable	8.3
Concomitant therapy	At every visit. May be conducted by phone if not tied to a visit			6.5
**Immunogenicity Assessments**				
Blood sample for immunogenicity testing	X [a]		[a] Take SFU visit sample at 40 (+ 7 days) after last study intervention or before starting new anticancer treatment, whichever occurs first	8.5.2
**Other Assessments**				
Survival status	X	X	Until death, withdrawal of consent, or end of study	7.1.2

ADA = anti-drug antibody; AE = adverse event; CT = computed tomography; ECOG = Eastern Cooperative Oncology Group; ILD = interstitial lung disease; IV = intravenous; MRI = magnetic resonance imaging; PD = progressive disease; RECIST 1.1 = Response Evaluation Criteria in Solid Tumours, Version 1.1; SAE = serious adverse event; SFU = safety follow-up; SpO_2 = pulse oximetry; WOCBP = women of childbearing potential.

2 INTRODUCTION

Trastuzumab deruxtecan (T-DXd; also known as DS-8201a) is a HER2-targeting antibody-drug conjugate (ADC) in development as a candidate therapy for breast cancer and other HER2-expressing tumours. The non-proprietary name for T-DXd is trastuzumab deruxtecan except in the United States where it is fam-trastuzumab deruxtecan-nxki.

2.1 Study Rationale

Human epidermal growth factor receptor 2 (HER2) is an important target protein for gastric cancer (GC) therapy (Huang et al 2013). HER2 expression initiates a strong pro-tumourigenic signalling cascade and it has been shown that HER2-positive status is associated with decreased survival and clinicopathological features of tumour progression, such as invasion, metastases, and higher disease stage (Gravalos and Jimeno 2008). HER2 testing is therefore recommended for all gastric adenocarcinoma patients at the time of diagnosis if metastatic disease is documented or suspected. Immunohistochemistry (IHC) and in situ hybridisation (ISH) are the gold standard methods for HER2 testing.

HER2-positive was refined by Hofmann et al in 2008 (Bang et al 2010, Hofmann et al 2008) and was validated in a subsequent study (Ruschoff et al 2010). A score of 0 or 1+ is considered to be HER2-negative. A score of 2+ is considered equivocal and should be additionally examined by ISH methods. Cases that have an IHC score of 3+ or an IHC score 2+ and ISH positive are considered HER2-positive. Across Asian populations, HER2 positivity (defined as IHC 3+ and IHC 2+/ISH +) in GC is reported in approximately 9.8% to 23.0% of cases (Cho et al 2013, Kim et al 2011, Park et al 2006, Shan et al 2013, Yano et al 2006, Yoon et al 2018). In China, HER2-positivity is reported in 12% to 13% of patients with GC (Sheng et al 2013,Huang et al 2013). Although ISH testing is recommended to verify HER2 status for IHC 2+ patients, the testing rate of ISH in the IHC 2+ population in China is very low (<10%) (Huang et al 2018). Regardless of ISH results, there are 22% to 24% of GC patients in China have HER2 expressing (defined as IHC 3+ or IHC 2+ in this study) status (Sheng et al 2013, Fan et al 2013, Shan et al 2013).The prognostic significance of HER2 status in GC remains to be fully elucidated. While some studies suggest that HER2-positivity is associated with poor prognosis (Cho et al 2013, Chua and Merrett 2012, Gomez-Martin et al 2012, Gravalos and Jimeno 2008, Jorgensen and Hersom 2012, Kato et al 2018), others report that HER2 could be an independent prognostic factor of outcome in a subgroup of patients with intestinal histology (Grabsch et al 2010, Janjigian et al 2012, Kunz et al 2012). GC is characterised as a tumour with a high degree of heterogeneity, such as in histology (differentiated or undifferentiated type) and site of tumour progression (hepatic or peritoneal metastases); heterogeneity in HER2-expression between metastatic sites and within the tumour has also been observed, and this is one of the key hurdles for the development of anti-HER2 drug for GC (Lee et al 2013, Ruschoff et al 2012,Thuss-Patience et al 2017). In addition, a decrease in HER2-expression after anti-HER2 treatment has been reported in

approximately 30% to 60% of HER2-positive GC patients (Pietrantonio et al 2016, Saeki et al 2018).

The prognosis of metastatic GC is poor. After relapse or progression, trastuzumab is the only approved HER2-targeted therapy and is indicated, in combination with cisplatin and capecitabine or 5-fluorouracil for the treatment of patients with HER2-positive metastatic gastric or gastroesophageal junction (GEJ) adenocarcinoma who have not received prior treatment for metastatic disease. However, after treatment failure of trastuzumab, there is no further antiHER2-specific treatment available in second line setting in China, treatment options in these patients are limited to single agent chemotherapy, followed by third-line treatment (regardless of HER2 expression status) with apatinib, nivolumab or single agent chemotherapy, or participation in a clinical trial (Li et al 2018), or with a recently (June 2021) NMPA conditionally approved (not recommended in guidelines) anti-HER2 ADC RC-48.

Given that the majority of patients with HER2-positive advanced GC eventually acquire resistance or show intolerance to drugs approved for use in the first- or second-line settings and that there is no recommended HER2-specific treatment available beyond the first-line setting, HER2-positive advanced GC is considered a serious and life-threatening disease and this patient population has a high unmet medical need.

In DESTINY-Gastric01 and DESTINY-Gastric02 studies, T-DXd showed promising efficacy in Korean and Japanese patients (confirmed ORR by ICR was 40.5% [95% CI: 31.8, 49.6] in T-DXd group vs 11.3% [95% CI: 4.7, 21.9] in the comparator group ($p < 0.0001$) (Shitara et al 2020) in addition to US and European patients (confirmed ORR was 38% [95% CI, 27.3-49.6]) (Cutsem et al 2021).

Results from Study [redacted] also showed T-DXd has benefit in [redacted] population, confirmed ORR by ICR (FAS) in [redacted] [95% CI: [redacted]]) was comparable to that in [redacted] population (N= [redacted] [95% CI: [redacted] These data suggest that the antitumor activity of T-DXd may also extend to [redacted] population and this warrants further evaluation.

The similar efficacy results in HER2 IHC 2+ population were seen in another anti-HER2 ADC, RC-48. In a single-arm, open-label, phase II trial, which enrolled 125 HER2 overexpression (defined as IHC 3+ or IHC 2+ in RC-48 label) locally advanced or metastatic gastric or GEJ adenocarcinoma subjects who have had progression or intolerance following receipt of at least two systemic chemotherapy, the ORR of RC-48 was 24.8% (95% CI: 17.5%-33.3%), and showed similar ORR (FAS) in IHC 2+ (23.0% [95% CI: 13.2, 35.5]) and IHC 3+ population (26.6% [95% CI: 16.3, 36.1]) (Peng et al 2021).

On the above basis, the current study aims to evaluate the efficacy and safety of T-DXd monotherapy in patients in China with HER2-positive (IHC 3+ or IHC 2+/ISH +, confirmed

by central laboratory), in addition to HER2-expressing (IHC 3+ or IHC 2+, confirmed by central laboratory) locally advanced or metastatic gastric or GEJ adenocarcinoma who have received 2 or more prior regimens in order to assess the opportunity to bring new HER2-targeted agents into this challenging setting.

2.2 Background

T-DXd is being developed for the treatment of HER2-expressing cancers and HER2-mutant tumours. Clinical observations in Phase I and II studies indicate that T-DXd has antitumour activity in HER2-expressing cancers, including GC. In China, there are currently no anti-HER2 therapies recommended for patients with GC who have failed a trastuzumab containing regimen (Li et al 2018, Treatment Guidelines 2019), demonstrating a clear unmet medical need for patients with HER2-positive GC in the third-line setting. As detailed below, this Phase II study will evaluate the efficacy and safety of T-DXd monotherapy in patients in China with HER2-positive (confirmed by central laboratory) locally advanced or metastatic gastric or GEJ adenocarcinoma who have received 2 or more prior regimens.

2.2.1 HER2-positive Gastric Cancer

Gastric cancer is the fifth most common cancer worldwide. There are approximately 950000 newly diagnosed cases and 780000 deaths annually worldwide, making GC the third leading cause of cancer-related deaths in the world (GLOBOCAN 2018a, Torre et al 2012). Globally, the highest incidence of GC is reported in East Asian countries, particularly China (with nearly half of global cases), Japan, and South Korea. In China, GC is now the second most commonly diagnosed cancer and is the second leading cause of cancer-related death (Chen et al 2016). GLOBOCAN statistics estimate that there were 456124 new cases of GC and 390182 deaths caused by GC in China in 2018 (GLOBOCAN 2018b).

Gastric cancer is often diagnosed at an advanced inoperable stage; disease-related symptoms include fatigue, nausea, vomiting, decreased appetite, abdominal pain, weight loss, and anaemia. In addition, patients might have difficulty taking oral food due to their advanced disease, leading to a reduction in quality of life. Worldwide, approximately 65% of GC patients present with locally advanced or metastatic disease, with a poor outcome (Meza-Junco and Sawyer 2012). In China, this figure is higher, with approximately 80% of patients presenting with advanced disease and likely to have a poor outcome: the 5-year relative survival rate was 35.9% in patients with GC in the period 2010-2014 (Yang et al 2018). In patients with GC who are refractory to standard therapies, the median life expectancy is approximately 6 months, underlining the high unmet medical need (Minashi and Hironaka 2015).

Over 95% of GC including GEJ are adenocarcinomas, which are typically classified based on histological features such as diffuse or intestinal subtype. The intestinal subtype is more common. A specific diagnosis of gastric adenocarcinoma should be established for staging and

treatment purposes, while sub-classification of gastric adenocarcinoma as an intestinal or diffuse subtype may have implications for therapy since intestinal subtype tumours are more likely to be HER2-positive (NCI 2019).

2.2.2 Current Treatment Options for HER2-positive Gastric Cancer

For patients with relapsed or progressed early-stage GC after gastrectomy and for those with de novo unresectable or metastatic GC, trastuzumab is the only approved HER2-targeted therapy and, as indicated is used in combination with cisplatin and capecitabine or 5-fluorouracil in patients with HER2-overexpressing metastatic gastric or GEJ adenocarcinoma who have not received prior treatment for metastatic disease.

Neither the National Comprehensive Cancer Network (NCCN) treatment guideline for GC (NCCN 2021) or relevant guidelines in China (Li et al 2018, Treatment Guidelines 2019) are supportive of continuing trastuzumab beyond the first-line treatment of HER2-positive locally advanced or recurrent GC. In the absence of an approved anti-HER2 therapy for HER2-positive GC patients who have received a trastuzumab-containing regimen, the NCCN guidelines recommend treatment with CYRAMZA®(ramucirumab) as a single agent or in combination with paclitaxel. An overall response rate (ORR) for ramucirumab in combination with paclitaxel of 28% (95% confidence interval [CI]: 23, 33), a median overall survival (OS) of 9.6 months (95% CI: 8.5, 10.8), and an OS hazard ratio (HR) of 0.807 (95% CI: 0.678, 0.962) ($p = 0.017$) versus paclitaxel alone (median OS: 7.4 months [95% CI: 6.3, 8.4]) ($p = 0.017$) has been reported in HER2-expressing patients who progressed on or after a trastuzumab-containing regimen (Li et al 2018). However, ramucirumab is not approved in China, and second-line treatment options for HER2-positive patients following progression on a trastuzumab-containing regimen are limited to single-agent chemotherapy (docetaxel, paclitaxel, and irinotecan) or clinical trial participation (Li et al 2018).

Third-line therapy is generally considered in patients with good performance status and two National Medical Products Administration (NMPA)-fully approved agents show a minimal increase in OS compared with placebo:

- Apatinib (AITAN®) is a highly selective vascular endothelial growth factor receptor 2 inhibitor, which was approved by the NMPA in October 2014 as a third-line or later therapy for late-stage GC, based on a Phase III clinical study which enrolled 273 patients who had treatment failure after second-line/subsequent-lines chemotherapeutic regimens. Compared with placebo, apatinib prolonged median progression-free survival (PFS) (2.6 vs 1.8 months, $p < 0.001$) and increased the disease control rate (DCR) (42.05% vs 8.79%, $p < 0.001$). The ORRs for the apatinib and placebo groups were 2.84% and 0%, respectively (Li et al 2016).
- Nivolumab (OPDIVO®) was approved in March 2020 by NMPA for patients with advanced or recurrent gastric or gastroesophageal adenocarcinoma who have received 2

or more systemic treatment regimens. Compared with placebo, nivolumab improved median OS (5.26 vs 4.14 months) at the 2-year follow-up (HR ratio [95% CI], 0.62 [0.51, 0.76]; p < 0.0001). The ORRs for the nivolumab and placebo groups were 11.2% and 0%, respectively (Chen et al 2020).

Except the above two agents, RC-48 (AIDIXI®) was granted by NMPA conditional marketing approval after initiation of DESTINY-Gastric06 study (in Jun 2021) for the treatment of HER2 IHC 3+ or IHC 2+ (regardless of ISH status), locally advanced or metastatic gastric or GEJ adenocarcinoma patients who have had progression or intolerance following receipt of at least two systemic chemotherapy, but has not been recommended in guidelines. The ORR of RC-48 monotherapy was 24.8% (95% CI: 17.5%-33.3%), the median PFS and OS were 4.1 months (95% CI: 3.7-4.9 months) and 7.9 months (95% CI: 6.7-9.9 months), respectively (Peng et al 2021). Confirmatory phase 3 study of RC48-007 (RC-48 vs. chemotherapy) is ongoing to further evaluate the efficacy and safety of RC-48 in 3L+ HER2 IHC 2+ or IHC 3+ GC patients.

Thus, for third-line treatment of GC patients regardless of HER2 expression status, Chinese guidelines recommend treatment with apatinib, nivolumab, single-agent chemotherapy or participation in a clinical trial (Li et al 2018). Given that no anti-HER2 therapy is recommended by NCCN or in China for GC patients who have failed a trastuzumab containing regimen (Li et al 2018, Treatment Guidelines 2019), there is a clear unmet medical need for HER2-positive GC patients who have received 2 or more prior regimens.

2.2.3 Trastuzumab Deruxtecan

As of 28 March 2019, AstraZeneca and Daiichi Sankyo Company, Limited (Daiichi Sankyo) entered into a joint global development and collaboration agreement for T-DXd, a HER2-targeting ADC that is being developed as a therapeutic candidate for HER2-expressing tumours.

Trastuzumab deruxtecan, under the tradename ENHERTU®, is approved for the treatment of patients with unresectable or metastatic HER2-positive (IHC 3+ or IHC 2+/ISH +) GC that has progressed after cancer chemotherapy (in Japan) or after trastuzumab containing chemotherapy (in the US, Israel and Singapore). Trastuzumab deruxtecan is also approved for the treatment of patients with unresectable or metastatic HER2-positive breast cancer who have received ≥ 2 prior anti-HER2-based regimens in the metastatic setting (in the US, European Union, United Kingdom, and Israel), or who have progressed after cancer chemotherapy (in Japan), or who have received prior trastuzumab emtansine treatment (in Canada).

T-DXd consists of an anti-HER2 antibody, MAAL-9001, covalently linked to ~8 molecules of MAAA-1162a (GGFG tetrapeptide cleavable linker and a topoisomerase I inhibitor

[MAAA-1181a]). The antibody MAAL-9001 has the same amino acid sequence as trastuzumab (HERCEPTIN®), and thus T-DXd is similarly targeted to HER2 expressing tumours. The drug MAAA-1181a (deruxtecan [DXd]), a derivative of exatecan, is released after internalisation and leads to apoptosis of the target tumour cells via the inhibition of topoisomerase I.

Due to incorporation of a novel linker, T-DXd achieves a higher drug-antibody ratio (DAR) of approximately 8 with homogeneous conjugation of DXd, compared with other currently approved ADCs, which have a DAR of 3 to 4 (Ogitani et al 2016). In addition, the cleavable linker in T-DXd is stable in plasma, conferring a favourable safety profile as observed in nonclinical toxicology rat and monkey studies.

T-DXd exhibits HER2 specific antitumour activity via a mechanism of action that combines the monoclonal antibody (mAb) specificity with the broad cytotoxicity of the released drug. After binding to HER2 and internalisation, T-DXd is cleaved by lysosomal enzymes preferentially expressed in tumour cells and releases the drug DXd in the cytoplasm. DXd is an exatecan derivative with greater potency than SN-38, the active metabolite of irinotecan (Ogitani et al 2016). T-DXd is expected to exhibit antitumour activity through DXd-induced apoptosis and, potentially, the antibody-dependant cellular cytotoxic activity of MAAL-9001, which leads to the inhibition of Akt phosphorylation.

There are completed and ongoing clinical trials with T-DXd, either alone or in combination, across multiple HER2-expressing tumour types including GC, breast cancer, non-small cell lung cancer, and colorectal cancer (please refer to the T-DXd Investigator's Brochure [IB] for a list of completed and ongoing trials and most recent patient exposure data).

A detailed description of the chemistry, pharmacology, mechanism of action, efficacy, and safety of T-DXd is provided in the T-DXd IB.

2.3 Benefit/Risk Assessment

The study will be performed in accordance with ethical principles that have their origin in the Declaration of Helsinki and are consistent with International Council for Harmonisation (ICH)/Good Clinical Practice (GCP), and applicable regulatory requirements.

More detailed information about the known and expected benefits and potential risks of T-DXd may be found in the IB.

2.3.1 Risk Assessment

2.3.1.1 Potential Risks of T-DXd

Based on data from clinical trials, toxicities considered to be associated with administration of T-DXd include the important identified risks of interstitial lung disease (ILD)/pneumonitis and

neutropenia (including febrile neutropenia). Other identified risks of T-DXd are infusion-related reactions (IRRs), haematological adverse events (AEs) (anaemia, leucopenia, lymphopenia, thrombocytopenia), pulmonary/respiratory AEs (cough, dyspnea, upper respiratory tract infection, epistaxis), gastrointestinal (GI) AEs (abdominal pain, constipation, diarrhoea, dyspepsia, nausea, stomatitis, vomiting), hepatic AEs (hepatic function abnormality, alanine aminotransferase [ALT], aspartate aminotransferase [AST], and alkaline phosphatase increased), skin AEs (alopecia, rash, pruritis), blood bilirubin increased, pneumonia, dry eye, dehydration, hypokalaemia, decreased appetite, dizziness, fatigue, peripheral oedema, pyrexia and headache.

Based on the available pre-clinical data, review of the cumulative literature, reported toxicities for the same class of agents, the important potential risks for T-DXd are left ventricular ejection fraction (LVEF) decrease (re-labelled as 'Left ventricular dysfunction' as the undesirable clinical outcome of LVEF reductions, in accordance with Revision 2 of the European Medicines Agency guidelines on Good Pharmacovigilance Practice. This relabelling of the risk does not affect the nature or monitoring methods of LVEF decrease as a potential risk associated with T-DXd) and embryo-foetal toxicity. Keratitis is considered a potential risk for T-DXd.

Interstitial lung disease/pneumonitis and LVEF decrease are considered to be adverse events of special interest (AESI) and are closely monitored in the T-DXd clinical development programme.

T-DXd has not been studied in participants with severe/moderate hepatic impairment or severe renal impairment.

These identified and potential risks are generally manageable through dose modification and routine clinical practice.

2.3.1.2 Potential Risks of HER2-targeted Agents

Several agents, that target HER2 and prevent its activation or heterodimerisation, have been developed and marketed for the treatment of HER2-positive cancers. These include the mAbs trastuzumab (Herceptin) and pertuzumab (PERJETA®), the ADC trastuzumab emtansine (T-DM1) (KADCYLA®), and HER1- and 2-associated tyrosine kinase inhibitors, lapatinib (TYKERB®) and neratinib (NERLYNX®). The safety profile of these HER2-targeted agents has been well described. The main safety risks identified in participants receiving HER2-targeted products are described below; these could potentially be expected to occur in participants receiving T-DXd.

Cardiotoxicity: Participants treated with trastuzumab are at increased risk for developing congestive heart failure (CHF) (New York Heart Association [NYHA] Class II to IV) or asymptomatic cardiac dysfunction, including LVEF decrease. Cardiac dysfunction, mainly

asymptomatic LVEF decrease, has also been observed with pertuzumab in combination with trastuzumab. Similarly, cardiac dysfunction has been observed in participants receiving T-DM1, at a lower incidence than in trastuzumab-treated participants. The majority of cases have been asymptomatic decreases in LVEF. Cardiac dysfunction with lapatinib has occurred mainly in participants receiving the combination of trastuzumab and lapatinib and has consisted of predominantly asymptomatic LVEF decrease.

Pulmonary toxicity: Cases of pulmonary toxicity, including ILD and pneumonitis, have been observed in participants receiving trastuzumab, T-DM1, and lapatinib. Occasionally, these cases have been severe in nature and have resulted in fatal outcomes. Risk factors associated with ILD/pneumonitis include prior or concomitant therapy with other antineoplastic therapies known to be associated with it such as taxanes, gemcitabine, vinorelbine, and radiation therapy.

Hypersensitivity/IRRs: The administration of therapeutic proteins is associated with a risk of hypersensitivity and/or IRRs. Hypersensitivity/IRRs have been reported with trastuzumab, pertuzumab, and T-DM1. These can range from mild reactions to severe anaphylactic shock with fatal outcome, as has been the case for trastuzumab.

Hepatic toxicity: Cases of hepatic toxicity have occurred with T-DM1, lapatinib, and trastuzumab. In participants receiving T-DM1, hepatic toxicity has manifested mainly as transient asymptomatic liver transaminase elevations, although serious cases of drug-induced liver failure and nodular regenerative hyperplasia have also been reported. Lapatinib has also been associated with serious cases of drug-induced liver injury (DILI).

Haematological toxicity: Haematological toxicity has been observed with all HER2-targeted therapies. Neutropenia, febrile neutropenia, leucopenia, and anaemia have occurred commonly with trastuzumab, pertuzumab, and T-DM1. Thrombocytopenia, including Grade 3 and 4, is a common occurrence in T-DM1-treated participants. Although rare, serious haemorrhagic events have been reported in the setting of thrombocytopenia. Lower rates of thrombocytopenia have also occurred with trastuzumab and pertuzumab when used in combination with chemotherapy.

Refer to Appendix K for T-DXd toxicity management guidelines (TMGs).

2.3.1.3 Potential Risks of Topoisomerase I Inhibitors

DXd is a derivative of exatecan (DX-8951f), a topoisomerase I inhibitor. Other products of the same class include irinotecan and topotecan. Exatecan is a campothecin derivative, which has previously been developed by the former Daiichi Pharmaceuticals Co, Ltd. as an anticancer therapy.

The main risks associated with the use of topoisomerase I inhibitors include haematological

and GI toxicities. Haematological toxicities, manifesting as neutropenia, febrile neutropenia, anaemia, thrombocytopenia, and pancytopenia are commonly observed. An increased risk of infections, including neutropenic colitis and neutropenic sepsis has been reported with these agents.

Diarrhoea and delayed onset diarrhoea, which can be severe and lead to dehydration, have been associated with topoisomerase I inhibitors. Other significant risks include ILD/pneumonitis, liver impairment, immune system disorders, and alopecia. Acute cholinergic syndrome, manifesting as diarrhoea and other cholinergic symptoms has been reported with irinotecan.

The safety profile of exatecan is broadly similar to the safety profile of other topoisomerase I inhibitors, with haematological toxicities and GI toxicities being the most significant groups of events.

2.3.1.4 Anti-cancer treatments and immunosuppressants in eligible patients with Hepatitis B infection.

Treatment with some anti-cancer treatments and immunosuppressants such as corticosteroids increase the risk of reactivation of Hepatitis B infection. For eligible patients with Hepatitis B infection, prophylaxis with anti-viral therapy, if not already part of ongoing therapy, may be required to be started prior to study treatments, or during treatments with moderate to high doses of corticosteroids (at doses equivalent to > 20 mg prednisolone of duration ≥ 4 weeks). Anti-viral therapy (if not already ongoing) should be started prior to or at least simultaneously with the initiation of moderate to high doses of corticosteroid treatment if anticipated to be ≥ 4 weeks duration. In the event of Hepatitis B infection reactivation, anti-cancer treatment may be interrupted, and anti-viral treatment started. Management decisions should be taken in consultation with the local Hepatitis B expert.

2.3.2 Benefit Assessment

T-DXd is under development for the treatment of HER2-expressing cancers and HER2-mutant tumours. Based on preliminary clinical observations in a Phase I study (Study DS8201-A-J101 [NCT02564900]) and in two Phase II studies (Study DS8201-A-U201 DESTINY-Breast01; NCT03248492 and the DESTINY-Gastric01; NCT03329690), T-DXd demonstrated antitumour activity in [redacted] cancers, including GC and breast cancer, and a generally acceptable safety profile in these populations. Data from the Phase II DS8201-A-U204 (DESTINY-Lung01; [NCT03505710]) study also provides encouraging preliminary evidence of antitumour activity of T-DXd in tumours with [redacted]. This observation is of particular relevance in China where there are currently no approved HER2-targeted therapies for GC patients with HER2-overexpression after first-line therapy.

2.3.3 Overall Benefit: Risk Conclusion

Taking into account the measures taken to minimize risk to participants in this study, the potential risks identified in association with T-DXd are justified by the anticipated benefits that may be afforded to participants with locally advanced or metastatic GC or GEJ adenocarcinoma.

The important identified risks associated with administration of T-DXd are ILD/pneumonitis and neutropenia, including febrile neutropenia; LVEF decrease and embryo-foetal toxicity are considered important potential risks. To specifically mitigate the incidence of pulmonary toxicities, strict inclusion/exclusion criteria have been included in this Clinical Study Protocol (CSP), prohibiting most participants with pre-existing pulmonary co-morbidities from entering the study. In addition, baseline pulmonary function tests will be performed for all participants. For haematological toxicities, the use of growth factors is allowed at the discretion of the investigator. Participants will be monitored closely throughout the study and clinical and laboratory assessments will be performed before every cycle. Toxicity management guidelines are added to assist with the management of the most commonly seen AEs (Appendix K).

The emergence of the coronavirus 2019-nCoV (COVID-19) (also referred to as severe acute respiratory syndrome coronavirus 2: SARS-CoV-2) presents a potential safety risk for study participants, therefore, several risk mitigation factors have been implemented in this study. Details regarding instructions related to COVID-19 and a more detailed description of benefit/risk considerations relevant to COVID-19 are provided in Appendix H.

T-DXd has the potential to provide meaningful clinical benefit. Considering the measures to minimise risks to participants, the benefit/risk assessment supports the proposed study.

3 OBJECTIVES AND ENDPOINTS

Study objectives and endpoints are provided in Table 4.

Table 4 Objectives and Endpoints

Objectives	Endpoints
Primary in HER2-positive (IHC 3+ or IHC 2+/ISH +) population	
To evaluate the efficacy of T-DXd by assessment of confirmed ORR by ICR in participants with HER2-positive advanced gastric or GEJ adenocarcinoma who have received at least 2 prior regimens including a fluoropyrimidine agent and a platinum agent	Confirmed ORR is defined as the proportion of participants who have a confirmed CR or confirmed PR, as determined by ICR per RECIST 1.1.<br>The analysis will include enrolled participants with HER2-positive status (defined as IHC 3+ or IHC 2+/ISH +) confirmed by central laboratory.

Table 4 Objectives and Endpoints

Objectives	Endpoints
	Data obtained from enrolment up until progression, or the last evaluable assessment in the absence of progression, will be included in the assessment of ORR, regardless of whether the participant withdraws from therapy. Participants who discontinue study intervention without a response or progression, receive a subsequent anticancer therapy, and then respond will not be included as responders in the ORR.<br>The measure of interest is the estimate of ORR.
Secondary in HER2-expressing (IHC 3+ or IHC 2+) population	
To evaluate the efficacy of T-DXd by assessment of confirmed ORR by ICR in participants with HER2-expressing (IHC 3+ or IHC 2+) advanced gastric or GEJ adenocarcinoma who have received at least 2 prior regimens including a fluoropyrimidine agent and a platinum agent	Confirmed ORR is defined as the proportion of participants who have a confirmed CR or confirmed PR, as determined by ICR per RECIST 1.1.<br>The analysis will include enrolled participants with HER2-expressing status (defined as IHC 3+ or IHC 2+) confirmed by central laboratory.<br>Data obtained from enrolment up until progression, or the last evaluable assessment in the absence of progression, will be included in the assessment of ORR, regardless of whether the participant withdraws from therapy. Participants who discontinue study intervention without a response or progression, receive a subsequent anticancer therapy, and then respond will not be included as responders in the ORR.<br>The measure of interest is the estimate of ORR.
Secondary in HER2-positive (IHC 3+ or IHC 2+/ISH +) / HER2-expressing (IHC 3+ or IHC 2+) population	
To evaluate the efficacy of T-DXd by assessment of confirmed ORR by investigator in participants with HER2-positive / HER2-expressing advanced gastric or GEJ adenocarcinoma who have received at least 2 prior regimens including a fluoropyrimidine agent and a platinum agent	Confirmed ORR is defined as the proportion of participants who have a confirmed CR or confirmed PR, as determined by the investigator at local site per RECIST 1.1
To evaluate the efficacy of T-DXd by assessment of PFS in participants with HER2-positive / HER2-expressing advanced gastric or GEJ adenocarcinoma who have received at least 2 prior regimens including a fluoropyrimidine agent and a platinum agent	PFS is defined as time from date of enrolment until progression per RECIST 1.1 as assessed by ICR or death due to any cause.<br>PFS will be evaluated based on ICR and on investigator assessment.

Table 4 Objectives and Endpoints

Objectives	Endpoints
To evaluate the efficacy of T-DXd by assessment of DCR in participants with HER2-positive / HER2-expressing advanced gastric or GEJ adenocarcinoma who have received at least 2 prior regimens including a fluoropyrimidine agent and a platinum agent	DCR is defined as the percentage of participants who have a confirmed CR/PR or SD (without subsequent anticancer therapy) after date of enrolment.<br>DCR will be evaluated based on ICR and on investigator assessment.
To evaluate the efficacy of T-DXd by assessment of DoR in participants with HER2-positive / HER2-expressing advanced gastric or GEJ adenocarcinoma who have received at least 2 prior regimens including a fluoropyrimidine agent and a platinum agent	DoR is defined as the time from the date of first documented response until date of documented progression or death in the absence of disease progression.<br>DoR will be evaluated based on ICR and on investigator assessment.
To evaluate the efficacy of T-DXd by assessment of OS in participants with HER2-positive / HER2-expressing advanced gastric or GEJ adenocarcinoma who have received at least 2 prior regimens including a fluoropyrimidine agent and a platinum agent	OS is defined as time from date of enrolment until the date of death due to any cause
To evaluate efficacy of T-DXd by assessment of tumour size change at each DCO in participants with HER2-positive / HER2-expressing advanced gastric or GEJ adenocarcinoma who have received at least 2 prior regimens including a fluoropyrimidine agent and a platinum agent	Tumour size change is defined as the best percentage change from baseline in the sum of diameters of target lesions at each DCO.<br>Tumour size change will be evaluated based on ICR and investigator assessment.
PK and immunogenicity	
To assess the PK of T-DXd in participants with HER2-expressing advanced gastric or GEJ adenocarcinoma who have received at least 2 prior regimens including a fluoropyrimidine agent and a platinum agent	Concentrations of T-DXd, total anti-HER2 antibody, and MAAA-1181a in serum and evaluation of appropriate PK parameters
To investigate the immunogenicity of T-DXd in participants with HER2-expressing advanced gastric or GEJ adenocarcinoma who have received at least 2 prior regimens including a fluoropyrimidine agent and a platinum agent	Presence of ADAs against T-DXd in serum during treatment (before infusion on Day 1 of Cycles 1, 2, and 4, and every 4 cycles thereafter) and at follow-up. Neutralising ADAs will also be assessed
Safety	
To assess the safety and tolerability of T-DXd in participants with HER2-expressing advanced gastric or GEJ adenocarcinoma who have received at least 2 prior regimens including a fluoropyrimidine agent and a platinum agent	Safety and tolerability will be evaluated in terms of SAEs, TEAEs, AESI, (including discontinuations due to AEs and due to AESI), vital signs, clinical laboratory results, ECGs, physical examination findings, elevated troponin levels, ECHO/MUGA findings, ophthalmologic findings, and ADAs

Table 4 Objectives and Endpoints

Objectives	Endpoints

ADAs = anti-drug antibodies; AE = adverse event; AESI = adverse event of special interest; CR = complete response; DCO = data cut off; DCR = disease control rate; DoR = duration of response; ECG = electrocardiogram; ECHO = echocardiogram; GEJ = gastroesophageal junction; HER2 = human epidermal growth factor receptor 2; ICR = independent central review; IHC = immunohistochemistry; ISH = in situ hybridisation; MUGA = multigated acquisition scan; ORR = objective response rate; OS = overall survival; PFS = progression-free survival; PK = pharmacokinetics; PR = partial response; RECIST 1.1 = Response Evaluation Criteria in Solid Tumours version 1.1; SAE = serious adverse event; SD = stable disease; T-DXd = trastuzumab deruxtecan; TEAE = treatment-emergent adverse event.

4 STUDY DESIGN

4.1 Overall Design

This is a Phase II, open-label, single-arm, multicentre, study in China assessing the efficacy and safety of T-DXd in participants with HER2-positive advanced gastric or GEJ adenocarcinoma who have received at least 2 prior regimens including a fluoropyrimidine agent and a platinum agent (Figure 1).

Following screening of approximately [redacted] participants, approximately [redacted] participants who have HER2-expressing (defined as IHC 3+ or IHC 2+ based on local laboratory report) will be enrolled and assigned to treatment with TDXd monotherapy at a dose of 6.4 mg/kg administered intravenously (IV) every 3 weeks until Response Evaluation Criteria in Solid Tumours, Version 1.1 (RECIST 1.1)defined- progressive disease (PD), unacceptable toxicity, withdrawal of consent, or any other criterion for discontinuation of study intervention is met. This is to ensure that approximately [redacted] participants enrolled in the study have HER2-positive (defined as IHC 3+ or IHC 2+/ISH +) status confirmed by central laboratory as these participants will form the basis of the primary analysis population. This also provides that approximately [redacted] participants enrolled in the study have HER2-expressing status (defined as IHC 3+ or IHC 2+) confirmed by central laboratory as these participants will form the basis of the analysis population for the secondary objectives in this population.

The primary endpoint of the study is confirmed ORR by independent central review (ICR) per RECIST 1.1. The study will also evaluate other measures of efficacy (investigator-assessed ORR, DCR, duration of response [DoR], PFS, OS, tumour size change) and further characterise the safety, tolerability, pharmacokinetics (PK) and immunogenicity of T-DXd in participants in China.

Efficacy assessments will be based on tumour assessments performed at screening and every 6 weeks (± 7 days) from the first dose of study intervention (Cycle 1 Day 1) until RECIST 1.1 defined radiological PD. All imaging scans (computer tomography [CT] or magnetic resonance imaging [MRI]) should be sent to the central imaging vendor selected by AstraZeneca to confirm the existence of a measurable lesion by RECIST 1.1. Scans (CT or MRI) of the chest, abdomen, and pelvis are mandatory. Brain CT or MRI is required only in cases of pre-existing brain metastases or symptoms. Imaging assessments should be conducted in the same way on each occasion.

All participants are required to have documented HER2-positivity (IHC 3+ or IHC 2+) at screening. For the purposes of study eligibility, HER2-positivity will be determined at site based on a documented pathology result obtained from either a local laboratory or from a previous pathology report that has been confirmed by the investigator.

In addition, formalin-fixed paraffin-embedded (FFPE) tumour tissue slides will be provided

for central laboratory confirmation of HER2 status after enrolment and prior to first dose of study intervention. If the primary tumour is accessible by endoscopy or if a metastatic tumour can be biopsied, FFPE tumour slides generated from a newly-acquired tumour sample will be provided to the central laboratory during screening. If it is not possible to obtain a newly-acquired tumour tissue sample, archived FFPE tumour slides are acceptable and should be based on the most recent archived tumour tissue sample.

4.1.1 Study Duration

Enrolment of participants will take place over approximately [redacted] at approximately [redacted] sites in mainland China. The data cutoff (DCO) for the full final analysis will occur approximately [redacted] months after the last participant has initiated study intervention. Thus, the total study duration will be approximately [redacted] months.

4.1.2 Duration of Study Participation

The screening period for the study is 28 days. Following completion of screening and inclusion in the study, participants will receive the study intervention (T-DXd) on Day 1 of each 3-week cycle. The number of treatment cycles is not fixed and participants may continue to receive study intervention until RECIST 1.1-defined PD, unacceptable toxicity, withdrawal of consent, or any other criterion for discontinuation of study intervention is met (see Section 7.1). An end of treatment/early discontinuation visit will be performed within 7 days of discontinuation of study intervention or early discontinuation from the study.

Participants will have a post-treatment follow-up visit 40 (+ 7) days after the last administration of study intervention. Thereafter, participants will have long-term/survival follow-up visits every 3 months (± 14 days) until death, withdrawal of consent, or study closure, whichever occurs first. Long-term/survival follow-up visits may be conducted by telephone or as site visits.

4.1.3 Study Conduct Mitigation During Study Disruptions Due to Cases of Civil Crisis, Natural Disaster, or Public Health Crisis

The guidance given below supersedes instructions provided elsewhere in this CSP and should be implemented only during cases of civil crisis, natural disaster, or public health crisis (eg, during quarantines and resulting site closures, regional travel restrictions, and considerations if site personnel or study participants become infected with COVID-19 or similar pandemic infection), which would prevent the conduct of study-related activities at study sites, thereby compromising the study site staff or the participant's ability to conduct the study. The investigator or designee should contact the study sponsor to discuss whether the mitigation plans below should be implemented.

To ensure continuity of the clinical study during a civil crisis, natural disaster, or public health crisis, changes may be implemented to ensure the safety of study participants, maintain

compliance with GCP, and minimise risks to study integrity. Where allowable by local health authorities, ethics committees, healthcare provider guidelines (eg, hospital policies), or local government, these changes may include the following options:

- Obtaining reconsent for the mitigation procedures (note, in the case of verbal reconsent, the informed consent form (ICF) should be signed at the participant's next contact with the study site).
- Rescreening: Additional rescreening for screen failure and to confirm eligibility to participate in the clinical study can be performed in previously screened participants. The investigator should confirm this with the designated study physician.

For further details on study conduct during civil crisis, natural disaster, or public health crisis, refer to Appendix I.

4.2 Scientific Rationale for Study Design

4.2.1 Rationale for Study Design

The primary population (HER2 IHC 3+ or IHC 2+/ISH +) of the current study closely follows the primary cohort in a completed multicentre, open-label Phase II study conducted by Daiichi Sankyo in Japan and South Korea (DESTINY-Gastric01; NCT03329690). In DESTINY-Gastric01, the efficacy and safety of T-DXd (6.4 mg/kg) in participants with HER2-positive (IHC 3+ or IHC 2+ / ISH +) advanced gastric or GEJ adenocarcinoma who had progressed on 2 prior regimens including a fluoropyrimidine agent, a platinum agent, and trastuzumab was compared with the physician's choice of treatment. Analysis of the primary cohort in DESTINY-Gastric01 performed once all participants had completed [redacted] of treatment or discontinued study intervention confirmed the anticipated efficacy of T-DXd in participants with advanced GC/GEJ.

The primary population (HER2 IHC 3+ or IHC 2+/ISH +) of the current study closely follows the primary cohort in a completed multicentre, open-label Phase II study conducted by Daiichi Sankyo in Japan and South Korea (DESTINY-Gastric01; NCT03329690). In DESTINY-Gastric01, the efficacy and safety of T-DXd (6.4 mg/kg) in participants with HER2-positive (IHC 3+ or IHC 2+ / ISH +) advanced gastric or GEJ adenocarcinoma who had progressed on 2 prior regimens including a fluoropyrimidine agent, a platinum agent, and trastuzumab was compared with the physician's choice of treatment. Analysis of the primary cohort in DESTINY-Gastric01 performed once all participants had completed [redacted] of treatment or discontinued study intervention confirmed the anticipated efficacy of T-DXd in participants with advanced GC/GEJ.

- As of the DCO (08 November 2019) for the primary analysis, confirmed ORR per RECIST 1.1 by ICR for the primary cohort was significantly higher in the T-DXd group

than in the physician's choice group ($p < 0.0001$). ORR was 40.5% (95% CI: 31.8, 49.6) in the T-DXd group with 51/126 participants having a best overall response of complete response (CR) (10 [7.9%] participants) or partial response (PR) (41 [32.0%] participants), and 11.3% (95% CI: 4.7, 21.9) in the physician's choice group with 7/62 participants having a best overall response of PR (7 [11.3%] participants).

- The unconfirmed ORR per RECIST 1.1 by ICR was 51.3% (95% CI: 41.9, 60.5) in the T-DXd group with 61/119 participants having a BOR of CR (11 [9.2%] participants) or PR (50 [42.0%] participants), and 14.3% (95% CI: 6.4, 26.2) in the physician's choice group with 8/56 participants having a BOR of PR (8 [14.3%] participants) ($p > 0.0001$).
- The confirmed ORR per RECIST 1.1 by ICR in response evaluable participants was 42.9% (95% CI: 33.8, 52.3) in the T-DXd group, with 51/119 participants having a BOR of CR (10 [8.4%] participants) or PR (41 [34.5%] participants), and 12.5% (95% CI: 5.2, 24.1) in the physician's choice group, with 7/56 participants having a BOR of PR (7 [12.5%] participants).

Similar benefits were reported in OS and PFS and the safety profile of T-DXd in the study population was generally manageable and tolerable.

In China, the standard of care (SoC) in the third-line setting is limited to treatment with individual chemotherapy agents, nivolumab, or apatnib. As the efficacy of these agents is modest (ORR [redacted] median PFS [redacted], and median OS [redacted] and unfavorable compared with T-DXd, a comparator arm is not considered appropriate for this study (Xuan 2014, Li et al 2016, Chen et al 2020). Thus, while the design of the current study is very similar to that of Study DESTINY-Gastric01, this will be a single-arm study and all eligible participants will receive T-DXd monotherapy.

Results from Study [redacted] also showed T-DXd has benefit in [redacted] population, confirmed ORR by ICR (FAS) in [redacted] comparable to that in [redacted] population [redacted] [95% CI: [redacted] These data suggest that the antitumor activity of T-DXd may also extend to [redacted] population and this warrants further evaluation. The similar efficacy results in HER2 IHC 2+ population were seen in another anti-HER2 ADC, RC-48. The ORR of RC-48 was 24.8% (95% CI: 17.5%-33.3%), and showed almost similar ORR (FAS) in IHC 2+ (23.0% [95% CI: 13.2, 35.5]) and IHC 3+ population (26.6% [95% CI: 16.3, 36.1]).

So there is rationale to evaluate the efficacy and safety of T-DXd in the expanded population (HER2-expressing: IHC 3+ or IHC 2+) in this study.

4.2.2 Rationale for T-DXd in HER2-expressing Gastric and GEJ Adenocarcinoma

Nonclinical pharmacology data have demonstrated the potent HER2-targeting antitumour

activity of T-DXd through the released drug, MAAA-1181a, inducing DNA damage and apoptosis by topoisomerase I inhibition. T-DXd also exhibits antibody-dependent cellular cytotoxic activity and HER2-mediated Akt phosphorylation inhibition. In vitro studies indicate that T-DXd specifically binds to HER2 and exhibits HER2-expression-dependent cell growth inhibitory activity. In vivo studies indicate that the HER2-targeting antitumour activity of T-DXd is more potent than that of the anti-HER2 antibody, MAAL-9001 and of T-DM1.

In China, HER2-expressing (IHC 3+ or 2+) rate is reported in 22% to 24% of GC patients (Fan et al 2013, Shan et al 2013, Sheng et al 2013) and, as discussed in Section 2.1, HER2 is considered an important target protein for GC therapy. The rationale for inclusion of participants in China with HER2-expressing gastric and GEJ adenocarcinoma reflects the positive efficacy and safety outcomes reported in Study DESTINY-Gastric01 and Study DESTINY-Gastric01 exploratory cohort 1 in which participants with HER2-expressing (IHC 3+ or IHC 2+) advanced gastric or GEJ adenocarcinoma were treated with T-DXd 6.4 mg/kg every 3 weeks.

4.2.3 Rationale for Study Endpoints

The primary endpoint is:

- Confirmed ORR, defined as the proportion of participants who have a confirmed CR or confirmed PR, as determined by ICR per RECIST 1.1.

Confirmed ORR by ICR represents the percentage of participants whose disease decreases in size or disappears and provides an early signal for clinical benefit. It is an appropriate and reasonable efficacy endpoint for single-arm studies performed in China provided, in accordance with the requirements of NMPA’s Center for Drug Evaluation, efficacy response assessment is performed by independent review.

Additional secondary efficacy endpoints (ORR, DCR, DoR, PFS, and tumour size change by investigator assessment, and OS) will be used to further evaluate efficacy and to corroborate the benefits of antitumor effects demonstrated by ORR.

As part of the secondary endpoint assessment, blood samples will be taken to allow for research into the PK and immunogenicity of T-DXd in Chinese participants.

4.3 Justification for Dose of T-DXd

The recommended dosage and administration of T-DXd for patients with locally advanced or metastatic HER2-positive GC or GEJ adenocarcinoma is 6.4 mg/kg every 3 weeks, as established based on efficacy and safety results from Studies DS8201-A-J101 and DESTINY-Gastric01, comparison of dose-exposure relationship between participants with breast cancer in Study DS8201-A-J101 and participants with GC in Study DESTINY-Gastric01, and

exposure-response (ER) analyses for both efficacy and safety.

Following administration of T-DXd every 3 weeks at 5.4 mg/kg or 6.4 mg/kg to participants with HER2-positive GC enrolled in Part 2b of Study DS8201-A-J101, the 6.4 mg/kg group showed a higher ORR ([redacted]) than the 5.4 mg/kg ([redacted]), and the response in the 6.4 mg/kg group was durable. Though the incidence and severity of AEs tended to be higher in the 6.4 mg/kg group than the 5.4 mg/kg group, the AEs were tolerable and generally manageable in both treatment groups. A comparison of the exposure of T-DXd in participants with GC given a 6.4 mg/kg dose was [redacted] lower than the exposure of T-DXd in participants with breast cancer who were given a 6.4 mg/kg dose. Based on the tolerability, safety, efficacy, and PK profile in Study DS8201-A-J101, 6.4 mg/kg every 3 weeks was selected as the dose regimen for Study DESTINY-Gastric01.

In Study DESTINY-Gastric01, the 6.4 mg/kg dose showed clinically meaningful efficacy with respect to ORR and OS. The ORR in the T-DXd 6.4 mg/kg group was statistically significantly higher (40.5% [95% CI: 31.8, 49.6]) than that in the physician's choice group (11.3% [95% CI: 4.7, 21.9]) (Cochran–Mantel–Haenszel test, $p < 0.0001$). There was a statistically significant and clinically meaningful prolongation of OS in the T-DXd arm compared with the physician's choice group (stratified log-rank test, $p = 0.0097$). The safety in participants with GC in the 6.4 mg/kg arm was acceptable compared with the physician's choice group, and was manageable.

The population PK (popPK) analysis showed that the exposure of T-DXd in participants with GC given a 6.4 mg/kg dose was [redacted] to that in participants with [redacted] given the approved [redacted] dose.

The ER analysis for efficacy showed that after administration of T-DXd at 6.4 mg/kg, participants with higher exposure of T-DXd tended to exhibit higher efficacy, indicating added benefit from using the higher 6.4 mg/kg dose.

The ER analysis for safety showed that the incidence of ILD tended to be higher in participants with higher exposure of T-DXd. The incidence of ILD at 6.4 mg/kg in participants with GC was estimated to be [redacted] to that after administration of [redacted] to participants with [redacted], [redacted] of T-DXd and DXd at 6.4 mg/kg in GC compared with [redacted] mg/kg in [redacted] There was a clear trend for higher rates of AEs in participants with higher average serum concentration of DXd, with the notable exception of AEs associated with study drug discontinuation, [redacted] AEs with a higher incidence in participants with GC than those with [redacted] were mainly [redacted] and the incidence of these events increased with increasing exposure of DXd. However, these AEs were generally manageable without discontinuing treatment by dose modification and routine clinical practice.

Based on the benefit-risk analysis, the dosing regimen of T-DXd 6.4 mg/kg every 3 weeks is considered to be appropriate in participants with locally advanced or metastatic HER2-positive GC or GEJ adenocarcinoma who have received 2 or more prior regimens.

Refer to the IB for further information on the nonclinical and clinical data for T-DXd.

Information on dose modifications for T-DXd is provided in Section 6.6.

4.4 End of Study Definition

A participant is considered to have completed the study if he/she has completed all phases of the study including the last scheduled procedure shown in the SoA (Section 1.3), including follow-up for OS determination.

The study may be stopped if, in the judgment of AstraZeneca, study participants are placed at undue risk because of clinically significant findings. In addition, the study may be terminated at individual centres if study procedures are not being performed according to ICH GCP or if the recruitment rate does not allow for the study to be completed in the planned timeframe.

The end of the study is defined as the time of the final DCO for the full final analysis. Final analysis is planned to be performed approximately [redacted] months after the last participant initiates study intervention. Based on data from Study DESTINY-Gastric01, this will allow the last participant sufficient opportunity to achieve a treatment response (estimated at [redacted] months) and sufficient DoR follow-up for responders (estimated at [redacted] months).

See Section 6.7 for details on participant management following the final DCO as well as following study completion.

5 STUDY POPULATION

The target population of primary interest in this study is participants in China with HER2-positive (IHC 3+ or IHC 2+/ISH + by central laboratory) advanced gastric or GEJ adenocarcinoma who have received at least 2 prior regimens including a fluoropyrimidine agent and a platinum agent. The study will enrol participants who have HER2-expressing (defined as IHC 3+ or IHC 2+) status, in which efficacy and safety will be evaluated as secondary endpoints.

For the purposes of eligibility, HER2 status should be determined at site based on a documented pathology result obtained from either a local laboratory or from a previous pathology report that has been confirmed by the investigator. In addition, HER2 status will be confirmed by central laboratory. If the result of HER2 testing by the central laboratory indicates that the participant is not HER2-expressing (IHC 3+ or IHC 2+), the participant may continue in the study and continue to receive study intervention. Prospective approval of

protocol deviations to recruitment and enrolment criteria, also known as protocol waivers or exemptions, is not permitted.

Participants who do not meet the eligibility criteria requirements are screen failures (see Section 5.4).

5.1 Inclusion Criteria

Participants are eligible to be included in the study only if all of the following criteria apply:

Age

1. Male or female participant aged ≥ 18 years at the time of screening.

Type of Participant and Disease Characteristics

2. Pathologically documented locally advanced unresectable or metastatic gastric or GEJ adenocarcinoma.
3. Disease progression on or after ≥ 2 prior regimens for advanced/metastatic disease that included a fluoropyrimidine and a platinum:
   (a) Progression within 6 months of prior adjuvant or neoadjuvant chemotherapy will count as rapid progression in the neoadjuvant/adjuvant setting and is thus equivalent to advanced/metastatic disease failing 1 regimen of therapy
   (b) If prior combination therapy was discontinued due to an AE and then one of the combination agents was continued, this is considered to be 1 prior regimen, not 2 prior regimens
   (c) A change in dosage form of the fluoropyrimidine (eg, intravenous to oral administration) without progression is considered to be 1 prior regimen, not 2 prior regimens.
4. Eastern Cooperative Oncology Group (ECOG) performance status 0-1.
5. Documentation of HER2 IHC 3+ or IHC 2+, regardless of ISH status, during screening.

   For the purposes of study eligibility, HER2 expression status should be determined at site based on a documented pathology result obtained from either a local laboratory or from a previous pathology report that has been confirmed by the investigator. If HER2 status has not been documented previously, a newly-acquired tumour sample must be provided for local assessment of HER2 status prior to enrolment if the primary tumour is accessible by endoscopy or if metastatic tissue can be biopsied. If a newly-acquired sample is not available, an archived sample is acceptable (based on the most recent archived tumour tissue sample).
6. Willing and able to provide an adequate newly-acquired tumour sample for confirmation of HER2 status by a central laboratory specified by the sponsor if the primary tumour is accessible by endoscopy or if metastatic tissue can be biopsied. If a newly-acquired sample is not available, an archived sample is acceptable (based on the most recent

archived tumour tissue sample). See Section 8.6.1 and the Laboratory Manual for further information.

7. At least 1 lesion that can be accurately measured at baseline as ≥ 10 mm in the longest diameter (except lymph nodes, which must have short axis ≥ 15 mm) with CT or MRI and is suitable for accurate repeated measurements, as per RECIST 1.1.
8. Adequate organ and bone marrow function (as defined in Table 5) within 14 days prior to study enrolment. All parameters must meet the inclusion criteria and must be the most recent results available.

Table 5 Parameters for Adequate Organ and Bone Marrow Function

**Adequate bone marrow function**	
Platelet count	≥ 100000/mm^3<br>Platelet transfusion is not allowed within 7 days prior to the screening assessment
Haemoglobin	≥ 8.0 g/dL<br>Red blood cell transfusion is not allowed within 7 days prior to the screening assessment. Participants requiring ongoing transfusions or growth factor support to maintain haemoglobin ≥ 8.0 g/dL are not eligible
Absolute neutrophil count	≥ 1500/mm^3<br>Granulocyte-colony stimulating factor administration is not allowed within 7 days prior to screening assessment
**Adequate hepatic function**	
ALT and AST	≤ 3 × ULN (or < 5 × ULN in participants with liver metastases)
Total bilirubin	≤ 1.5 × ULN if no liver metastases or < 3 × ULN in the presence of documented Gilbert's syndrome (unconjugated hyperbilirubinaemia) or liver metastases at baseline
Serum albumin	≥ 2.5 g/dL
**Adequate renal function**	
CrCL	≥ 30 mL/min as determined by the Cockcroft Gault equation, using actual body weight:<br>Males:<br>$\text{CrCL (mL/min)} = \dfrac{\text{Weight (kg)} \times (140 - \text{Age})}{72 \times \text{serum creatinine (mg/dL)}}$<br>Females:<br>$\text{CrCL (mL/min)} = \dfrac{\text{Weight (kg)} \times (140 - \text{Age})}{72 \times \text{serum creatinine (mg/dL)}} \times 0.85$
**Adequate blood clotting function**	
INR or PT and either PTT or aPTT	≤ 1.5 × ULN

ALT = alanine aminotransferase; aPTT = activated partial thromboplastin time; AST = aspartate aminotransferase; CrCL = calculated creatinine clearance; INR = international normalised ratio; PT = prothrombin time; PTT = partial thromboplastin time; ULN = upper limit of normal.

9. Left ventricular ejection fraction (LVEF) ≥ 50% by echocardiogram (ECHO) or multigated acquisition (MUGA) scan 28 days prior to enrolment.
10. Minimum life expectancy of 12 weeks.
11. Adequate treatment washout period prior to initiation of study intervention, as defined in Table 6.

Table 6 Adequate Treatment Washout Period

Treatment	Washout Period
Major surgery	≥ 4 weeks
Palliative radiotherapy	≥ 4 weeks for palliative radiotherapy with wide field of radiation or to more than 30% of the bone marrow or radiation therapy including palliative stereotactic radiation therapy to chest<br>≥ 2 weeks for palliative radiotherapy with a limited field of radiation or for whole brain radiation
Anti-Cancer chemotherapy [Immunotherapy (non-antibody based therapy)], retinoid therapy, hormonal therapy	≥ 3 weeks
Targeted agents and small molecules	≥ 2 weeks or 5 half-lives whichever is longer
Nitrosureas and mitomycin C	≥ 6 weeks
Antibody-based anticancer therapy	≥ 4 weeks
Chloroquine and hydroxychloroquine	≥ 14 days
Cell-free and Concentrated Ascites Reinfusion Therapy (CART), peritoneal shunt or drainage of pleural effusion, ascites or pericardial effusion	≥2 weeks prior to screening assessment
Anticancer traditional Chinese medicines (ie, those with a label for the treatment of cancer)	≥ 14 days

Reproduction

Contraceptive use by men or women should be consistent with local regulations regarding the methods of contraception for those participating in clinical studies.

12. Negative serum pregnancy test at screening for women of childbearing potential (WOCBP) who are sexually active with a non-sterilised male partner.
13. Female participants must be 1 year post-menopausal, surgically sterile, or using one highly effective form of birth control (a highly effective method of contraception is defined as one that can achieve a failure rate of less than 1% per year when used consistently and correctly; see Table 18). For women who are on hormone replacement therapy (HRT),

please refer to Appendix F. WOCBP who are sexually active with a non-sterilised male partner must agree to use one highly effective method of birth control. They should have been stable on their chosen method of birth control for a minimum of 3 months before entering the study until 7 months after the last dose of study intervention (see Appendix F, Table 18 for complete list of highly effective birth control methods). Complete heterosexual abstinence for the duration of the study and study intervention washout period is an acceptable contraceptive method if it is line with the participant's usual lifestyle (consideration must be made to the duration of the clinical trial); however, periodic or occasional abstinence, the rhythm method, and the withdrawal method are not acceptable. Female participants must refrain from egg cell donation and breast-feeding while on the study and for 7 months after the last dose of study intervention. Preservation of ova may be considered prior to enrollment in this study.Non-sterilised male partners of a WOCBP must use a male condom plus spermicide (condom alone in countries where spermicides are not approved) throughout this period.

14. Male participants who intend to be sexually active with a female partner of childbearing potential must be surgically sterile or using an acceptable method of contraception (see Appendix F) from the time of screening throughout the total duration of the study and until 4 months after the last dose of study intervention to prevent pregnancy in a partner. Male participants must not donate or bank sperm during this same time period. Not engaging in heterosexual activity (sexual abstinence) for the duration of the study and for the study intervention washout period is an acceptable practice if this is the preferred usual lifestyle of the participant; however, periodic or occasional abstinence, the rhythm method, and the withdrawal method are not acceptable methods of contraception.

Informed Consent

15. Capable of giving signed informed consent as described in Appendix A which includes compliance with the requirements and restrictions listed in the ICF and in this protocol.

5.2 Exclusion Criteria

Participants are excluded from the study if any of the following criteria apply:

Medical Conditions

1. As judged by the investigator, any evidence of diseases (such as severe or uncontrolled systemic diseases, including ongoing or active infection, uncontrolled hypertension, renal transplant and active bleeding diseases, serious chronic GI conditions associated with diarrhoea, which, in the investigator's opinion, makes it undesirable for the participant to participate in the study or that would jeopardise compliance with the protocol.

2. A pleural effusion, ascites or pericardial effusion that requires drainage, peritoneal shunt, or Cell-free and Concentrated Ascites Reinfusion Therapy (CART). Drainage and CART are not allowed within 2 weeks prior to screening assessment.
3. History of another primary malignancy except for malignancy treated with curative intent with no known active disease within 3 years before the first dose of study intervention and of low potential risk for recurrence. Exceptions include basal cell carcinoma of the skin and squamous cell carcinoma of the skin that has undergone potentially curative therapy, adequately resected non-melanoma skin cancer, curatively treated in situ disease, or other solid tumours curatively treated.
4. Has unresolved toxicities from previous anticancer therapy, defined as toxicities (other than alopecia) not yet resolved to Grade ≤ 1 or baseline. Note: Subjects may be enrolled with chronic, stable Grade 2 toxicities (defined as no worsening to >Grade 2 for at least 3 months prior to [randomization/enrollment/Cycle 1 Day 1] and managed with standard of care treatment) that the investigator deems related to previous anticancer therapy, such as:
   - Chemotherapy-induced neuropathy
   - Fatigue
   - Residual toxicities from prior IO treatment: Grade 1 or Grade 2 endocrinopathies which may include:
     a) Hypothyroidism/hyperthyroidism
     b) Type 1 diabetes
     c) Hyperglycaemia
     d) Adrenal insufficiency
     e) Adrenalitis
     f) Skin hypopigmentation (vitiligo)

5. Has spinal cord compression or clinically active central nervous system metastases, defined as untreated and symptomatic, or requiring therapy with corticosteroids or anticonvulsants to control associated symptoms. Subjects with clinically inactive brain metastases may be included in the study. Subjects with treated brain metastases that are no longer symptomatic and who require no treatment with corticosteroids or anticonvulsants may be included in the study if they have recovered from the acute toxic effect of radiotherapy. A minimum of 2 weeks must have elapsed between the end of whole brain radiotherapy and study enrolment/randomization.
6. Active primary immunodeficiency, known uncontrolled human immunodeficiency virus (HIV) infection or active hepatitis B or C infection. , or active hepatitis B or C infection. Participants positive for hepatitis C virus (HCV) antibody are eligible only if polymerase chain reaction is negative for HCV RNA. Participants should be tested for HIV prior to enrolment if required by local regulations or institutional review board (IRB)/independent ethics committee (IEC).
7. Uncontrolled infection requiring IV antibiotics, antivirals, or antifungals.

8. Receipt of live, attenuated vaccine (mRNA and replication deficient adenoviral vaccines are not considered attenuated live vaccines) within 30 days prior to the first dose of study intervention. Note: Participants, if enrolled, should not receive live vaccine during the study and up to 30 days after the last dose of study intervention.
9. Has substance abuse or any other medical conditions such as clinically significant cardiac or psychological conditions, that may, in the opinion of the investigator, interfere with the participant's participation in the clinical study or evaluation of the clinical study results.
10. Participants with a medical history of myocardial infarction (MI) within 6 months before enrolment, symptomatic CHF (NYHA Class II to IV), unstable angina pectoris, or a recent (< 6 months) cardiovascular event including stroke. Participants with troponin levels above the upper limit of normal (ULN) at screening (as defined by the manufacturer), and without any myocardial-related symptoms, should have a cardiological consultation before enrolment to rule out MI.
11. Investigator judgement of one or more of the following:
    (a) Mean resting QT interval corrected by Fridericia's formula (QTcF) interval > 470 msec (females) or > 450 msec (males) obtained from triplicate 12-lead ECGs taken at screening, where

$$QTcF = \frac{QT}{\sqrt[3]{RR}}$$

    (b) History of QT prolongation associated with other medications that required discontinuation of that medication, or any current concomitant medication known to prolong the QT interval and cause Torsdes de Pointes
    (c) Congenital long QT syndrome, family history of long QT syndrome, or unexplained sudden death under 40 years of age in first-degree relatives.

12. Lung criteria:
    (a) History of (non-infectious) ILD/pneumonitis that required steroids, current ILD/pneumonitis, or where suspected ILD/pneumonitis cannot be ruled out by imaging at screening
    (b) Lung-specific intercurrent clinically significant illnesses including, but not limited to, any underlying pulmonary disorder (eg, pulmonary emboli within 3 months of study enrolment, severe asthma, severe chronic obstructive pulmonary disease, restrictive lung disease, pleural effusion etc)
    (c) Prior pneumonectomy(complete).

13. Any autoimmune, connective tissue or inflammatory disorders (eg, rheumatoid arthritis, Sjogren's, sarcoidosis, etc), where there is documented, or a suspicion of pulmonary

involvement at the time of screening. Full details of the disorder should be recorded in the electronic Case Report Form (eCRF) for participants who are included in the study.

Prior/Concomitant Therapy

14. Prior exposure, without adequate treatment washout prior to enrolment for therapies detailed in Table 6.
15. Any concurrent anticancer treatment, Concurrent use of hormonal therapy for non-cancer related conditions (eg, HRT) is allowed. Washout periods for prior therapies are provided in Table 6.
16. Major surgical procedure (excluding placement of vascular access) or significant traumatic injury within 4 weeks of the first dose of study intervention or an anticipated need for major surgery during the study.
17. Palliative radiotherapy with a limited field of radiation within 2 weeks or with wide field of radiation or to more than 30% of the bone marrow within 4 weeks before the first dose of study intervention. Radiation therapy, including palliative stereotactic radiation therapy to chest ≥ 4 weeks. See Table 6.

Prior/Concurrent Clinical Study Experience

18. Previous treatment in the present study.
19. Randomisation into a prior T-DXd study regardless of treatment assignment, or concurrent enrolment in another clinical study, unless it is an observational (non-interventional) clinical study or during the follow-up period of an interventional study.
20. Known allergy or hypersensitivity to study intervention or any of the study intervention excipients.
21. History of severe hypersensitivity reactions to other mAbs.

Other Exclusions

22. Involvement in the planning and/or conduct of the study (applies to both AstraZeneca staff and/or staff at the study site).
23. Judgment by the investigator that the participant should not participate in the study if the participant is unlikely to comply with study procedures, restrictions and requirements.
24. Currently pregnant (confirmed with positive pregnancy test) or breastfeeding, or who are planning to become pregnant.
25. Subjects with past or resolved hepatitis B virus (HBV) infection or inactive chronic HBV infection are eligible only if they meet the following criteria and such patients should be closely monitored for HBV reactivation.

i. HBsAg(-) (on or off anti-viral treatment), AND all of the following criteria:
   a. anti-HBc (+) (IgG or total Ig),
   b. HBV viral DNA < 2,000 IU/mL,
   c. ALT ≤ ULN,
   d. Liver architecture normal (absence of any liver pathology including cirrhosis, fibrosis, and liver metastases),
   e. Has access to a local Hepatitis B expert during and after the study,
   f. Absence of HCV co-infection or history of HCV co-infection,
   g. Prophylactic anti-viral treatment starting at a minimum of 7 days before commencing study drug(s) is required if HBV viral DNA is detectable at baseline.

ii. HBsAg(+) AND all of the following criteria:
   h. Anti-HBc (+),
   i. HBeAg(-),
   j. HBV viral DNA < 2,000 IU/mL,
   k. ALT ≤ ULN,
   l. Liver architecture normal (absence of any pathology including cirrhosis, fibrosis or liver metastases)
   m. No presence or family history of HCC or cirrhosis, no extrahepatic manifestations e.g. arthritis, polyarteritis nodosa,
   n. Has access to a local Hepatitis B expert during and after the study,
   o. Absence of HCV co-infection or history of HCV co-infection,
   p. Prophylactic anti-viral treatment starting at a minimum of 7 days before commencing study drug(s) is required.

5.3 Lifestyle Considerations

The following restrictions apply while the participant is receiving study intervention and for the specified times before and after:

- Participants must follow the contraception requirements outlined in Appendix F.
- Participants should not donate blood or blood components while participating in this study and through 40 (+ 7) days after the last dose of study intervention.
- Use of tobacco products, e-cigarettes and vaping is strongly discouraged but not prohibited. Participants should inform the investigator about any prior or current use of these products and details should be recorded in the eCRF.
- Concomitant use of dietary supplements, medications not prescribed by the investigator, and alternative/complementary treatments is discouraged, but not prohibited.
- Preservation of sperm should be considered prior to enrolment in this study.

Restrictions relating to concomitant therapies are described in Appendix G 1.

5.4 Screen Failures

Screen failures are defined as participants who consent to participate in the clinical study but are not subsequently assigned to treatment. A minimal set of screen failure information is required to ensure transparent reporting of screen failure participants to meet the Consolidated Standards of Reporting Trials publishing requirements and to respond to queries from regulatory authorities. Minimal information includes demography, screen failure details, eligibility criteria, and any serious adverse event (SAE).

These participants should have the reason for study withdrawal recorded in the (eCRF) as "eligibility criteria not fulfilled" (ie, participant does not meet the required inclusion/exclusion criteria). This reason for study withdrawal is only valid for screen failures. The date of screening failure is defined as the date the site determined that the participant was ineligible.

Individuals who do not meet the eligibility criteria for participation in this study (screen failures) may be rescreened once with respect to HER2 status and once with respect to other screening procedures (see Section 1.3.1).

- Participants who are rescreened are required to sign a new ICF.
- Rescreened participants should be assigned the same participant number (ie, E-code) as for the initial screening.
- Rescreening should be documented so that its effect on study results, if any, can be assessed.
- All assessments must be repeated for rescreening unless they are within 28 days of the participant being enrolled in the interactive response technology (IRT).

Participant enrolment is described in Section 6.3.

6 STUDY INTERVENTION

Study intervention is defined as any investigational intervention, marketed product, placebo, or medical device intended to be administered to a study participant according to the CSP.

6.1 Study Interventions Administered

6.1.1 Investigational Products

The investigational product in this study is T-DXd. Details of the study intervention are provided in Table 7. AstraZeneca will supply T-DXd.

All participants included in the study will receive T-DXd 6.4 mg/kg as an IV infusion once

every 3 weeks, on Day 1 of each 3-week cycle. Participants may continue to receive study intervention until RECIST 1.1-defined PD, unacceptable toxicity, withdrawal of consent, or until any other criterion for discontinuation of study intervention is met (see Section 7.1). Continuation of study intervention beyond RECIST 1.1-defined PD is not permitted in this study.

Participants may continue to receive T-DXd as long as they are continuing to show clinical benefit, as judged by the investigator and in the absence of discontinuation criteria.

Dose modifications are described in Section 6.6.

Table 7 Study Intervention: T-DXd

**Arm Name**	T-DXd arm
**Intervention Name**	T-DXd
**Type**	Drug
**Dose Presentation**	Vial
**Unit Dose Strength**	Powder for concentrate for solution for infusion 100 mg/vial
**Dosage Level**	6.4 mg/kg
**Route of Administration**	IV infusion
**Use**	Experimental
**IMP or NIMP**	IMP
**Sourcing**	Provided centrally by sponsor
**Packaging and Labelling**	Study intervention will be provided in 100 mg vials in cartons. Each vial and carton will be labelled in accordance with Good Manufacturing Practice Annex 13 and per country requirements [a]
**Former Name/Alias**	DS8201a

[a] Label text for T-DXd (DS8201a) will show “DS8201a” depending on the agreed product name used in the respective approved study master label document. All naming conventions for these compounds are correct during this transitional period.

IMP = investigational medicinal product; IV = intravenous; NIMP = non-investigational medicinal product; T-DXd = trastuzumab deruxtecan.

6.2 Preparation/Handling/Storage/Accountability of Interventions

1. The investigator or designee (eg, pharmacist) must confirm appropriate temperature conditions have been maintained during transit for all study intervention received and any discrepancies are reported and resolved before use of the study intervention.
2. Only participants enrolled in the study may receive study intervention and only authorised site staff may supply or administer study intervention. All study intervention must be stored in a secure, environmentally controlled, and monitored (manual or automated) area in accordance with the labelled storage conditions with access limited to the investigator and authorised site staff.

3. The investigator, institution, or the head of the medical institution (where applicable) is responsible for study intervention accountability, reconciliation, and record maintenance (ie, receipt, reconciliation, and final disposition records).
4. Further guidance and information for the final disposition of unused study interventions are provided in the Study Reference Manual or other specified location.

6.2.1 T-DXd Preparation, Administration and Storage

T-DXd will be supplied as a 100 mg/vial lyophilised powder for concentrate for solution for infusion. Following reconstitution with sterile water for injection, the solution contains 20 mg/mL T-DXd in 25 mM histidine/histidine hydrochloride, 90 mg/mL sucrose, 0.03% (w/v) polysorbate 80; it has a pH of 5.5. The post-reconstitution label-claim volume is 5 mL.

The reconstituted product is a clear to opalescent, colourless to yellow liquid, and practically free from visible particles.

Preparation of T-DXd

The dose of T-DXd for administration must be prepared by the investigator's or site's designated study intervention manager using aseptic technique. Total time from needle puncture of the T-DXd vial to the start of administration must not exceed:

- 24 hours at 2°C to 8°C (36°F to 46°F)
- 4 hours at room temperature

If the final product is stored at both refrigerated and ambient temperature, the total time must not exceed 24 hours.

Following preparation and during administration, the prepared IV bag must be covered by light protection cover.

Administration of T-DXd

It is recommended that participants receive prophylactic anti-emetic agents prior to infusion of T-DXd and on subsequent days. Anti-emetics such as 5-hydroxytryptamine 3 (5-HT_3) antagonists or neurokinin 1 (NK1) receptor antagonists and/or steroids (eg, dexamethasone) should be considered and administered in accordance with the prescribing information or institutional guidelines.

T-DXd will be administered using an IV bag containing 5% (w/v) Dextrose Injection infusion solution and delivered through an IV administration set with a 0.2- or 0.22-µm filter. The standard infusion time for T-DXd is approximately 90 (± 10) minutes for the first infusion. If the first infusion is well tolerated and the participant does not experience an IRR, the

minimum infusion time for subsequent cycles is 30 minutes. However, if there are interruptions during infusion, the total allowed time must not exceed 3 hours at room temperature.

Do not co-administer other drugs through the same infusion line.

The participant's weight at screening (baseline) will be used to calculate the initial dose of T-DXd. If, during the course of treatment, the participant's weight changes by ≥ ± 10%, the participant's dose will be recalculated based on the participant's updated weight. Thereafter, a dose recalculation will be required upon a ≥ ±10% change from the last updated weight.

Refer to the Pharmacy Instructions for detailed information about preparation and administration of T-DXd.

Monitoring of T-DXd Administration

Participants will be monitored during and after infusion of T-DXd. Vital signs will be measured according to the SoA (Table 2).

Management of study intervention-related toxicities are described in Appendix K. As with any biological product, allergic reactions to dose administration are possible. Therefore, appropriate drugs and medical equipment to treat acute anaphylactic reactions must be immediately available, and study personnel must be trained to recognise and treat anaphylaxis.

Storage of T-DXd

The investigator, or an appropriate delegate, will ensure that all study intervention is stored in a secured area, at appropriate temperatures and as specified on the label, and in accordance with applicable regulatory requirements.

- A calibrated temperature monitoring device will be used to record the temperature conditions in the drug storage facility.
- A temperature log will be used to record the temperature of the storage area.
- Temperature excursions outside the permissible range listed in the clinical supply packaging are to be reported to the Study Monitor upon detection.
- T-DXd vials are stored at 2°C to 8°C (36°F to 46°F) and must not be frozen. T-DXd must be kept in the original packaging until use to prevent prolonged light exposure.

6.3 Measures to Minimise Bias

Participant Enrolment

All participants will be centrally assigned to open-label study intervention using an IRT. Before the study is initiated, directions for the IRT will be provided to each site. The IRT will

provide the kit identification number to be allocated to the participant at each dispensing visit.

Investigators should keep a record (ie, the participant screening log) of participants who entered screening. At screening/baseline (Days -28 to -1), the investigators or suitably trained delegate will:

- Obtain signed informed consent before any study-specific procedures are performed. If laboratory or imaging procedures were performed for alternate reasons prior to signing consent, these can be used for screening purposes with consent of the participant. All screening laboratory and imaging results must have been obtained within 28 days prior to the participant being enrolled in the study.
- Participants will be identified to the IRT per country regulations. Obtain a unique 7-digit enrolment number (E-code), through the IRT in the following format (ECCNNXXX: CC being the country code, NN being the centre number, and XXX being the participant enrolment code at the centre). This number is the participant's unique identifier and is used to identify the participant on the eCRFs.
- Determine participant eligibility (see Sections 5.1 and 5.2).
- Following completion of all screening procedures and documentation of all baseline assessments, enrol eligible participant into the study and obtain a treatment assignment number. The treatment assignment number should be obtained from the IRT on the same day the participant's eligibility is confirmed and they are enrolled in the study. The date of enrolment is defined as the date the participant is confirmed as eligible in the IRT.

If the participant is ineligible for inclusion in the study for any reason, the IRT should be accessed to terminate the participant in the system. The date of screening failure is defined as the date the site determined that the participant was ineligible.

Refer to Section 5.4 for details of rescreening procedures, where applicable.

Participants will begin study intervention on Day 1; every effort should be made to ensure study intervention is initiated within 3 days of enrolment. Participants must not be treated unless all eligibility criteria have been met. Clinical status should be checked prior to the first dose of study intervention.

Study intervention will be dispensed at the study visits summarised in SoA (Table 2).

If a participant withdraws from the study, his/her enrolment code cannot be reused. Withdrawn participants will not be replaced.

Returned study intervention should not be re-dispensed to the participants.

Methods for Assigning Treatment Groups

Not applicable. This is an open-label, single arm study and all participants will receive the same study intervention. Potential bias in data interpretation will be reduced through the following:

- Efficacy: The primary endpoint, confirmed ORR, will be based on ICR.
- Safety: An independent ILD Adjudication Committee will review and adjudicate on all cases of potential ILD/pneumonitis.

Procedures for Handling Incorrectly Enrolled Participants

Participants who fail to meet the eligibility criteria should not, under any circumstances, be enrolled or receive study medication. There can be no exceptions to this rule. Participants who are enrolled but subsequently found not to meet all the eligibility criteria must not be started on study intervention and must be withdrawn from the study.

Where a participant does not meet all the eligibility criteria but is incorrectly started on study intervention, the investigator should inform the AstraZeneca study physician immediately, and a discussion should occur between the AstraZeneca study physician and the investigator regarding whether to continue or discontinue the participant from treatment. The AstraZeneca study physician must ensure all decisions are appropriately documented and that the potential benefit/risk profile remains positive for the participant.

6.4 Study Intervention Compliance

When participants are dosed at the site, they will receive study intervention directly from the investigator or designee, under medical supervision. The date, and time if applicable, of dose administered in the clinic will be recorded in the source documents and recorded in the eCRF. The dose of study intervention and study participant identification will be confirmed at the time of dosing by a member of the study site staff other than the person administering the study intervention.

Deviations from the prescribed dosage regimen should be recorded in the eCRF.

The on-site Study Pharmacist is responsible for managing the study intervention from receipt by the study site until the destruction or return of all unused study intervention.

6.5 Concomitant Therapy

Any concomitant treatment, procedure, or other medication considered necessary by the investigator for the participant’s safety and wellbeing (including over-the-counter or prescription medicines, vitamins, and/or herbal supplements) that the participant has received from the time of screening or receives during the study, including the 40-day (+ 7-day)

follow-up period following the last dose of study intervention, must be recorded in the eCRF along with:

- Reason for use.
- Dates of administration including start and end dates.
- Dosage information including dose and frequency.

The study physician should be contacted if there are any questions regarding concomitant or prior therapy.

If any concomitant therapy is administered due to a new or unresolved AE, it should be recorded.

Participants must be instructed not to take any medications, including over-the-counter products, without first consulting with the investigator.

Restricted, prohibited, and permitted concomitant medications/therapies are described in more detail in Appendix G 1.

Drug-drug Interactions

There is no information to date on drug-drug interactions with T-DXd, either preclinically or in participants. There may be a hypothetical interaction between T-DXd and hydroxychloroquine and/or chloroquine and therefore concomitant treatment with hydroxychloroquine or chloroquine is not allowed during the study intervention.

6.5.1 Prohibited Concomitant Medications

T-DXd safety-specific restrictions are listed below (refer also to Appendix G 1):

- Participants must be instructed not to take any medications, including over-the-counter products, without first consulting with the investigator.
- Participants, if assigned treatment, should not receive live vaccine during the study and up to 30 days after the last dose of study intervention. Participants who have received live, attenuated vaccine within 30 days prior to the first dose of T-DXd will be excluded.
- The following medications are prohibited during the study. The sponsor must be notified if a participant receives any of these during the study
  - Any concurrent chemotherapy, anticancer study intervention or biological, radiotherapy (except palliative radiotherapy to areas other than chest, after consultation with the study physician) or hormonal therapy for cancer treatment, including anticancer traditional Chinese medicines (ie, those with a label for cancer

treatment). Concurrent use of hormones for noncancer-related conditions (eg, insulin for diabetes and HRT) is acceptable

- T-DXd cannot be administered when the participant is taking immunosuppressive medications, including corticosteroids with the following exceptions:
  - short-term courses (< 2 weeks) of low to moderate dose (< 10 mg prednisolone per day or equivalent)
  - long-term, alternate-day treatment with short-acting preparations
  - maintenance physiological doses (replacement therapy)
  - administered topically (skin or eyes), by aerosol, or by intra-articular, bursal, or tendon injection
  - treatment with corticosteroids to prevent or treat hypersensitivity reactions to radiographic contrast agents is allowed. A temporary period of steroid treatment will be allowed for different indications after discussion with the study physician (eg, chronic obstructive pulmonary disorder [COPD], radiation, nausea, etc)
  - participants with bronchopulmonary disorders may use bronchodilators if only administered intermittently
  - use of immunosuppressive medications for the management of study intervention-related AEs or in participants with contrast allergies is acceptable.
- Immunosuppressive medications also include drugs like methotrexate, azathioprine, and tumour necrosis factor-alpha blockers.

- Concomitant treatment with chloroquine or hydroxychloroquine is not allowed during the study intervention. If treatment with chloroquine or hydroxychloroquine is absolutely required for COVID-19, study intervention must be interrupted. If chloroquine or hydroxychloroquine is administered, a washout period of at least 14 days is required before restarting study intervention.

6.5.2 Other Protocol Restrictions or Supportive Treatments

Other CSP-mandated restrictions or supportive treatments are listed below (see also Appendix G 1):

- Based on the currently available clinical safety data, it is recommended that participants receive prophylactic anti-emetic agents prior to infusion of T-DXd and on subsequent days. Anti-emetics such as 5-HT_3 antagonists or NK1 receptor antagonists and/or steroids (eg, dexamethasone) should be considered and administered in accordance with the prescribing information or institutional guidelines.
- Concomitant use of dietary supplements, medications not prescribed by the investigator, and alternative/complementary treatments is discouraged, but not prohibited.

- Prophylactic or supportive treatment of study intervention-induced AEs will otherwise be as per investigator's discretion and institutional guidelines.

Permitted/Rescue Therapies:

- Based on the currently available clinical safety data, it is recommended that patients receive prophylactic anti-emetic agents prior to infusion of T-DXd and on subsequent days.
- Use of immunosuppressive medications for the management of IMP-related AEs or in patients with contrast allergies is acceptable.
- Patients with Hepatitis B infection eligible for study participation should commence anti-viral therapy (if not already ongoing) prior to or at the time of starting steroids equivalent to > 20 mg prednisolone and likely to continue for ≥ 4 weeks. Anti-viral therapy must be managed in consultation with a local Hepatitis B expert.

6.6 Dose Modification

In case a dose reduction is necessary, the study intervention will be administered in accordance with Table 8 and the guidance in Table 23 (Appendix K).

Table 8 Dose Reduction Levels of T-DXd

Starting Dose	Dose Level -1	Dose Level -2
6.4 mg/kg	5.4 mg/kg	4.4 mg/kg

Once the dose of T-DXd has been reduced because of toxicity, all subsequent cycles should be administered at that lower dose level unless further dose reduction is required (Table 8). More than 2 dose reductions are not allowed, and the study intervention will be discontinued if further toxicity that meets the requirement for dose reduction occurs.

Dose delays are permitted for T-DXd treatment and the dosing interval for the next T-DXd cycle may be shortened, as clinically feasible, to gradually align with the schedule of tumour efficacy assessment. Two consecutive doses must be administered at least [redacted] apart. Every effort should be made to limit study drug delay, however, in circumstances, such as of adverse event management or medical intervention, the study drug can be held up to [redacted] ([redacted]) from the last T-DXd dose. During this time scheduled CT/MRI scans should continue as per protocol, and patients should fulfil all of the following criteria:

- Study drug may be resumed with confirmation of continued benefit per RECIST 1.1. Scans should be performed at the frequency defined per protocol, while the drug is being held

    - At minimum 1 restaging scan must be done within 6 weeks prior to restarting the study drug
- IP(s) is/are restarted within the guidance of the TMGs for T-DXd and any combination agents, if appropriate
- No prohibited concomitant medications have been administered since the last dose of T-DXd

Treatment cycles for a participant for whom T-DXd dosing is temporarily withheld for any reason may have future cycles scheduled based on the date of the last T-DXd dose.

In addition, investigators may consider dose reductions or discontinuations of T-DXd according to the participant's condition and after discussion with the study physician or designee. For management of dose delays due to T-DXd related events, the TMGs (Appendix K) should be followed, as applicable.

In summary, if a participant experiences a clinically significant and/or unacceptable toxicity, dosing will be interrupted or permanently discontinued depending on the severity of the toxicity, and supportive therapy administered as required.

On improvement of an AE for which T-DXd was temporarily interrupted, T-DXd may be restarted at the same dose at the discretion of the investigator. If a further episode of the same AE subsequently requires dose interruption, or if a different AE subsequently requires dose interruption, T-DXd may be restarted at a one dose level reduction on improvement of the AE or discontinued if the participant is receiving the lowest protocol-specified dose level (Appendix K).

Appropriate and optimal treatment of the toxicity is assumed prior to considering dose modifications. Prior to discontinuation of study intervention due to toxicities, please consult with the sponsor study physician.

All dose modifications (interruption, re-initiation, reduction and/or discontinuation) should be based on the worst preceding toxicity (National Cancer Institute Common Terminology Criteria for Adverse Events [NCI CTCAE v5.0]). Specific criteria for interruption, re-initiation, dose reduction and/or discontinuation of T-DXd are listed in Appendix K, which is applicable only to treatment-emergent adverse events (TEAEs) that are assessed as related to use of T-DXd by the investigator(s). For non-drug related TEAEs, follow standard clinical practice. Appropriate clinical experts should be consulted as deemed necessary.

All confirmed or suspected COVID-19 infection events must be recorded in the eCRF. Refer to Appendix H for additional information on dose modification for suspected or confirmed COVID-19 infection for participants treated with T-DXd.

ILD/Pneumonitis Management Guidance

Please refer to the Guidance for Management of Participants with Drug-induced ILD/Pneumonitis summary flow chart in Appendix J for information on the management of participants with drug-induced ILD/pneumonitis. All potential ILD/pneumonitis cases should be reported within 24 hours; including both serious and non-serious potential ILD/pneumonitis cases (potential ILD/pneumonitis is defined in the current Site Manual List of Common Terminology Criteria for Adverse Events [MedDRA] Preferred Terms).

ILD/pneumonitis should be ruled out if a participant develops an acute onset of new or worsening pulmonary or other related signs/symptoms such as dyspnoea, cough, or fever. If the AE is confirmed to have an aetiology other than ILD/pneumonitis, follow the management guidance outlined in Appendix K.

If the AE is suspected to be ILD/pneumonitis, treatment with study intervention should be interrupted pending further evaluations. Evaluations should include those outlined in Section 8.2.5.2.1. As soon as ILD/pneumonitis is suspected, corticosteroid treatment should be started promptly as per clinical treatment guidelines.

If the AE is confirmed to be ILD/pneumonitis, follow the management guidance outlined in Appendix J and Appendix K. All events of ILD/pneumonitis regardless of severity or seriousness will be followed until resolution including after study intervention discontinuation.

To ensure adequate and relevant evaluation, systematic additional data collection will be conducted for all cases that will be brought for evaluation. This additional data collection will cover a more in-depth relevant medical history (eg, smoking, radiation, COPD, and other chronic lung conditions), diagnostic evaluation, treatment and outcome of the event. This data collection will be triggered for AEs reported using selected MedDRA Preferred Terms.

LVEF Decrease Management Guidance

- Left ventricular ejection fraction will be measured by either ECHO or MUGA scan. All ECHOs/MUGAs will be evaluated by the investigator or delegated physician for monitoring cardiac function.
- Troponin-T (preferably high-sensitivity troponin-T) will be measured locally at screening and at end of treatment, and as needed based on participant-reported cardiac signs or symptoms suggesting CHF, MI, or other causes of cardiac myocyte necrosis. If ECG is abnormal, follow institutional guidelines.
- Refer to Sections 8.2.3 (ECGs and troponin testing) and 8.2.5.1 (ECHO/MUGA acquisition scans) for details of investigations of cardiac function.

- Refer to Appendix K for information on the management of cardiac toxicities related to the study intervention.

Refer to Table 8 for protocol-specified dose reductions of T-DXd and to Appendix K for further information.

6.7 Intervention after the End of the Study

As described in Section 4.4, the study will remain open until all participants have discontinued study intervention and completed their last expected visit/contact.

Participants who continue to receive benefit from their assigned study intervention at the scheduled DCO for the final analysis and final database lock may continue to receive study intervention for as long as they and their physician feel they are gaining clinical benefit. Where participants continue to receive study intervention following the scheduled DCO for final analysis, it is recommended that participants continue the scheduled site visits (see Section 1.3).

For all study participants, investigators should continue to monitor and document data in the source notes after the scheduled DCO for the final analysis and final database lock. Depending on the results of the analysis, a decision may be made to continue further data collection for a longer period with the intent to analyse long-term OS and safety data to fulfil any other potential Health Authority requirements. Any additional long-term analysis may be further clarified through an addendum to the main Statistical Analysis Plan (SAP), which will be developed before the final DCO. Data will be collected until any of following conditions are met:

- Remaining participants in the study (including those who have discontinued study intervention) have discontinued the study, OR
- Remaining participants have been transferred into a roll-over study, OR
- If the sponsor decides to stop data collection, participants who are receiving study intervention at this time and deriving clinical benefit from their assigned study intervention will be allowed to continue treatment and only SAEs will be collected.

After the final DCO for this study, AstraZeneca will continue to supply T-DXd to participants who received T-DXd until meeting any discontinuation criteria as defined in Section 7.1. Where possible, if commercial supply of T-DXd is available in the local market, then this route should be used.

In the event that a roll-over or safety extension study is available at the time of the final DCO and database closure, participants currently receiving treatment with T-DXd may be transitioned to such a study, and the current study would reach its end. The roll-over or safety

extension study would ensure treatment continuation with visits and assessments per its protocol. Any participant who would be proposed to move to such a study would be asked to sign a new ICF. OS data collected in the roll-over study may be combined with OS data from this study and evaluated as a combined dataset.

7 DISCONTINUATION OF STUDY INTERVENTION AND PARTICIPANT DISCONTINUATION/WITHDRAWAL

7.1 Discontinuation of Study Intervention

It may be necessary for a participant to permanently discontinue (definitive discontinuation) study intervention. If study intervention is permanently discontinued, the participant will remain in the study to be evaluated for safety and survival follow-up. The investigator should instruct the participant to contact the site before or at the time if study intervention is stopped. A participant who decides to discontinue study intervention will always be asked about the reason(s) and the presence of any AEs. The reason for discontinuation should be documented in the source document and the appropriate section of the eCRF.

Participants who have permanently discontinued from further study intervention will need to be discontinued from the IRT.

Individual participants may be discontinued from study intervention in the following situations:

- RECIST 1.1-defined radiological progression as assessed by the investigator (refer to Section 8.1.1 and Appendix E).
- Investigator determination that the participant is no longer benefiting from study intervention.
- An AE that, in the opinion of the investigator or AstraZeneca, contraindicates further dosing.
- Any AE that meets criteria for discontinuation defined in the dose modification guidelines for management of study intervention-related toxicities (see Section 6.6).
- Participant decision. The participant is at any time free to discontinue treatment, without prejudice to further treatment. A participant who discontinues treatment is normally expected to continue to participate in the study (eg, for safety and survival follow-up) unless they specifically withdraw their consent to all further participation in any study procedures and assessments (see Section 7.2).
- Severe non-compliance with the CSP as judged by the investigator or AstraZeneca.
- Pregnancy or intent to become pregnant. Refer to Section 8.3.14 for information on pregnancy and to Appendix F for contraception requirements.
- Initiation of subsequent anticancer therapy, including another investigational agent.

Note that discontinuation from study intervention is NOT the same as withdrawal from the study.

Refer to the SoA for details of data to be collected at the time of study intervention discontinuation (ie, the end of treatment visit) and follow-up, and for any further evaluations that need to be completed.

7.1.1 Follow-up of Participants Post Discontinuation of Study Intervention

All participants who discontinue the study intervention will be followed up for safety assessments 40 (+ 7) days after their last dose of study intervention. Additional assessments to be performed at the time of the 40-day safety follow-up are detailed in the SoA (Table 3). For participants with ILD/pneumonitis and reactivation of Hepatitis B infection, safety follow-up will continue until the resolution of ILD/pneumonitis or reactivation of Hepatitis B infection.

Participants who have discontinued study intervention for any reason prior to objective progression per RECIST 1.1 as determined by the investigator, regardless of whether or not they have commenced subsequent anticancer therapy, will be followed up with tumour assessments as indicated in the SoA (Table 3) until RECIST 1.1-defined radiological PD or death regardless of whether or not the participant started a subsequent anticancer therapy, unless they have withdrawn all consent to study-related assessments.

7.1.2 Follow-up for Survival

Participants will be followed up for survival status as indicated in the SoA (Table 3) until death, withdrawal of consent, or the end of the study. Survival information may be obtained via telephone contact with the participant or the participant's family, or by contact with the participant's current physician. Additional assessments to be performed at the time of survival follow-up are detailed in the SoA (Table 3).

Note: Survival calls will be made following the date of DCO for the final analysis (these contacts should generally occur within 7 days of the DCO). If participants are confirmed to be alive or if the date of death is after the DCO date, these participants will be censored at the date of DCO.

7.2 Participant Withdrawal from the Study

- A participant may withdraw from the study at any time at his/her own request, or may be withdrawn at any time at the discretion of the investigator for safety, behavioural, compliance, or administrative reasons. This is expected to be uncommon.
- A participant who considers withdrawing from the study must be informed by the investigator about modified follow-up options to ensure the collection of endpoints and safety information including new AEs and any ongoing AEs and concomitant medications

(eg, telephone contact at 40 [+ 7] days after discontinuation of study intervention, a contact with a relative or treating physician, or information from medical records).

- At the time of withdrawal from the study, if possible, an Early Study Intervention Discontinuation visit should be conducted, as shown in the SoA (Table 2). See SoA for data to be collected at the time of study withdrawal and follow-up (Table 3) and for any further evaluations that need to be completed.
  - The participant will discontinue the study intervention and be withdrawn from the study at that time.
- If the participant withdraws consent for disclosure of future information, the sponsor may retain and continue to use any data collected before such a withdrawal of consent.
- If a participant withdraws from the study, it should be confirmed if he/she still agrees for existing samples to be used in line with the original consent. If the participant requests withdrawal of consent for use of samples, destruction of any samples taken and not tested should be carried out in line with what was stated in the informed consent and local regulation. The investigator must document the decision on use of existing samples in the site study records and inform the Global Study Team.

7.3 Lost to Follow-up

A participant will be considered lost to follow-up if he or she repeatedly fails to return for scheduled visits and no contact has been established by the time the study is completed (see Section 4.4), such that there is insufficient information to determine the participant's status at that time.

Participants who decline to continue participation in the study, including telephone contact, should be documented as "withdrawal of consent" rather than "lost to follow-up." Investigators should document attempts to re-establish contact with missing participants throughout the study period. If contact with a missing participant is re-established, the participant should not be considered lost to follow-up and evaluations should resume according to the protocol.

The following actions must be taken if a participant fails to return to the clinic for a required study visit:

- The site must attempt to contact the participant and reschedule the missed visit as soon as possible and counsel the participant on the importance of maintaining the assigned visit schedule and ascertain whether or not the participant wishes to and/or should continue in the study.
- Before a participant is deemed lost to follow-up, the investigator or designee must make every effort to regain contact with the participant (where possible, 3 telephone calls and,

if necessary, a certified letter to the participant's last known mailing address or local equivalent methods). These contact attempts should be documented in the participant's medical record.

- Should the participant continue to be unreachable, he/she will be considered to have been lost to follow-up from the study.
- Site personnel, or an independent third party, will attempt to collect the vital status of the participant during survival follow-up within legal and ethical boundaries for all participants, including those who did not get study intervention. Public sources may be searched for vital status information. If vital status is determined as deceased, this will be documented and the participant will not be considered lost to follow-up. Sponsor personnel will not be involved in any attempts to collect vital status information.

Discontinuation of specific sites or of the study as a whole are handled as part of Appendix A.

In order to support efficacy endpoints of PFS and OS, the survival status of all participants in the Full Analysis and the Safety Analysis Sets should be re-checked, including participants who withdrew consent or are classified as "lost to follow-up."

- Lost to follow-up – Site personnel should check hospital records and a publicly available death registry (if available), as well as checking with the participants' current physician, to obtain a current survival status.
- In the event that the participant has actively withdrawn consent to the processing of their personal data, the survival status of the participant can be obtained by site personnel from publicly available death registries (if available) where it is possible to do so under applicable local laws to obtain a current survival status.

8 STUDY ASSESSMENTS AND PROCEDURES

Study procedures and their timing (including permitted visit windows) are summarised in the SoA (Section 1.3). Data collection following study analysis until the end of the study is described below.

- Protocol waivers or exemptions are not allowed.
- The investigator is responsible for ensuring the accuracy, completeness, and timeliness of data recorded on the eCRFs and for the provision of responses to data queries. The investigator will sign the completed eCRFs and a copy of the completed eCRFs will be archived at site.

- Immediate safety concerns should be discussed with the sponsor immediately upon occurrence or awareness to determine if the participant should continue or discontinue study intervention.
- Adherence to the study design requirements, including those specified in the SoA (Section 1.3), is essential and required for study conduct.
- All screening evaluations must be completed and reviewed to confirm that potential participants meet all eligibility criteria. The investigator will maintain a screening log to record details of all participants screened and to confirm eligibility or record reasons for screening failure, as applicable.
- Procedures conducted as part of the participant’s routine clinical management (eg, blood count) and obtained before signing of the ICF may be utilised for screening or baseline purposes provided the procedures met the protocol-specified criteria and were performed within the time frame defined in the SoA.
- If a participant has an unscheduled assessment or visit, eg, as a result of an AE, all relevant data must be collected on the eCRF.
- If a participant undergoes unscheduled imaging, eg, to investigate clinical signs/symptoms of progression and is found not to have progressed, every attempt should be made to perform the subsequent imaging at the next regularly scheduled visit.

Data Collection Following Study Analysis until the End of the Study

Following the DCO for the final analysis, all participants who remain in the study will continue the scheduled survival follow-up site visits indicated in the SoA (Table 3). Refer to Section 6.7 for further details. In addition, any AEs of ILD/pneumonitis will be followed-up until resolution. For other safety reporting requirements after final analysis, see Section 8.3.12.

8.1 Efficacy Assessments

8.1.1 Imaging Tumour Assessments

Tumour assessments use images from CT (preferred) or MRI, with IV contrast, of the chest, abdomen, and pelvis (including the entire liver and both adrenal glands), collected during screening/baseline and at regular (follow-up) intervals (every 6 weeks [± 7 days]) during study intervention. Any other areas of disease involvement should also be imaged at screening based on known metastasis sites or signs and symptoms of the individual participant. Scans (CT or MRI) of the chest, abdomen, and pelvis are mandatory. Brain CT or MRI is required only in cases of pre-existing brain metastases or symptoms. The imaging modality used for baseline tumour assessment should be kept the same consistently at each subsequent follow-up assessment throughout the study, if possible. It is important to follow the tumour assessment schedule as closely as possible (refer to the SoA, Section 1.3) relative to first dose.

Screening/baseline imaging should be performed no more than 28 days before enrolment (confirmation of participant eligibility) and ideally should be performed as close as possible to and prior to the start of study intervention. Treatment continues until RECIST 1.1-defined PD, unacceptable toxicity, withdrawal of consent, or any other criterion for discontinuation of study intervention is met. Scanning/tumour assessments continue throughout treatment until RECIST 1.1-defined radiological progression by investigator's assessment. If an unscheduled assessment is performed (eg, to investigate clinical signs/symptoms of progression) and the participant has not progressed, every attempt should be made to perform the subsequent assessments at the next scheduled visit.

The RECIST 1.1 assessments of baseline images identify target lesions (TLs; defined as measurable) and non-target lesions (NTLs). On-study images are evaluated for TLs and NTLs chosen at baseline, and for new lesions (NLs) when they appear. This allows determination of follow-up TL response, NTL lesion response, the presence of unequivocal NLs, and overall time point responses (CR, PR, SD, PD, or not evaluable [NE]).

8.1.2 Central Reading of Scans

Images, including unscheduled visit scans, will be collected on an ongoing basis and sent to an AstraZeneca-appointed imaging Contract Research Organisation (iCRO) for quality control, storage, and for ICR. Digital copies of all original scans should be stored at the investigator site as source documents. Electronic image transfer from the sites to the iCRO is strongly encouraged. An ICR of images will be performed at the discretion of AstraZeneca. Results of these independent reviews will not be communicated to investigators, and results of investigator tumour assessments will not be shared with the central reviewers.

Further details of the ICR will be documented in an Independent Review Charter.

8.1.3 Overall Survival

Assessments for survival will be conducted every 3 months ± 14 days following objective PD or discontinuation of study intervention. Survival information may be obtained via telephone contact with the participant, participant's family, by contact with the participant's current physician, or local death registries as described in Sections 7.1.2 and 7.3.

8.1.4 Clinical Outcome Assessments

Not applicable.

8.2 Safety Assessments

Planned time points for all safety assessments are provided in the SoA (Section 1.3).

8.2.1 Physical Examinations

A complete or targeted physical examination will be completed as indicated in the SoA (Section 1.3).

- Complete physical examination will include assessments of the following; general appearance, respiratory, cardiovascular, abdomen, skin, head and neck (including ears, eyes, nose and throat), lymph nodes, thyroid, musculoskeletal (including spine and extremities), urogenital, dermatological, GI, endocrine, haematological/lymphatic, and neurological systems.
- Targeted physical examinations are to be used by the investigator on the basis of clinical observations and symptomatology.

Physical examination, as well as assessment of height (screening only) and weight, will be performed at timelines as specified in the SoA; investigators should pay special attention to clinical signs related to previous serious illnesses, and to new or worsening abnormalities that may qualify as AEs. Refer to Section 8.3.5 for details.

8.2.2 Vital Signs

The following vital signs will be evaluated at the time points indicated in the SoA (Section 1.3).

- Body temperature
- Pulse rate
- Respiratory rate
- Systolic and diastolic blood pressure.

Pulse oximetry (SpO_2) is described in Section 8.2.5.2.

Blood pressure and pulse measurements will be assessed with the participant in the seated or supine position using a completely automated device. Manual techniques will be used only if an automated device is not available.

Blood pressure and pulse measurements should be preceded by at least 5 minutes of rest for the participant in a quiet setting without distractions (eg, television, cell phones).

Vital signs (to be taken before blood collection for laboratory tests) will consist of 1 pulse and 3 blood pressure measurements (3 consecutive blood pressure readings will be recorded at intervals of at least 1 minute). The average of the 3 blood pressure readings will be recorded on the eCRF.

All measurements scheduled to be taken before infusion on Day 1 of a treatment cycle may be taken up to 3 days prior to administration of study intervention.

Situations in which vital signs results should be reported as AEs are described in Section 8.3.5.

In the event of an AE of IRR, the corresponding vital signs values should be entered into the eCRF.

8.2.3 Electrocardiograms

Triplicate ECGs will be performed at screening and before infusion on C1D1. Subsequent ECGs will be performed in triplicate in close succession only if abnormalities are noted. Single 12-lead ECGs will be performed at the times specified in the SoA after the participant has been resting semi-supine for at least 5 minutes and recorded while the participant remains in that position using an ECG machine that automatically calculates heart rate and measures PR, RR, QRS, QT, and QTcF intervals. Refer to the TMGs in Appendix K for management of prolonged average QTc > 500 ms or > 60 ms change from baseline.

All ECGs should be assessed by the investigator as to whether they are clinically significantly abnormal. Any clinically significant abnormalities detected require triplicate ECG results. At each time point at which triplicate ECG are required, 3 individual ECG tracings should be obtained in succession, no more than 2 minutes apart. Where required, the full set of triplicate ECGs should be completed within 5 minutes.

During treatment cycles, ECGs may be taken up to 3 days prior to administration of study intervention.

Troponin (preferably high-sensitivity troponin-T) will be collected at time points specified in the SoA (Section 1.3), including whenever a participant reports signs or symptoms suggesting CHF, MI, or other causes of myocyte necrosis:

- Participants with troponin levels above the ULN at screening (as defined by the manufacturer), and without any MI related symptoms, should have a cardiological consultation before enrolment to rule out MI.
- If troponin levels are above the ULN but below the level of MI defined by the manufacturer (CTCAE Grade 1), at baseline, no repeat testing is required.
- Only Grade 3 troponin needs to be repeated.
- If ECG is abnormal, follow institutional guidelines.

Situations in which ECG results should be reported as AEs are described in Section 8.3.5.

Whenever ECGs, vital signs, and blood draws are scheduled for the same nominal time, ECG assessments should occur first, then vital signs assessments, and then blood draws. The timing of the first 2 assessments should be such that it allows the blood draw (eg, PK blood sample) to occur at the timepoints indicated in the SoA.

8.2.4 Clinical Safety Laboratory Assessments

Blood and urine samples for determination of clinical chemistry, haematology, coagulation, and urinalysis will be taken at the visits indicated in the SoA (Section 1.3).

Additional safety samples may be collected if clinically indicated at the discretion of the investigator. The date, time of collection and results (values, units and reference ranges) will be recorded on the appropriate eCRF.

The analysis of clinical chemistry and haematology samples, and urinalysis will be performed at a local laboratory at or near the investigator site. Sample tubes and sample sizes may vary depending on laboratory method used and routine practice at the site.

Other safety laboratory tests include assessment for pregnancy and hepatitis B and C serology and HIV antibody test. Pregnancy tests may be performed at site using a licensed test, subject to the following:

- A negative serum pregnancy test (with a sensitivity of at least 25 mIU/mL) must be available at screening (within 72 hours prior to enrolment) for all female participants of childbearing potential
- A negative pregnancy test (serum or urine per institutional guidelines) must be available within 72 hours prior to each subsequent administration of study intervention and at the end of treatment visit for all female participants of childbearing potential.
- A positive urine pregnancy test result must immediately be confirmed using a serum test.

The laboratory variables indicated in Table 9 will be measured.

Table 9 Laboratory Safety Variables

Haematology/Haemostasis (Whole Blood)	Clinical Chemistry (Serum or Plasma)
Haemoglobin	Creatinine
Leukocyte count	Bilirubin, total
Leukocyte differential count (absolute count and percentage; neutrophils, lymphocytes, monocytes, eosinophils, basophils)	Alkaline phosphatase
Platelet count	AST
Total red blood cell count	ALT
Haematocrit	Albumin
**Urinalysis**	Potassium
Haemoglobin/Erythrocytes/Blood	Calcium, total
Protein/Albumin	Sodium
Glucose	Creatine kinase
**Coagulation**	Amylase
Coagulation variables (aPTT, PTT, and INR)	Gamma-glutamyl transferase
**Pregnancy Test**	Glucose (fasting)
Serum pregnancy test (with sensitivity of at least 25 mIU/mL) at screening; serum or urine thereafter	Lactate dehydrogenase
	Protein, total
	Blood urea nitrogen or Urea
	Troponin [a]
	Magnesium
	Chloride

[a] See Section 8.2.3 for details of requirements for troponin testing.
ALT = alanine aminotransferase; aPTT = activated partial thromboplastin time; AST = aspartate aminotransferase; INR = international normalised ratio; PTT = partial thromboplastin time.

Creatinine clearance should be determined according to the Cockcroft Gault equation, using actual body weight to confirm adequate renal function within 14 days prior to study enrolment.

Males:

$$\text{CrCL (mL/min)} = \frac{\text{Weight (kg)} \times (140 - \text{Age})}{72 \times \text{serum creatinine (mg/dL)}}$$

Females:

$$\text{CrCL (mL/min)} = \frac{\text{Weight (kg)} \times (140 - \text{Age}) \times 0.85}{72 \times \text{serum creatinine (mg/dL)}}$$

The investigator should assess the available results with regard to clinically relevant

abnormalities in documentation. Any clinically significant abnormal laboratory values should be repeated as clinically indicated (preferably within 24 to 48 hours) and recorded on the eCRF. Situations in which laboratory safety results should be reported as AEs are described in Section 8.3.5.

All participants with Grade 3 or 4 laboratory values at the time of completion or discontinuation from study intervention must be followed and have further tests performed until the laboratory values have returned to Grade 1 or 2, unless these values are not likely to improve because of the underlying disease.

NB. In case a participant shows an AST **or** ALT ≥ 3 × ULN together with total bilirubin (TBL) ≥ 2 × ULN, refer to Appendix D "Actions required in cases of increases in liver biochemistry and evaluation of Hy's Law" (HL), for further instructions.

8.2.5 Other Safety Assessments

8.2.5.1 Echocardiogram/Multigated Acquisition Scan

An ECHO or MUGA scan to assess LVEF will be performed at the visits shown in SoA (Section 1.3). The modality of the cardiac function assessments must be consistent for a given participant (ie, if ECHO is used at screening for a given participant, then ECHO should also be used for subsequent scans for that participant). The participants should also be examined using the same machine and operator whenever possible, and quantitative measurements should be taken (ie, accurate to 1% and not estimated to 5%). All ECHOs/MUGAs will be evaluated by the investigator or delegated physician for monitoring cardiac function.

If a participant has had an ECHO or MUGA performed within 4 weeks prior to treatment discontinuation, the discontinuation visit ECHO/MUGA scan is not required unless clinically indicated. If a participant has any clinically significant decrease in LVEF (greater than 10 percentage points to below 50%), there should be follow-up within 4 weeks until resolution.

Situations in which ECHO or MUGA results should be reported as AEs are described in Section 8.3.5.

8.2.5.2 Pulmonary Assessments

Pulse oximetry (SpO_2) should be evaluated by the investigator or the delegate physician at each study visit.

On Day 1 of each treatment cycle, SpO_2 should be evaluated by the investigator or the delegate physician prior to and at the end of the infusion of study intervention.

Pulmonary function tests should include basic (required) spirometry at a minimum, with optional additional components as detailed in Table 10.

Table 10 Spirometry Components

Required spirometry components	Optional spirometry components
FVC	Peak expiratory flow
FVC % predicted	DLCO
FEV1	FEV6
FEV1 % predicted	Total lung capacity
FEV1/FVC %	Residual volume

DLCO = diffusion capacity of the lungs for carbon monoxide; FEV = forced expiratory volume; FEV1 = FEV in 1 second; FEV6 = FEV in 6 seconds; FVC = forced vital capacity.

Diffusion capacity of the lungs (DLCO) will be performed/encouraged if feasible, but is strongly encouraged for participants with prior severe and/or clinically significant pulmonary disorders. In event of suspected ILD/pneumonitis, refer to Section 8.2.5.2.1 for additional pulmonary assessments.

High-resolution computed tomography (HRCT) of the chest is required at screening and if ILD/pneumonitis is suspected. Chest CT and/or chest HRCT scans will be reviewed separately for safety for the presence of ILD/pneumonitis prior to administration of the next scheduled dose of T-DXd. If both a non-contrast chest HRCT scan for assessment of ILD/pneumonitis and a diagnostic IV contrast enhanced chest CT scan for tumour response assessment (as part of chest-abdomen-pelvis imaging) are to be acquired in the same imaging session, HRCT should be performed first.

8.2.5.2.1 ILD/Pneumonitis Investigation

If new or worsening pulmonary symptoms (eg, dyspnoea, cough, or fever) or radiological abnormality suggestive of ILD/pneumonitis are observed, study intervention should be interrupted and a full investigation is required as described in the T-DXd TMGs (Appendix K). Evaluations should include:

- Signs and symptoms (cough, shortness of breath, and pyrexia, etc)
- Detailed past medical history, including concomitant medications
- Physical examination, including auscultation of lung field
- Arterial blood gases if clinically indicated
- Pulmonary function tests (Section 8.2.5.2) and pulse oximetry (SpO_2)
- Bronchoscopy and bronchoalveolar lavage, as clinically indicated and feasible
- HRCT
- Pulmonologist consultation (infectious disease consultation as clinically indicated)
- One blood sample collection for PK as soon as ILD/pneumonitis is suspected, if feasible
- Other tests could be considered, as needed.

The results of the full diagnostic workup (including HRCT, blood and sputum culture, haematological parameters, etc) will be captured in the eCRF. It is strongly recommended to perform a full diagnostic workup to exclude alternative causes such as lymphangitic carcinomatosis, infection, allergy, cardiogenic oedema, or pulmonary haemorrhage. In the presence of confirmatory HRCT scans where other causes of respiratory symptoms have been excluded, a diagnosis of ILD/pneumonitis should be considered and the TMGs should be followed. Troponin measurements will be performed to rule out cardiac aetiology.

The following assessments should be performed, if feasible, to enhance the investigation and diagnosis of potential cases of ILD/pneumonitis. The results of these assessment will be collected.

- Other items

  When ILD/pneumonitis is suspected during study intervention, the following markers should be measured where possible:
  - ILD/pneumonitis markers (Krebs von den Lungen-6 [KL-6] and surfactant protein D [(SP-D]) and β-D-glucan
  - Tumour markers: particular tumour markers related to disease progression
    - Additional clinical chemistry: C-reactive protein, lactate dehydrogenase.

8.2.5.3 ECOG Performance Status

ECOG performance status will be assessed at the times specified in the SoA (Section 1.3) using the following:

0 Fully active; able to carry out all usual activities without restrictions.

1 Restricted in strenuous activity, but ambulatory and able to carry out light work or work of a sedentary nature (eg, light housework or office work).

2 Ambulatory and capable of self-care, but unable to carry out any work activities; up and about more than 50% of waking hours.

3 Capable of only limited self-care; confined to bed or chair more than 50% of waking hours.

4 Completely disabled; unable to carry out any self-care and totally confined to bed or chair.

5 Dead.

Any significant change from baseline or screening must be reported as an AE.

8.2.5.4 Ophthalmological Assessments

Ophthalmological assessments will be performed as specified in the SoA (Section 1.3) and will include visual acuity testing, slit lamp examination, and fundoscopy.

8.2.5.5 Safety assessment for Eligible HBV infection only

- Eligible patients enrolled with inactive or resolved Hepatitis B infection will be monitored for reactivation.
- In patients enrolled with HBsAg(-) at baseline, reactivation is defined as:
  (1) reverse HBsAg seroconversion occurs (reappearance of HBsAg positivity)
  (2)HBV DNA > 2 log (100 x) baseline, or HBV DNA becomes detectable, if undetectable at baseline
  (3)Reactivation may occur with or without a hepatitis flare, reasonably defined as an ALT increase to ≥3 times the baseline level and >100 U/L.
- In patients with HBsAg(+) at baseline, reactivation will be defined as:
  1) HBV DNA > 2log (100 x) baseline or becomes detectable, if undetectable at baseline,
  (2)With or without a hepatitis flare (ALT increase to ≥3 times the baseline level and >100 U/L).

If HBV reactivation is confirmed, interrupt T-DXd therapy, and if not already on anti-viral therapy, commence prompt treatment with anti-virals following consultation with the local Hepatitis B expert and in accordance with local practice (administration of a potent nucleos(t)ide analogue with a high barrier to resistance i.e. entecavir, tenofovir disoproxil or tenofovir alafenamide is recommended). A decision to re-start T-DXd therapy should consider any potential interactions between T-DXd and therapies for HBV reactivation, and should be made in consultation with the AstraZeneca Study Physician and the supervising local Hepatitis B expert. Antivirals should be continued for at least 12 months after last dose of study treatment.

8.3 Adverse Events and Serious Adverse Events

The principal investigator is responsible for ensuring that all staff involved in the study are familiar with the content of this section.

The definitions of an AE or SAE can be found in Appendix B.

Adverse events will be reported by the participant (or, when appropriate, by a caregiver, surrogate, or the participant’s legally authorised representative).

The investigator and any designees are responsible for detecting, documenting, recording, and reporting events that meet the definition of an AE.

8.3.1 Time Period and Frequency for Collecting AE and SAE Information

Adverse events and SAEs will be collected from the time of signature of the ICF throughout the treatment period and including the safety follow-up period (which is 40 + 7 days after the discontinuation of study intervention). Additionally, for ILD/pneumonitis or reactivation of Hepatitis B, safety follow-up will be continued until resolution of ILD/pneumonitis or Hepatitis B reactivation. If an event that starts after the defined safety follow-up period is considered to be due to a late onset toxicity to study intervention, including reactivation of Hepatitis B infection, then it should be reported as an AE or SAE as applicable. Collection and reporting of AEs and SAEs after the final DCO is described in Section 8.3.12.

If the investigator becomes aware of an SAE with a suspected causal relationship to the study intervention that occurs after the end of the clinical study in a participant treated by him or her, the investigator shall, without undue delay, report the SAE to the sponsor.

A TEAE is defined as an AE that occurs, having been absent before the first dose of study intervention, or has worsened in severity or seriousness after the initiating the study intervention until 40 (+7) days after last dose of the study intervention. SAEs with an onset or worsening more than 40 (+7) days after the last dose of study intervention, if considered related to the study intervention, are also TEAEs.

The following types of events should be reported by the investigator in eCRF electronic data capture (EDC) AE page(s) in the clinical study database within 24 hours of becoming aware for the purposes of reporting in the global safety database:

- SAEs
- All potential ILD/pneumonitis cases should be reported within 24 hours; including both serious and non-serious potential ILD/pneumonitis cases (potential ILD/pneumonitis is described in the Event Adjudication Site Manual).
- All reactivation of Hepatitis B case (both serious and non-serious) should be reported within 24 hours.
- Hepatic events (both serious and non-serious) which meet the potential Hy's Law (PHL) criteria defined as an elevated (ALT or AST) $\geq 3 \times$ ULN <u>and</u> an elevated TBL $\geq 2 \times$ ULN that may occur either at different time points or simultaneously during the study. A targeted questionnaire is built within the eCRF to collect relevant additional information for these potential cases. See Section 8.3.6.
- Overdose, defined as the accidental or intentional administration of any dose of a product that is considered both excessive and medically important. An "excessive and medically important" overdose includes any overdose in which either a SAE, a non-serious AE, or no AE occurs and is considered by the investigator as clinically relevant, ie, poses an actual or potential risk to the participant.

- Overdose is always serious. By definition an overdose is medically important, which meets the seriousness criterion of important medical event. An overdose can occur with or without an AE. AEs can either be serious or non-serious. Details of the overdose including T-DXd dosage, clinical course, associated AEs, and outcome must be captured in the Narrative form of the eCRF within the EDC. See Section 8.4.

Disease progression/worsening of GC/GEJ adenocarcinoma will not be recorded as an AE on the Adverse Event eCRF. See Section 8.3.7.

Death due to disease progression should be recorded on the Death eCRF.

8.3.2 Follow-up of AEs and SAEs

Any AEs that are unresolved at the participant's last visit in the study are followed up by the investigator for as long as medically indicated, but without further recording in the eCRF. AstraZeneca retains the right to request additional information for any participant with ongoing AE(s)/SAE(s) at the end of the study, if judged necessary.

Adverse event variables

The following variables will be collected for each AE:

- AE (verbatim)
- The date the AE started and stopped
- Initial CTCAE grade, plus any changes in CTCAE grade
- Whether the AE is serious or not (Appendix B)
- Investigator causality rating against the study intervention (yes or no)
- Action taken with regard to study intervention
- AE caused participant's withdrawal from study (yes or no)
- Administration of treatment for the AE
- Outcome.

In addition, the following variables will be collected for SAEs:

- Date AE met criteria for SAE
- Date investigator became aware of SAE
- Seriousness criteria
- Date of hospitalisation
- Date of discharge
- Probable cause of death

- Date of death
- Autopsy performed
- Causality assessment in relation to study procedures
- Causality assessment to other medication.

The grading scales found in the NCI CTCAE will be utilised for all events with an assigned CTCAE grading. For those events without assigned CTCAE grades, the recommendation in the CTCAE criteria that converts mild, moderate, and severe events into CTCAE grades should be used. A copy of the CTCAE can be downloaded from the Cancer Therapy Evaluation Program website (http://ctep.cancer.gov).

8.3.3 Causality Collection

The investigator should assess causal relationship between study intervention and each AE, and answer "yes" or "no" to the question "Do you consider that there is a reasonable possibility that the event may have been caused by the investigational product?"

For SAEs, causal relationship should also be assessed for other medication and study procedures. Note that for SAEs that could be associated with any study procedure, the causal relationship is implied as "yes".

A guide to the interpretation of the causality question is found in Appendix B to the CSP.

8.3.4 Adverse Events Based on Signs and Symptoms

All AEs spontaneously reported by the participant or care provider or reported in response to the open question from the study site staff: "Have you had any health problems since the previous visit/since you were last asked?", or revealed by observation will be collected and recorded in the eCRF. When collecting AEs, the recording of diagnoses is preferred (when possible) to recording a list of signs and symptoms. However, if a diagnosis is known and there are other signs or symptoms that are not generally part of the diagnosis, the diagnosis and each sign or symptom will be recorded separately.

8.3.5 Adverse Events Based on Examinations and Tests

The results from the CSP-mandated laboratory tests, vital signs, physical examinations, ECGs, and ECHO/MUGA scans will be summarised in the Clinical Study Report (CSR).

Deterioration as compared with baseline in protocol-mandated laboratory values or vital signs should therefore only be reported as AEs if they fulfil any of the SAE criteria, are the reason for discontinuation of treatment with the study intervention, or are considered to be clinically relevant as judged by the investigator (which may include but not be limited to consideration as to whether treatment or non-planned visits were required or other action was taken with the

study intervention, eg, dose adjustment or study intervention interruption).

If deterioration in a laboratory value or vital sign is associated with clinical signs and symptoms, the sign or symptom will be reported as an AE and the associated laboratory result, or vital sign will be considered as additional information. Wherever possible the reporting investigator uses the clinical, rather than the laboratory term (eg, anaemia versus low haemoglobin value). Any diagnosis of the undesirable clinical outcome of 'Left ventricular dysfunction', a valid or qualifying reduction of LVEF (as measured by MUGA or ECHO) should be confirmed and included in the AE report. In the absence of clinical signs or symptoms, clinically relevant deteriorations in non-mandated parameters should be reported as AEs.

Deterioration of a laboratory value, which is unequivocally due to PD, should not be reported as an AE/SAE.

Any new or aggravated clinically relevant abnormal medical finding at a physical examination as compared with the baseline assessment will be reported as an AE unless unequivocally related to the disease under study.

8.3.6 Hy's Law

Cases where a participant shows elevations in liver biochemistry may require further evaluation and occurrences of AST or ALT $\geq 3 \times$ ULN together with TBL $\geq 2 \times$ ULN may need to be reported as SAEs. Please refer to Appendix D for further instruction on cases of increases in liver biochemistry and evaluation of HL.

8.3.7 Disease Progression

Disease progression can be considered as a worsening of a participant's condition attributable to the disease for which the study intervention is being studied. It may be an increase in the severity of the disease under study and/or increases in the symptoms of the disease. The development of new, or progression of existing metastasis to the primary cancer under study should be considered as PD and not an AE. Events, which are unequivocally due to PD, should not be reported as an AE during the study.

8.3.8 Disease Under Study

Symptoms of disease under study are those that might be expected to occur as a direct result of GC/GEJ adenocarcinoma. Events which are unequivocally due to disease under study should not be reported as an AE during the study unless they meet SAE criteria or lead to discontinuation of the study intervention.

8.3.9 New Cancers

The development of a new cancer should be regarded as an AE and will generally meet at least

one of the serious criteria (ie, an SAE). New primary cancers are those that are not the primary reason for the administration of study intervention and have been identified after the participant's inclusion in this study. They do not include metastases of the original cancer.

8.3.10 Deaths

All deaths that occur during the study intervention period, or within the protocol-defined follow-up period after the administration of the last dose of study intervention, must be reported as follows:

- Death clearly resulting from PD should be documented in the eCRF in the Statement of Death page. It should not be reported as an SAE.
- Where death is not due (or not clearly due) to progression of the disease under study, the AE causing the death must be reported as an SAE within 24 hours. It should also be documented in the Statement of Death page in the eCRF. The report should contain a comment regarding the co-involvement of PD, if appropriate, and should assign the main and contributory causes of death.
- Any death with an unknown cause should always be reported as an SAE and documented in the Statement of Death page in the eCRF, but every effort should be made to determine a cause of death. A post-mortem may be helpful in the assessment of the cause of death, and if performed, a copy of the post-mortem results should be forwarded to AstraZeneca Patient Safety or its representative within the usual time frames.

Deaths occurring after the protocol-defined follow-up period after the administration of the last dose of study intervention should be documented in the Statement of Death page. If the death occurred as a result of an event that started after the defined follow-up period and the event is considered to be due to a late-onset toxicity to study intervention, it should also be reported as an SAE.

8.3.11 Adverse Events of Special Interest

An AESI is an event of scientific and medical interest specific to the further understanding of the safety profile of T-DXd, and requires close monitoring and rapid communication by the investigators to AstraZeneca. An AESI can be serious or non-serious. All AESIs will be recorded in the eCRF using a recognised medical term or diagnosis that accurately reflects the event. Serious AESIs will be recorded and reported as per Section 8.3.13.

AESI will be assessed by the investigator for severity, relationship to the study intervention, possible aetiologies, and whether the event meets criteria for an SAE and therefore requires immediate notification to AstraZeneca. If an AESI evolves into a condition that meets the regulatory definition of "serious," it will be reported on the SAE Report Form.

Based on the available pre-clinical and clinical data, review of the cumulative literature, reported toxicities for the same class of agents, and biological plausibility, the following events are considered to be AESIs:

Interstitial Lung Disease/Pneumonitis

ILD is considered an important identified risk based on a comprehensive cumulative review of potential ILD/pneumonitis cases by the independent ILD Adjudication Committee, the available safety data from the clinical development programme, available data from recent epidemiology/literature, biological plausibility, and safety information from drugs of similar class. ILD event adjudication is a retrospective review and will not impact any safety decisions for participants. Refer to the current T-DXd IB for a summary of preliminary clinical study data.

LVEF Decrease

Left ventricular ejection fraction decrease in association with T-DXd is considered to be an important potential risk based on the available pre-clinical data, literature, and available safety information for drugs of a similar class. Refer to the current T-DXd IB for a summary of preliminary clinical trial data.

Refer to Sections 8.2.3 (ECGs and troponin testing) and 8.2.5.1 (ECHO/MUGA acquisition scans) for details of investigations of cardiac function.

Additional relevant information regarding the AESIs ILD/pneumonitis, and LVEF decrease, for the trastuzumab deruxtecan clinical program regardless of seriousness is to be collected through the specific section of the eCRF.

8.3.12 Safety Data to be Collected Following Final Study Data Cutoff

Where participants continue to receive study intervention following the final DCO, it is recommended that participants continue the scheduled site visits and investigators should continue to monitor and document data in the source notes. Thus, AEs and SAEs will continue to be collected, but only SAEs will be reported. In addition, it is recommended that investigators continue to monitor the participant's safety laboratory results during treatment with T-DXd in order to manage AEs, consistent with the dose modification guidelines for management of study intervention-related toxicities (see Section 6.6). All data after the final DCO and database closure will be recorded in the participant's notes but, with the exception of SAEs, will not otherwise be reported for the purposes of this study. Refer to Section 6.7 for further information.

All SAEs that occur in participants still receiving T-DXd (or within the 40 + 7 days follow-up period after the last dose of T-DXd) after the final DCO must be reported as detailed in Section 8.3.13.

8.3.13 Reporting of Serious Adverse Events

All SAEs have to be reported, whether or not considered causally related to the study intervention, or to the study procedure(s). All SAEs will be recorded in the eCRF.

If any SAE occurs in the course of the study, then investigators or other site personnel inform the appropriate AstraZeneca representatives within 1 day, ie, immediately but **no later than 24 hours** of when he or she becomes aware of it.

The designated AstraZeneca representative works with the investigator to ensure that all the necessary information is provided to the AstraZeneca Patient Safety data entry site **within 1 calendar day** of initial receipt for fatal and life-threatening events **and within 5 calendar days** of initial receipt for all other SAEs.

For fatal or life-threatening AEs where important or relevant information is missing, active follow-up is undertaken immediately. Investigators or other site personnel inform AstraZeneca representatives of any follow-up information on a previously reported SAE within 1 calendar day ie, immediately but **no later than 24 hours** of when he or she becomes aware of it.

Once the investigators or other site personnel indicate an AE is serious in the EDC system, an automated email alert is sent to the designated AstraZeneca representative.

If the EDC system is not available, the investigator or other study site staff reports a SAE to the appropriate AstraZeneca representative by telephone followed by completion of a paper SAE form. The AstraZeneca representative will advise the investigator/study site staff how to proceed.

For further guidance on the definition of a SAE, see Appendix B of the CSP.

The reference document for definition of expectedness/listedness for T-DXd is the current IB.

8.3.14 Pregnancy

All pregnancies and outcomes of pregnancy with conception dates following the first date of study intervention, including pregnancy in the partner of male participants, should be reported to AstraZeneca.

8.3.14.1 Maternal Exposure

If a participant becomes pregnant during the course of the study, the study intervention should be discontinued immediately.

Pregnancy itself is not regarded as an AE unless there is a suspicion that the study intervention under study may have interfered with the effectiveness of a contraceptive medication. Congenital abnormalities/birth defects and spontaneous miscarriages should be reported and

handled as SAEs. Elective abortions without complications should not be handled as AEs. The outcome of all pregnancies (spontaneous miscarriage, elective termination, ectopic pregnancy, normal birth or congenital abnormality) should be followed up and documented even if the participant was discontinued from the study.

If any pregnancy occurs during the study the investigator or other site personnel should inform the appropriate AstraZeneca representatives within 1 day, ie, immediately but **no later than 24 hours** of when he or she becomes aware of it.

The designated AstraZeneca representative will work with the investigator to ensure that all relevant information is provided to the AstraZeneca Patient Safety data entry site within 1 or 5 calendar days for SAEs (see Section 8.3.13) and within 30 days for all other pregnancies.

The same timelines apply when outcome information is available.

The PREGREP module in the eCRF is used to report the pregnancy.

8.3.14.2 Paternal Exposure

Non-sterilised male participants who intend to be sexually active with a female partner of childbearing potential should refrain from fathering a child or donating or banking sperm for the duration of the study (from the time of screening) and for 4 months after the last dose of study intervention.

Pregnancy of the participant's partner is not considered to be an AE. However, the outcome of all pregnancies (spontaneous miscarriage, elective termination, ectopic pregnancy, normal birth, or congenital abnormality) occurring from the date of the first dose of study intervention until 4 months after the last dose of study intervention should be followed up and documented in the medical record and provided to the AstraZeneca Patient Safety data entry site. Consent from the partner must be obtained before the information is collected and reported to AstraZeneca.

Where a report of pregnancy is received, prior to obtaining information about the pregnancy, the investigator must obtain the consent of the participant's partner. The local study team should adopt the Master Pregnant Partner Form in line with local procedures/requirements and submit it to the relevant Regulatory Authority/IRBs/IECs prior to use.

8.3.15 Medication Error

If a medication error occurs in the course of the study, the investigator or other site personnel should inform the appropriate AstraZeneca representatives within 1 day, ie, immediately but no later than 24 hours of when he or she becomes aware of it.

The designated AstraZeneca representative will work with the investigator to ensure all

relevant information is completed within 1 (Initial Fatal/Life-threatening or follow-up Fatal/Life-threatening) or 5 (other serious initial and follow-up) calendar days if there is an SAE associated with the medication error (see Section 8.3.13), and within 30 days for all other medication errors.

Medication errors are defined in Appendix B.

8.4 Overdose

Use of T-DXd in doses exceeding that specified in the CSP is considered to be an overdose. There is currently no specific treatment in the event of overdose of T-DXd, and possible symptoms of overdose are not established.

- An overdose with associated AEs is recorded as the AE diagnosis/symptoms on the relevant AE modules in the eCRF and on the Overdose eCRF module.
- An overdose without associated symptoms is only reported on the Overdose eCRF module.

If an overdose on an AstraZeneca study intervention occurs in the course of the study, the investigator or other site personnel should inform appropriate AstraZeneca representatives immediately, but **no later than 24 hours** after he or she becomes aware of it.

The designated AstraZeneca representative will work with the investigator to ensure that all relevant information is provided to the AstraZeneca Patient Safety data entry site within 1 or 5 calendar days for overdoses associated with an SAE (see Section 8.3.13) and within 30 days for all other overdoses.

8.5 Human Biological Samples

Instructions for the collection, handling, storage and shipping of biological samples will be provided in the study-specific laboratory manual. Samples should be stored in a secure storage space with adequate measures to protect confidentiality.

Samples collected in China will be stored and disposed of according to local laws and regulations. PK and anti-drug antibody (ADA) samples collected in China will be destroyed after finalisation of the Bioanalytical Report or completion of the CSR.

For further details on Handling of Human Biological Samples, see Appendix C.

8.5.1 Pharmacokinetics

- Serum samples will be collected for measurement of serum concentrations of T-DXd, total anti-HER2 antibody, and MAAA-1181a as specified in the SoA and Appendix H.

- Samples may be collected at additional time points during the study if warranted and agreed upon between the investigator and the sponsor, for example, for safety reasons. The timing of sampling may be altered during the course of the study based on newly available data (eg, to obtain data closer to the time of peak or trough matrix concentrations) to ensure appropriate monitoring.
- Serum samples will be used to analyse the PK of T-DXd, total anti-HER2 antibody, and MAAA-1181a. Samples collected for analyses of T-DXd, total anti-HER2 antibody, and MAAA-1181a concentration may also be used to evaluate safety or efficacy aspects related to concerns arising during or after the study.

8.5.1.1 Determination of Drug Concentration

Samples for determination of drug concentration in serum will be assayed by bioanalytical test sites operated by or on behalf of AstraZeneca, using an appropriately validated bioanalytical method. Full details of the analytical method used will be described in a separate Bioanalytical Report.

Incurred sample reproducibility analysis, if any, will be performed alongside the bioanalysis of the test samples. The results from the evaluation, if performed, will be reported in a separate Bioanalytical Report.

8.5.2 Immunogenicity Assessments

Blood samples for determination of ADAs against T-DXd and neutralising ADAs in serum will be collected per the SoA (Section 1.3). Samples will be assayed by bioanalytical test sites operated by or on behalf of AstraZeneca, using an appropriately validated bioanalytical method. Full details of the methods used will be described in a separate report.

ADA samples may also be further tested for characterisation of the ADA response. Please also refer to Appendix H.

8.6 Human Biological Sample Biomarkers

8.6.1 Collection of Mandatory Samples for Central Biomarker Analysis

Participant consent to the study includes participation in the mandatory biomarker assessment components of the study. FFPE tumour slides are mandatory in order to confirm HER2 status and will be collected from all enrolled participants as outlined below and in the SoA (Section 1.3).

FFPE tumour tissue slides must be provided for each enrolled participant after enrolment and prior to first dose of study intervention, in order to confirm HER2 status by central laboratory. It can be either:

- A newly acquired tumour sample if the primary tumour is accessible by endoscopy or if metastatic tissue can be biopsied, or
- The most recent archived tumour tissue sample, if a newly-acquired sample is not available.

If a tumour biopsy was performed at screening in order to determine participant's HER2 status locally during screening, the same biopsy will be provided to the central laboratory for confirmation of HER2 status. If the participant's HER2 status has not been documented locally, the newly-acquired biopsy obtained from an accessible tumour should be used to confirm HER2 status locally, and used to confirm HER2 status by central laboratory.

For all FFPE tumour specimens, freshly-cut, unstained, serial tumour slides of sufficient quality to allow for assessment of HER2 status should be submitted. Please note:

- The mandatory screening tumour biopsy must not be taken from a previously irradiated lesion.
- Tumour lesions used for newly-acquired biopsies should not be the same lesions used as RECIST 1.1 target lesions, unless there are no other lesions suitable for biopsy and in this instance only core needle (not excisional/incisional) biopsy is allowed.

To meet regulatory requirement, samples collected and testing data generated may be used to support companion diagnostic development and registration for the disease.

FFPE tumour slides collected for central HER2 confirmatory testing will be destroyed or repatriated maximally 5 years after study intervention is approved for marketing in China. To meet regulatory requirement, sections of the tumor may be retained that allow this for potential companion diagnostic filing as requested by the NMPA.

The histological images taken by the central laboratory may be used for computer-aided analysis to explore clinical response-associated biomarkers.

For further details on Handling of Human Biological Samples, including storage and destruction, refer to Appendix C and the Laboratory Manual. Details of tumour sampling requirements are provided in the Laboratory Manual.

9 STATISTICAL CONSIDERATIONS

Statistical analyses will be performed by AstraZeneca or its representatives.

A comprehensive SAP will be prepared prior to enrolment of the first participant in the study.

9.1 Statistical Hypotheses

This is a single-arm, Phase II open-label study in which all participants will receive the same study intervention. The primary objective of the study is to evaluate the efficacy of T-DXd 6.4 mg/kg by assessing the confirmed ORR by ICR in participants with HER2-positive (defined as IHC 3+ and IHC 2+/ISH +) advanced gastric or GEJ adenocarcinoma who have received at least 2 prior treatment regimens including a fluoropyrimidine and a platinum agent. The secondary objective is to evaluate the efficacy of T-DXd 6.4 mg/kg by assessing the confirmed ORR by ICR in participants with HER2-expressing (defined as IHC 3+ and IHC 2+) advanced gastric or GEJ adenocarcinoma who have received at least 2 prior treatment regimens including a fluoropyrimidine and a platinum agent.

The primary endpoint of the study, confirmed ORR by ICR, is defined as the proportion (%) of participants with confirmed CR or confirmed PR as assessed by ICR per RECIST 1.1.

Although participant eligibility with respect to HER2-positive status will be determined locally, the primary analysis will be performed on participants whose HER2-positive status is confirmed by central laboratory.

In view of the open-label, single-arm study design, no formal statistical hypothesis will be tested and, unless otherwise specified, study data will be presented using descriptive statistics. However, to ensure strong control of type 1 error amongst the primary and secondary ORR endpoints in the HER2-positive and HER2-expressing populations, a gated approach will be applied to the analysis of ORR in the HER2-expressing population. The analysis of ORR in the HER2-expressing population will only be assessed for success if the analysis of ORR in the HER2-positive population is deemed successful. See Section 9.4.5 for details. Efficacy demonstrated in confirmed ORR by ICR per RECIST 1.1 is considered an early signal for clinical benefit.

9.2 [redacted]

[redacted]

[redacted]

9.3 Populations for Analyses

Analysis populations are defined in Table 11 for analyses to support the primary objective in HER2 positive population (HER2 Positive FAS, HER2-Positive RES) and to support the secondary objectives in HER2-expressing population (HER2-expressing FAS, HER2-expressing RES).

Table 11 Populations for Analysis

Population/Analysis Set	Description
ITT Analysis Set	All participants who signed the informed consent form and were enrolled in the study
HER2- Positive FAS	All enrolled participants with HER2 status confirmed as IHC 3+ or IHC 2+/ISH + by central laboratory
HER2-expressingFAS	All enrolled participants with HER2 status confirmed as IHC 3+ or IHC 2+ by central laboratory
HER2- Positive RES	All enrolled participants with HER2 status confirmed as IHC 3+ or IHC 2+/ISH + by central laboratory who received at least 1 dose of study intervention and had measurable disease at baseline by ICR
HER2-expressing RES	All enrolled participants with HER2 status confirmed as IHC 3+ or IHC 2+ by central laboratory who received at least 1 dose of study intervention and had measurable disease at baseline by ICR
Safety Analysis Set (SAF)	All enrolled participants who received at least 1 dose of study intervention
PK Analysis Set	All enrolled participants who received at least 1 dose of study intervention and had at least 1 post-dose measurable serum concentration of T-DXd
T-DXd ADA Evaluable Set	All participants in the Safety Analysis Set with a non-missing baseline ADA T-DXd result and at least 1 post-baseline ADA T-DXd result

ADA = anti-drug antibody; FAS = full analysis set; HER2 = human epidermal growth factor receptor 2; ICR = independent central review; IHC = immunohistochemistry; ISH = in situ hybridisation; ITT = intent to treat; PK = pharmacokinetic; RES = response evaluable set; T-DXd = trastuzumab deruxtecan.

9.4 Statistical Analyses

9.4.1 General Considerations

The SAP will be finalised prior to enrolment of the first participant in the study and will include a more technical and detailed description of the statistical analyses described in this section. This section is a summary of the planned statistical analyses of the most important endpoints including primary and key secondary endpoints. Table 12 provides a summary of key endpoints and corresponding analysis populations.

The DCO for the primary analysis will occur approximately [redacted] after the last participant has initiated study intervention. At this time, [redacted] data will also be summarised.

The DCO for the full final analysis will occur approximately [redacted] months after the last participant has initiated study intervention. Given the median time to response [redacted] and the median duration of response [redacted] among participants with confirmed CR or PR in the T-DXd [redacted], this provides sufficient time for participants to reach response and sufficient DoR follow-up for responders. The full final analysis will report the analyses of all primary and secondary endpoints, including updated [redacted]

Table 12 Summary of Outcome Variables and Analysis Populations

Outcome variable	Populations
**Primary Efficacy Variable in HER2 Positive Population**	
• Confirmed ORR by ICR per RECIST 1.1	Primary analysis: HER2 Positive FAS<br>Supplementary analysis: HER2 Positive RES
**Secondary Efficacy Variables in HER2 Positive Population**	
• Confirmed ORR by investigator assessment per RECIST 1.1<br>• PFS, DoR, DCR, and tumour size change by ICR and by investigator assessment per RECIST 1.1<br>• OS	HER2 Positive FAS
**Secondary Efficacy Variables in HER2-expressing Population**	
• Confirmed ORR by ICR per RECIST 1.1<br>• Confirmed ORR by investigator per RECIST 1.1<br>• PFS, DoR, DCR, and tumour size change by ICR and by investigator assessment per RECIST 1.1<br>• OS	Primary analysis: HER2-expressing FAS<br>Supplementary analysis: HER2-expressing RES
**Baseline and Other Variables**	
• Demography, baseline, and disease characteristics<br>• Important deviations<br>• Medical/surgical history<br>• Previous and subsequent anticancer therapy<br>• Concomitant medications/procedures	Primary analysis: HER2-Positive FAS<br>Supplementary analysis: ITT Analysis Set
**Pharmacokinetics**	
• Pharmacokinetics data	PK Analysis Set
**Immunogenicity**	
• Anti-drug antibodies	T-DXd ADA Evaluable Set
**Safety**	
• Exposure to study intervention<br>• Safety data	Safety Analysis Set

ADA = anti-drug antibody; DCR = disease control rate; DoR = duration of response; FAS = full analysis set; ICR = independent central review; ITT = intent to treat; ORR = objective response rate; OS = overall survival; PFS = progression-free survival; PK = pharmacokinetic; RECIST 1.1 = Response Evaluation Criteria in Solid Tumours version 1.1; RES = response evaluable set; T-DXd = trastuzumab deruxtecan.

9.4.2 Efficacy

9.4.2.1 Calculation or Derivation of Tumour Response Variable

Independent Central Review

An ICR of radiological scans will be performed on all participants.

All images will be collected centrally. The imaging scans will be reviewed by 2 independent radiologists using RECIST 1.1 and will be adjudicated, if required. For each participant, the ICR will define the overall visit response data (CR, PR, SD, PD, or NE) and the relevant scan dates for each time point (ie, for visits where response or progression is/is not identified). If a participant has had a tumour assessment that cannot be evaluated, then the participant will be assigned a visit response of NE (unless there is evidence of progression, in which case the response will be assigned as PD). Efficacy endpoints (ORR, PFS, DoR, and DCR) by ICR will then be derived from the scan dates and overall visit responses.

Further details of the ICR will be documented in an Independent Review Charter.

Investigator Assessments per RECIST 1.1

All RECIST assessments, whether scheduled or unscheduled, will be included in the calculations. This is also regardless of whether a participant discontinues study intervention or receives another anticancer therapy.

At each visit, participants will be programmatically assigned a RECIST 1.1 visit response of CR, PR, SD, PD, or NE depending on the status of their disease compared with baseline and previous assessments. Baseline will be assessed within the 28 days prior to study enrolment.

Refer to Appendix E for definitions of CR, PR, SD, PD, and NE.

9.4.2.2 Primary Endpoint: Confirmed ORR by ICR per RECIST 1.1

The primary endpoint of the study is confirmed ORR by ICR according to RECIST 1.1. Confirmed ORR (per RECIST 1.1 by ICR) is defined as the percentage of participants with a confirmed response of CR or PR. Data obtained up until progression or the last evaluable assessment with the absence of progression will be included in the assessment of ORR. Participants who discontinue study intervention without progression, receive a subsequent anticancer therapy, and then respond will not be included as responders in the calculation of ORR.

A confirmed response of CR or PR means that a response of CR or PR is recorded at 1 visit and confirmed by repeat imaging not less than 4 weeks after the response was first observed, with no evidence of progression between the initial and CR/PR confirmation visits.

Confirmed Best Objective Response by ICR

Best objective response (BOR) is a participant's best response during their participation in the study, but prior to starting any subsequent anticancer therapy, up until RECIST 1.1-defined progression or the last evaluable assessment in the absence of RECIST 1.1-defined progression.

Categorisation of BOR will be based on RECIST 1.1 by ICR using the following response categories: (confirmed) CR, (confirmed) PR, SD, PD, and NE; unconfirmed CR/PR will be included in SD.

Analyses of Primary Endpoint

The primary analysis of the primary endpoint will be based on the HER2 Positive FAS. ORR will be estimated with 2-sided 95% exact CI. Summaries will be produced presenting the number and percentage of participants with a confirmed tumor response. Confirmed BOR will be summarized descriptively by n (%) for each category (confirmed CR, confirmed PR, SD, PD, and NE).

Supplementary Analysis

Supplementary analysis of the primary endpoint will be performed in the HER2 Positive RES using the same methods as described above.

Sensitivity Analysis

Details of any sensitivity analyses to be conducted for the primary endpoint will be described in the SAP, as appropriate.

Subgroup Analysis

Details of any subgroup analyses to be conducted for the primary endpoint will be specified in the SAP.

9.4.2.3 Secondary Endpoints in HER2 Positive Population

9.4.2.3.1 Objective Response Rate by Investigator Assessment

The secondary efficacy endpoint of ORR is defined as the proportion of participants who have a confirmed CR or PR, as determined by the investigator at local site per RECIST 1.1.

Confirmed Best Objective Response by Investigator Assessment

BOR is calculated based on the overall visit responses per RECIST 1.1 using investigator assessments. BOR is a participant's best response during their participation in the study, but prior to starting any subsequent anticancer therapy, up until RECIST 1.1-defined progression or the last evaluable assessment in the absence of RECIST 1.1-defined progression.

Categorisation of BOR will be based on RECIST 1.1 by investigator assessment using the following response categories: (confirmed) CR, (confirmed) PR, SD, PD, and NE; unconfirmed CR/PR will be included in SD.

Refer to Appendix E for guidance on the evaluation of tumor response per RECIST 1.1.

BOR will be determined programmatically based on RECIST 1.1 using all investigator assessments up until the first progression event per RECIST 1.1 as determined by the investigator. For participants whose first progression event is death, BOR will be calculated based on all evaluable RECIST 1.1 assessments prior to death.

For participants who die with no evaluable RECIST 1.1 assessments, if death occurs ≤ 91 days (ie, 2 * (6 weeks) + 1 week) after the first dose of study intervention, then BOR will be assigned to the PD category. For participants who die with no evaluable RECIST assessments, if the death occurs > 91 days (ie, 2 * (6 weeks) + 1 week) after the date of the first dose of study intervention, BOR will be assigned to the NE category.

Analysis Methods

Investigator assessed ORR will be summarized using the same methods as those specified for ORR by ICR for the HER2 Positive FAS (see Section 9.4.2.2).

9.4.2.3.2 Progression-free Survival

Progression-free Survival by ICR

PFS by ICR is defined as the time from enrolment until the date of confirmed PD per RECIST 1.1 as assessed by ICR or death (by any cause in the absence of progression) (ie, date of event or censoring – date of the first dose of study intervention + 1). The analysis will include all participants regardless of whether the participant withdraws from study intervention, receives another anticancer therapy, or clinically progresses prior to RECIST 1.1 progression. The date of progression will be determined based on the earliest of the scan dates of the component that triggered the progression. Participants who have not progressed or died at the time of analysis will be censored at the time of the latest date of assessment from their last evaluable RECIST 1.1 assessment. However, if the participant progresses or dies after 2 or more consecutive missed visits, the participant will be censored at the time of the latest evaluable RECIST 1.1 assessment prior to the 2 missed visits. (Note: NE visit is not considered as missed visit).

If the participant has no evaluable visits or does not have baseline data, they will be censored at Day 1, unless they die within 2 visits of baseline, ie, ≤ 91 days (2 * (6 weeks) + 1 week) allowing for a late assessment within the visit window).

Progression-free Survival by Investigator Assessment

PFS by investigator assessment is defined as the time from enrolment until the date of confirmed PD per RECIST 1.1 as assessed by the investigator or death (by any cause in the absence of progression) (ie, date of event or censoring – date of the first dose of study intervention + 1).

Data will be handled as described above for PFS by ICR.

Analysis Methods

PFS will be summarised for the HER2 Positive FAS. A Kaplan-Meier plot of PFS will be presented. The estimate of median PFS and corresponding 95% CI using the Brookmeyer-Crowley method with log-log transformation will be reported (Brookmeyer and Crowley 1982, Klein et al 1997). Summaries of the number and percentage of participants experiencing a PFS event and the type of event (RECIST 1.1 PD or death) will be provided. The proportion of participants alive and progression-free at 3-monthly intervals from the first dose of study intervention will be summarized.

9.4.2.3.3 Overall Survival

OS is defined as the time from the date of enrolment until death due to any cause. Any participant not known to have died at the time of analysis will be censored based on the last recorded date on which the participant was known to be alive.

Analysis Methods

OS will be analyzed for the HER2 Positive FAS.

A Kaplan Meier plot of OS will be presented. Summaries of the number and percentage of participants with an OS event will be provided along with the estimate of median OS and corresponding 95% CI using the Brookmeyer-Crowley method with log-log transformation (Brookmeyer and Crowley 1982, Klein et al 1997). The proportion of participants alive at 3-monthly intervals from the first dose of study intervention will be summarised.

9.4.2.3.4 Duration of Response

Duration of Response by ICR

For participants who achieve a confirmed CR/PR per RECIST 1.1 by ICR, DoR is defined as the time from the date of first documented response until the date of documented progression (using RECIST 1.1 by ICR) or death in the absence of disease progression. The time of the initial response will be defined as the latest of the dates contributing towards the first visit response of CR or PR. The end of response should coincide with the date of progression or death from any cause used for the PFS endpoint. If a participant does not progress following a response, then their DoR will be the PFS censoring time (ie, DoR = date of PFS event or censoring - date of first response + 1).

Duration of Response by Investigator Assessment

For participants who achieve a confirmed CR/PR per RECIST 1.1 by investigator assessment, DoR is defined as the time from the date of first documented response until the date of documented progression (using RECIST 1.1 by investigator assessment) or death in the absence of disease progression.

Data will be handled as described above for DoR by ICR.

Analysis Methods

DoR will be analysed for the HER2 Positive FAS.

A Kaplan Meier plot of DoR will be presented. The estimate of median DoR and corresponding 95% CI using the Brookmeyer-Crowley method with log-log transformation will be reported (Brookmeyer and Crowley 1982, Klein et al 1997).

9.4.2.3.5 Disease Control Rate

Disease Control Rate by ICR

The DCR by ICR at 6 weeks is defined as the percentage of participants who have a confirmed CR/PR or SD for at least 5 weeks (ie, 6 weeks - 1 week to allow for an early assessment within the assessment window) after the date of enrolment (without subsequent anticancer therapy) per RECIST 1.1 by ICR.

The DCR by ICR at 24 weeks is defined as the percentage of participants who have a confirmed CR/PR or SD for at least 23 weeks (ie, 24 weeks - 1 week to allow for an early assessment within the assessment window) after the date of enrolment (without subsequent anticancer therapy) per RECIST 1.1 by ICR.

Disease Control Rate by Investigator Assessment

The DCR by investigator assessment at 6 weeks is defined as the percentage of participants who have a confirmed CR/PR or SD for at least 5 weeks (ie, 6 weeks - 1 week to allow for an early assessment within the assessment window) after the date of enrolment (without subsequent anticancer therapy) per RECIST 1.1 by investigator assessment.

The DCR by investigator assessment at 24 weeks is defined as the percentage of participants who have a confirmed CR/PR or SD for at least 23 weeks (ie, 24 weeks - 1 week to allow for an early assessment within the assessment window) after the date of enrolment (without subsequent anticancer therapy) per RECIST 1.1 by investigator assessment.

Analysis Methods

DCR will be summarised descriptively with the number and percentage of participants with a confirmed CR/PR or SD for the HER2-Positive FAS.

9.4.2.4 Secondary Endpoints in HER2-expressing Population

Secondary efficacy endpoints in the HER2-expressing population including confirmed ORR, PFS, DoR, DCR, and tumour size change by ICR and by investigator assessment per RECIST 1.1, and OS will be analysed in HER2-expressing RES as primary analysis population and HER2-expressing FAS as supplementary analysis population using corresponding methods specified in section 9.4.2.3 to support the secondary objective in the HER2- expressing population. Details of the planned analysis, including subgroup and sensitivity analysis will be specified in the statistical analysis plan.

In order to ensure that family-wise type 1 error is controlled, the analysis of ORR in the HER2-expressing population will only be assessed for success if the analysis of ORR in the HER2-positive population is deemed successful, details see section 9.4.5.

9.4.3 Safety

Safety summaries will be provided using the Safety Analysis Set. Safety data will be presented using descriptive statistics unless otherwise specified. Summary statistics for continuous variables will include number of participants, mean, standard deviation, minimum, median, and maximum. Frequency tables and shift tables will include number and percentage of participants in the respective category. Unless otherwise stated, percentages will be calculated out of the population total.

Baseline

In general, the baseline value any parameter is the last non-missing value prior to administration of the first dose of study intervention. Details are described in the SAP.

Adverse Events

Adverse events will be coded using the most recent version of MedDRA that will be released for execution at AstraZeneca and NCI CTCAE v5.0.

Any TEAE occurring until 40 (+ 7) days after the last dose of the study intervention and prior to the start of a new anticancer treatment will be included in the AE summaries. Any other AEs will be flagged in the data listings, but not included in the summaries.

An overview of TEAEs will be provided: the number and percentage of participants with any TEAE, TEAEs with outcome of death, serious TEAEs, and TEAEs leading to discontinuation of study intervention, as well as TEAEs leading to study intervention dose interruptions, and AEs leading to study intervention dose reduction.

TEAEs will be presented by System Organ Class and/or Preferred Term covering number and percentage of participants reporting at least one event and number of events where appropriate.

Separate TEAE tables will be provided taking into consideration the relationship to study intervention as assessed by the investigator, the CTCAE grade, seriousness, death and events leading to discontinuation of study intervention as well as other action taken related to study intervention, AESIs, and other significant TEAEs (if applicable).

An additional table will be presented for the number and percentage of participants with most common TEAEs. Most common TEAEs will be defined in the SAP.

A TEAE listing will cover details for each individual TEAE.

Adverse events occurring prior to start of study intervention, TEAEs and post-treatment AEs will be presented separately.

Full details of TEAE analyses will be provided in the SAP.

Vital Signs

For each scheduled post-baseline visit, descriptive statistics for all vital sign parameters will be presented for observed values and change from baseline.

Details of vital sign analyses will be provided in the SAP.

ECGs

For each scheduled post-baseline visit, descriptive statistics for all ECG parameters will be presented for observed values and change from baseline. QTcF will be derived during creation of the reporting database using reported ECG values (RR and QT) using the following formula, where RR is in seconds:

$$QTcF = \frac{QT}{\sqrt[3]{RR}}$$

Details of ECG analyses will be provided in the SAP.

Laboratory Parameters

For each scheduled post-baseline visit, descriptive statistics for all clinical chemistry and haematology parameters will be presented for observed values and change from baseline.

Elevation in liver parameters for assessment of HL will be evaluated and reported appropriately if potential cases are identified during the course of the study. Corrected calcium will be derived during creation of the reporting database using the following formula:

$$\text{Corrected calcium (mmol/L)} = \text{total calcium (mmol/L)} + ([40 - \text{albumin (g/L)}] \times 0.02)$$

A shift table for urinalysis will be presented with baseline assessment against the maximum on-study intervention category.

Supportive laboratory listings will cover observed values and changes from baseline for each individual participant as well as abnormalities.

Details of laboratory summaries will be provided in the SAP.

Other Safety Analyses

All other safety endpoints, eg, physical examination findings including ECOG performance status, elevated troponin levels, ECHO/MUGA, and ophthalmologic findings, will be listed. Further details will be provided in the SAP.

9.4.4 Other Analyses

9.4.4.1 Pharmacokinetics

Serum PK concentration data for T-DXd, total anti-HER2 antibody, and MAAA-1181a will be listed for each sampling time for each participant at each sampling point, and a summary will be provided for all participants in the PK Analysis Set. Descriptive statistics may be calculated.

Details of the PK analyses will be described in the SAP prior to enrolment of the first participant in the study.

The popPK analysis to evaluate the effect of intrinsic and extrinsic factors of T-DXd, and if appropriate, total anti-HER2 antibody and MAAA-1181a will be reported separately from the main CSR.

9.4.4.2 Immunogenicity Data

Anti-drug antibody data will be summarized using the T-DXd ADA Evaluable Set. Immunogenicity results will be listed by participant, and a summary will be provided by the number and percentage of ADA-evaluable participants who develop detectable anti-T-DXd antibodies during the study. The immunogenicity titre and neutralising ADA data will be listed for samples confirmed positive for the presence of anti-T-DXd antibodies.

The effect of immunogenicity as well as the effect of its neutralising properties on PK, pharmacodynamics, efficacy, and safety will be evaluated, if the data allow. A detailed plan will be written by the AstraZeneca Clinical Pharmacology group or designee.

9.4.4.3 COVID-19

Summaries of data relating to participants diagnosed with COVID-19, and the impact of COVID-19 on study conduct (in particular missed visits, delayed or discontinued study intervention, and other protocol deviations) may be generated. More detail will be provided in

the SAP.

9.4.5 Methods for Multiplicity Control

[redacted].

The analysis of ORR in the [redacted] will only be assessed for success if the analysis of ORR in the [redacted] is deemed successful. The success criterion for ORR endpoint in the [redacted] is defined as that the [redacted] ORR of T-DXd [redacted] The success criterion for ORR endpoint [redacted] is defined as that the [redacted] of [redacted]

[redacted]

9.5 Interim Analyses

An interim analysis is not planned in this study.

9.6 Data Monitoring Committees

The safety of all AstraZeneca clinical studies is closely monitored on an ongoing basis by AstraZeneca representatives in consultation with Patient Safety. Issues identified will be addressed; for instance, this could involve amendments to the CSP and letters to investigators. An Independent Data Monitoring Committee is not considered necessary for this open-label, single-arm study.

9.6.1 ILD Adjudication Committee

An ILD Adjudication Committee and Charter will be established to review all cases of potential ILD/pneumonitis. To ensure adequate evaluation, relevant additional data from within the clinical database may be provided to the adjudication committee to fully characterise medical history (eg, smoking, radiation and pulmonary history), diagnostic evaluation, treatment, and outcome of the event. Further details can be found in the ILD Adjudication Charter.

10 SUPPORTING DOCUMENTATION AND OPERATIONAL CONSIDERATIONS

Appendix A Regulatory, Ethical, and Study Oversight Considerations

A 1 Regulatory and Ethical Considerations

- This study will be conducted in accordance with the protocol and with the following:
  - Consensus ethical principles derived from international guidelines including the Declaration of Helsinki and Council for International Organizations of Medical Sciences International Ethical Guidelines
  - Applicable ICH GCP Guidelines
  - Applicable laws and regulations
- The protocol, protocol amendments, ICF, IB, and other relevant documents (eg, advertisements) must be submitted to an IRB/IEC by the investigator and reviewed and approved by the IRB/IEC before the study is initiated.
- Any amendments to the protocol will require IRB/IEC and applicable Regulatory Authority approval before implementation of changes made to the study design, except for changes necessary to eliminate an immediate hazard to study participants.
- AstraZeneca will be responsible for obtaining the required authorisations to conduct the study from the concerned Regulatory Authority. This responsibility may be delegated to a Contract Research Organisation but the accountability remains with AstraZeneca.

Regulatory Reporting Requirements for Serious Adverse Events

- Prompt notification by the investigator to the sponsor of a SAE is essential so that legal obligations and ethical responsibilities towards the safety of participants and the safety of a study intervention under clinical investigation are met.
- The sponsor has a legal responsibility to notify both the local regulatory authority and other regulatory agencies about the safety of a study intervention under clinical investigation. The sponsor will comply with country-specific regulatory requirements relating to safety reporting to the regulatory authority, IRB/IEC, and investigators.
- For all studies except those utilising medical devices investigator safety reports must be prepared for suspected unexpected serious adverse reactions according to local regulatory requirements and sponsor policy and forwarded to investigators as necessary.
- An investigator who receives an investigator safety report describing a SAE or other specific safety information (eg, summary or listing of SAEs) from the sponsor will review and then file it along with the [IB or state other documents] and will notify the IRB/IEC, if appropriate according to local requirements.

A 2 Financial Disclosure

Investigators and sub-investigators will provide the sponsor with sufficient, accurate financial

information as requested to allow the sponsor to submit complete and accurate financial certification or disclosure statements to the appropriate regulatory authorities. Investigators are responsible for providing information on financial interests during the course of the study and for 1 year after completion of the study.

A 3 Informed Consent Process

- The investigator or his/her representative will explain the nature of the study to the participant or his/her legally authorised representative and answer all questions regarding the study.
- Participants must be informed that their participation is voluntary and they are free to refuse to participate and may withdraw their consent at any time and for any reason during the study. Participants or their legally authorised representative will be required to sign a statement of informed consent that meets the requirements of 21 Code of Federal Regulations 50, local regulations, ICH guidelines, Health Insurance Portability and Accountability Act requirements, where applicable, and the IRB/IEC or study centre.
- The medical record must include a statement that written informed consent was obtained before the participant was enrolled in the study and the date the written consent was obtained. The authorised person obtaining the informed consent must also sign the ICF.
- Participants must be re-consented to the most current version of the ICF(s) during their participation in the study.
- A copy of the ICF(s) must be provided to the participant or the participant's legally authorised representative.

Participants who are rescreened are required to sign a new ICF.

A 4 Data Protection

- Participants will be assigned a unique identifier by the sponsor. Any participant records or datasets that are transferred to the sponsor will contain the identifier only; participant names or any information which would make the participant identifiable will not be transferred.
- The participant must be informed that his/her personal study-related data will be used by the sponsor in accordance with local data protection law. The level of disclosure and use of their data must also be explained to the participant in the informed consent
- The participant must be informed that his/her medical records may be examined by Clinical Quality Assurance auditors or other authorised personnel appointed by the sponsor, by appropriate IRB/IEC members, and by inspectors from regulatory authorities.

Unless previously specified, the biomarker data will have unknown clinical significance and

AstraZeneca will not provide biomarker assessment results to participants, their family members, any insurance company, any employer, a clinical study investigator, a general physician, or any other third party, unless required to do so by law; however, AstraZeneca may share data and biosamples with research partners, eg, Daiichi Sankyo.

The participant's samples will not be used for any purpose other than those described in the study protocol.

A 5 Committees Structure

The safety of all AstraZeneca clinical studies is closely monitored on an on-going basis by AstraZeneca representatives in consultation with Patient Safety. Issues identified will be addressed; for instance, this could involve amendments to the CSP and letters to investigators.

A 6 Dissemination of Clinical Study Data

A description of this clinical study will be available on http://astrazenecaclinicaltrials.com, http://www.clinicaltrials.gov, and https://www.clinicaltrialsregister.eu/, as will the summary of the main study results when available. The clinical study and/or summary of main study results may also be available on www.chinadrugtrials.org.cn according to the regulations of China, the country in which the study is conducted.

A 7 Data Quality Assurance

- All participant data relating to the study will be recorded on the eCRF unless transmitted to the sponsor or designee electronically (eg, laboratory data). The investigator is responsible for verifying that data entries are accurate and correct by physically or electronically signing the eCRF.
- The investigator must maintain accurate documentation (source data) that supports the information entered in the eCRF.
- The investigator must permit study-related monitoring, audits, IRB/IEC review, and regulatory authority inspections and provide direct access to source data documents.
- Monitoring details describing strategy (eg, risk-based initiatives in operations and quality such as Risk Management and Mitigation Strategies and Analytical Risk-Based Monitoring), methods, responsibilities and requirements, including handling of noncompliance issues and monitoring techniques (central, remote, or on-site monitoring) are provided in the Monitoring Plan.
- The sponsor or designee is responsible for the data management of this study including quality checking of the data.
- The sponsor assumes accountability for actions delegated to other individuals (eg, Contract Research Organisations).

- Study monitors will perform ongoing source data verification to confirm that data entered into the eCRF by authorised site personnel are accurate, complete, and verifiable from source documents; that the safety and rights of participants are being protected; and that the study is being conducted in accordance with the currently approved protocol and any other study agreements, ICH GCP, and all applicable regulatory requirements.
- Records and documents, including signed ICFs, pertaining to the conduct of this study must be retained by the investigator for 15 years from the end of the study (as defined in the protocol) unless local regulations or institutional policies require a longer retention period. No records may be destroyed during the retention period without the written approval of the sponsor. No records may be transferred to another location or party without written notification to the sponsor.

A 8 Source Documents

- Source documents provide evidence for the existence of the participant and substantiate the integrity of the data collected. Source documents are filed at the investigator's site.
- Data reported on the eCRF or entered in the eCRF that are transcribed from source documents must be consistent with the source documents or the discrepancies must be explained. The investigator may need to request previous medical records or transfer records, depending on the study. Also, current medical records must be available.
- Definition of what constitutes source data can be found in the clinical study agreement. All information in original records and certified copies of original records of clinical findings, observations, or other activities in a clinical study necessary for the reconstruction and evaluation of the study are defined as source documents. Source data are contained in source documents (original records or certified copies).

A 9 Study and Site Start and Closure

The study start date is the date on which the clinical study will be open for recruitment of participants.

The first act of recruitment is the first site open and will be the study start date.

The sponsor designee reserves the right to close the study site or terminate the study at any time for any reason at the sole discretion of the sponsor. Study sites will be closed upon study completion. A study site is considered closed when all required documents and study supplies have been collected and a study-site closure visit has been performed.

The investigator may initiate study-site closure at any time, provided there is reasonable cause and sufficient notice is given in advance of the intended termination.

Reasons for the early closure of a study site by the sponsor or investigator may include but are not limited to:

- Failure of the investigator to comply with the protocol, the requirements of the IRB/IEC or local health authorities, the sponsor's procedures, or GCP guidelines
- Inadequate recruitment of participants by the investigator
- Discontinuation of further study intervention development

If the study is prematurely terminated or suspended, the sponsor shall promptly inform the investigators, the IECs/IRBs, the regulatory authorities, and any CROs used in the study of the reason for termination or suspension, as specified by the applicable regulatory requirements. The investigator shall promptly inform the participant and should assure appropriate participant therapy and/or follow-up.

Participants from terminated sites will have the opportunity to be transferred to another site to continue the study.

A 10 Publication Policy

- The results of this study may be published or presented at scientific meetings. If this is foreseen, the investigator agrees to submit all manuscripts or abstracts to the sponsor before submission. This allows the sponsor to protect proprietary information and to provide comments.
- The sponsor will comply with the requirements for publication of study results. In accordance with standard editorial and ethical practice, the sponsor will generally support publication of multicentre studies only in their entirety and not as individual site data. In this case, a co-ordinating investigator will be designated by mutual agreement.
- Authorship will be determined by mutual agreement and in line with International Committee of Medical Journal Editors authorship requirements.

Appendix B Adverse Events: Definitions and Procedures for Recording, Evaluating, Follow-up, and Reporting

B 1 Definition of Adverse Events

An AE is the development of any untoward medical occurrence (other than progression of the malignancy under evaluation) in a participant or clinical study participant administered a study intervention and which does not necessarily have a causal relationship with this treatment. An AE can therefore be any unfavourable and unintended sign (eg, an abnormal laboratory finding), symptom (for example nausea, chest pain), or disease temporally associated with the use of a medicinal product, whether or not considered related to the study intervention.

The term AE is used to include both serious and non-serious AEs and can include a deterioration of a pre-existing medical occurrence. An AE may occur at any time, including run-in or washout periods, even if no study intervention has been administered.

B 2 Definitions of Serious Adverse Event

An SAE is an AE occurring during any study phase (ie, run-in, treatment, washout, follow-up), that fulfils one or more of the following criteria:

- Results in death.
- Is immediately life-threatening.
- Requires in-participant hospitalisation or prolongation of existing hospitalisation.
- Results in persistent or significant disability or incapacity.
- Is a congenital abnormality or birth defect.
- Is an important medical event that may jeopardise the participant or may require medical treatment to prevent one of the outcomes listed above.

Adverse events for **malignant tumours** reported during a study should generally be assessed as **Serious** AEs. If no other seriousness criteria apply, the "Important Medical Event" criterion should be used. In certain situations, however, medical judgment on an individual event basis should be applied to clarify that the malignant tumour event should be assessed and reported as a **Non-serious** AE. For example, if the tumour is included as medical history and progression occurs during the study, but the progression does not change treatment and/or prognosis of the malignant tumour, the AE may not fulfil the attributes for being assessed as serious, although reporting of the progression of the malignant tumour as an AE is valid and should occur. Also, some types of malignant tumours, which do not spread remotely after a routine treatment that does not require hospitalisation, may be assessed as Non-serious; examples in adults include Stage 1 basal cell carcinoma and Stage 1A1 cervical cancer

removed via cone biopsy.

The above instruction applies only when the malignant tumour event in question is a new malignant tumour (ie, it is *not* the tumour for which entry into the study is a criterion and that is being treated by the study intervention under study and is not the development of new or progression of existing metastasis to the tumour under study). Malignant tumours that – as part of normal, if rare, progression – undergo transformation (eg, Richter's transformation of B cell chronic lymphocytic leukaemia into diffuse large B cell lymphoma) should not be considered a new malignant tumour.

Life threatening

"Life-threatening" means that the participant was at immediate risk of death from the AE as it occurred or it is suspected that use or continued use of the product would result in the participant's death. "Life-threatening" does not mean that had an AE occurred in a more severe form it might have caused death (eg, hepatitis that resolved without hepatic failure).

Hospitalisation

Outpatient treatment in an emergency room is not in itself a SAE, although the reasons for it may be (eg, bronchospasm, laryngeal oedema). Hospital admissions and/or surgical operations planned before or during a study are not considered AEs if the illness or disease existed before the participant was enrolled in the study, provided that it did not deteriorate in an unexpected way during the study.

Important medical event or medical treatment

Medical and scientific judgment should be exercised in deciding whether a case is serious in situations where important medical events may not be immediately life threatening or result in death, hospitalisation, disability or incapacity but may jeopardise the participant or may require medical treatment to prevent one or more outcomes listed in the definition of serious. These should usually be considered as serious.

Simply stopping the suspect drug does not mean that it is an important medical event; medical judgment must be used.

- Angioedema not severe enough to require intubation but requiring iv hydrocortisone treatment
- Hepatotoxicity caused by paracetamol (acetaminophen) overdose requiring treatment with N-acetylcysteine
- Intensive treatment in an emergency room or at home for allergic bronchospasm
- Blood dyscrasias (eg, neutropenia or anaemia requiring blood transfusion, etc) or convulsions that do not result in hospitalisation

- Development of drug dependency or drug abuse

Intensity rating scale:

The grading scales found in the revised NCI CTCAE latest version (v5.0) will be utilised for all events with an assigned CTCAE grading. For those events without assigned CTCAE grades, the recommendation in the CTCAE criteria that converts mild, moderate and severe events into CTCAE grades should be used. A copy of the CTCAE can be downloaded from the Cancer Therapy Evaluation Program website (http://ctep.cancer.gov). The applicable version of CTCAE should be described clearly.

It is important to distinguish between serious and severe AEs. Severity is a measure of intensity whereas seriousness is defined by the criteria in Appendix B 2. An AE of severe intensity need not necessarily be considered serious. For example, nausea that persists for several hours may be considered severe nausea, but not a SAE unless it meets the criteria shown in Appendix B 2. On the other hand, a stroke that results in only a limited degree of disability may be considered a mild stroke but would be a SAE when it satisfies the criteria shown in Appendix B 2.

B 3 A Guide to Interpreting the Causality Question

When making an assessment of causality consider the following factors when deciding if there is a "reasonable possibility" that an AE may have been caused by the drug.

- Time Course. Exposure to suspect drug. Has the participant actually received the suspect drug? Did the AE occur in a reasonable temporal relationship to the administration of the suspect drug?
- Consistency with known drug profile. Was the AE consistent with the previous knowledge of the suspect drug (pharmacology and toxicology) or drugs of the same pharmacological class? Or could the AE be anticipated from its pharmacological properties?
- De-challenge experience. Did the AE resolve or improve on stopping or reducing the dose of the suspect drug?
- No alternative cause. The AE cannot be reasonably explained by another aetiology such as the underlying disease, other drugs, other host or environmental factors.
- Re-challenge experience. Did the AE reoccur if the suspected drug was reintroduced after having been stopped? AstraZeneca would not normally recommend or support a re-challenge.
- Laboratory tests. A specific laboratory investigation (if performed) has confirmed the relationship.

In difficult cases, other factors could be considered such as:

- Is this a recognised feature of overdose of the drug?
- Is there a known mechanism?

Causality of "related" is made if following a review of the relevant data, there is evidence for a "reasonable possibility" of a causal relationship for the individual case. The expression "reasonable possibility" of a causal relationship is meant to convey, in general, that there are facts (evidence) or arguments to suggest a causal relationship.

The causality assessment is performed based on the available data including enough information to make an informed judgment. With no available facts or arguments to suggest a causal relationship, the event(s) will be assessed as "not related".

Causal relationship in cases where the disease under study has deteriorated due to lack of effect should be classified as no reasonable possibility.

B 4 Medication Error

For the purposes of this clinical study a medication error is an unintended failure or mistake in the treatment process for an AstraZeneca study intervention that either causes harm to the participant or has the potential to cause harm to the participant.

A medication error is not lack of efficacy of the drug, but rather a human or process related failure while the drug is in control of the study site staff or participant.

Medication error includes situations where an error:

- occurred.
- was identified and intercepted before the participant received the drug.
- did not occur, but circumstances were recognised that could have led to an error.

Examples of events to be reported in clinical studies as medication errors:

- Drug name confusion.
- Dispensing error eg, medication prepared incorrectly, even if it was not actually given to the participant.
- Drug not administered as indicated, for example, wrong route or wrong site of administration.
- Drug not taken as indicated eg, tablet dissolved in water when it should be taken as a solid tablet.

- Drug not stored as instructed eg, kept in the fridge when it should be at room temperature.
- Wrong participant received the medication (excluding IRT errors).
- Wrong drug administered to participant (excluding IRT errors).

Examples of events that **do not** require reporting as medication errors in clinical studies:

- Errors related to or resulting from IRT - including those which lead to one of the above listed events that would otherwise have been a medication error.
- Participant accidentally missed drug dose(s) eg, forgot to take medication.
- Accidental overdose (will be captured as an overdose).
- Participant failed to return unused medication or empty packaging.
- Errors related to background and rescue medication, or SoC medication in open-label studies, even if an AstraZeneca product.

Medication errors are not regarded as AEs but AEs may occur as a consequence of the medication error.

Appendix C Handling of Human Biological Samples

C 1 Chain of Custody

A full chain of custody is maintained for all samples throughout their lifecycle.

The investigator at each site keeps full traceability of collected biological samples from the participants while in storage at the centre until shipment or disposal (where appropriate) and records relevant processing information related to the samples whilst at site.

The sample receiver keeps full traceability of the samples while in storage and during use until used or disposed of or until further shipment and keeps record of receipt of arrival and onward shipment or disposal.

AstraZeneca or delegated representatives will keep oversight of the entire life cycle through internal procedures, monitoring of study sites, auditing or process checks, and contractual requirements of external laboratory providers

Samples retained for further use will be stored in the AstraZeneca-assigned biobanks or other sample archive facilities and will be tracked by the appropriate AstraZeneca Team during for the remainder of the sample life cycle.

If required, AstraZeneca will ensure that remaining biological samples are returned to the site according to local regulations or at the end of the retention period, whichever is the sooner.

C 2 Withdrawal of Informed Consent for Donated Biological Samples

If a participant withdraws consent specifically to the subsequent use of donated biological samples, the samples will be disposed of/destroyed/repatriated, and the action documented. If samples are already analysed, AstraZeneca is not obliged to destroy the results of this research. The participant will be presented with the option to opt out of the subsequent use of the donated samples during the withdrawal process. If the participant decides to opt out, then the donated samples will be disposed of or repatriated. If the participant withdraws consent without opting out for the subsequent use of the donated samples, then the samples will be used as per protocol.

Following withdrawal of consent for biological samples, further study participation should be considered in relation to the withdrawal processes outlined in the informed consent.

The investigator:

- Ensures participant's withdrawal of informed consent to the use of donated samples is highlighted immediately to AstraZeneca or delegate.

- Ensures that relevant human biological samples from that participant, if stored at the study site, are immediately identified, disposed of as appropriate, and the action documented.
- Ensures that the participant and AstraZeneca are informed about the sample disposal.

AstraZeneca ensures the organisation(s) holding the samples is/are informed about the withdrawn consent immediately and that samples are disposed of or repatriated as appropriate, and the action documented and study site notified.

C 3 International Airline Transportation Association (IATA) 6.2 Guidance Document

LABELLING AND SHIPMENT OF BIOHAZARD SAMPLES

IATA (https://www.iata.org/whatwedo/cargo/dgr/Pages/download.aspx) classifies infectious substances into 3 categories: Category A, Category B or Exempt

Category A Infectious Substances are infectious substances in a form that, when exposure to it occurs, is capable of causing permanent disability, life-threatening or fatal disease in otherwise healthy humans or animals.

Category A pathogens are, for example, Ebola, Lassa fever virus. Infectious substances meeting these criteria which cause disease in humans or both in humans and animals must be assigned to UN 2814. Infectious substances which cause disease only in animals must be assigned to UN 2900.

Category B Infectious Substances are infectious Substances that do not meet the criteria for inclusion in Category A. Category B pathogens are, for example, Hepatitis A, C, D, and E viruses. They are assigned the following UN number and proper shipping name:

- UN 3373 – Biological Substance, Category B
- are to be packed in accordance with UN3373 and IATA 650

Exempt - Substances which do not contain infectious substances or substances which are unlikely to cause disease in humans or animals are not subject to these Regulations unless they meet the criteria for inclusion in another class.

- Clinical study samples will fall into Category B or exempt under IATA regulations.
- Clinical study samples will routinely be packed and transported at ambient temperature in IATA 650 compliant packaging. (https://www.iata.org/whatwedo/cargo/dgr/Documents/DGR-60-EN-PI650.pdf).

- Biological samples transported in dry-ice require additional dangerous goods specification for the dry-ice content.

Appendix D Actions Required in Cases of Increases in Liver Biochemistry and Evaluation of Hy's Law

D 1 Introduction

This appendix describes the process to be followed in order to identify and appropriately report PHL cases and HL cases. It is not intended to be a comprehensive guide to the management of elevated liver biochemistries.

Specific guidance on managing liver abnormalities can be found in the TMGs (Appendix K Table 23).

During the course of the study the investigator will remain vigilant for increases in liver biochemistry. The investigator is responsible for determining whether a participant meets potential PHL criteria at any point during the study.

All sources of laboratory data are appropriate for the determination of PHL and HL events; this includes samples taken at scheduled study visits and other visits including all local laboratory evaluations even if collected outside of the study visits.

The investigator will also review AE data (eg, for AEs that may indicate elevations in liver biochemistry) for possible PHL events.

The investigator participates, together with AstraZeneca clinical project representatives, in review and assessment of cases meeting PHL criteria to agree whether HL criteria are met. HL criteria are met if there is no alternative explanation for the elevations in liver biochemistry other than DILI caused by the study intervention.

The investigator is responsible for recording data pertaining to PHL/HL cases and for reporting SAEs and AEs according to the outcome of the review and assessment in line with standard safety reporting processes.

D 2 Definitions

Potential Hy's Law Cases

Aspartate aminotransferase or ALT ≥ 3 × ULN **together with** TBL ≥ 2 × ULN at any point during the study following the start of study intervention irrespective of an increase in alkaline phosphatase.

Hy's Law Cases

AST or ALT ≥ 3 × ULN **together with** TBL≥ 2 × ULN, where no other reason, other than the study intervention, can be found to explain the combination of increases, eg, elevated alkaline phosphatase indicating cholestasis, viral hepatitis, another drug.

For PHL and HL the elevation in transaminases must precede or be coincident with (ie, on the same day) the elevation in TBL, but there is no specified timeframe within which the elevations in transaminases and TBL must occur.

D 3 Identification of Potential Hy's Law Cases

In order to identify cases of PHL it is important to perform a comprehensive review of laboratory data for any participant who meets any of the following identification criteria in isolation or in combination:

- ALT $\geq$ 3 × ULN.
- AST $\geq$ 3 × ULN.
- TBL $\geq$ 2 × ULN.

Local laboratories being used

The investigator will, without delay, review each new laboratory report and if the identification criteria are met will:

- Notify the AstraZeneca representative
- Determine whether the participant meets PHL criteria (see Section D 2 Definitions within this Appendix for definition) by reviewing laboratory reports from all previous visits.
- Promptly enter the laboratory data into the laboratory eCRF.

D 4 Follow-up

D 4.1 Potential Hy's Law Criteria Not Met

If the participant does not meet PHL criteria, the investigator will:

- Inform the AstraZeneca representative that the participant has not met PHL criteria.
- Perform follow-up on subsequent laboratory results according to the guidance provided in the CSP.

D 4.2 Potential Hy's Law Criteria Met

If the participant does meet PHL criteria, the investigator will:

- Determine whether PHL criteria were met at any study visit prior to starting study intervention (See Section D 6).
- Notify the AstraZeneca representative who will then inform the central Study Team.

- Within 1 day of PHL criteria being met, the investigator will report the case as an SAE of PHL; serious criterion “Important medical event” and causality assessment “yes/related” according to CSP process for SAE reporting.
- For participants that met PHL criteria prior to starting study intervention, the investigator is not required to submit a PHL SAE unless there is a significant change[#] in the participant’s condition.
- The study physician contacts the investigator, to provide guidance, discuss and agree an approach for the study participants’ follow-up (including any further laboratory testing) and the continuous review of data.
- Subsequent to this contact the investigator will:
  - Monitor the participant until liver biochemistry parameters and appropriate clinical symptoms and signs return to normal or baseline levels, or as long as medically indicated. Completes follow-up SAE Form as required.
  - Investigate the aetiology of the event and perform diagnostic investigations as discussed with the study physician.
  - Complete the 3 Liver eCRF Modules as information becomes available.

#A “significant” change in the participant’s condition refers to a clinically relevant change in any of the individual liver biochemistry parameters (ALT, AST, or TBL) in isolation or in combination, or a clinically relevant change in associated symptoms. The determination of whether there has been a significant change will be at the discretion of the investigator, this may be in consultation with the study physician if there is any uncertainty.

D 5 Review and Assessment of Potential Hy’s Law Cases

The instructions in this section should be followed for all cases where PHL criteria are met.

As soon as possible after the biochemistry abnormality was initially detected, the study physician contacts the investigator in order to review available data and agree on whether there is an alternative explanation for meeting PHL criteria other than DILI caused by the study intervention, to ensure timely analysis and reporting to health authorities within 15 calendar days from date PHL criteria was met. The AstraZeneca Global Clinical Lead or equivalent and Global Safety Physician will also be involved in this review together with other subject matter experts as appropriate.

According to the outcome of the review and assessment, the investigator will follow the instructions below.

Where there is an agreed alternative explanation for the ALT or AST and TBL elevations, a determination of whether the alternative explanation is an AE will be made and subsequently whether the AE meets the criteria for a SAE:

- If the alternative explanation is **not** an AE, record the alternative explanation on the appropriate eCRF.
- If the alternative explanation is an AE/SAE: update the previously submitted PHL SAE and AE eCRFs accordingly with the new information (reassessing event term; causality and seriousness criteria) following the AstraZeneca standard processes.

If it is agreed that there is **no** explanation that would explain the ALT or AST and TBL elevations other than the study intervention:

- Send updated SAE (report term "Hy's Law") according to AstraZeneca standard processes.
    - The "Medically Important" serious criterion should be used if no other serious criteria apply.
    - As there is no alternative explanation for the HL case, a causality assessment of "related" should be assigned.

If, there is an unavoidable delay, of over 15 calendar days in obtaining the information necessary to assess whether or not the case meets the criteria for HL, then it is assumed that there is no alternative explanation until such time as an informed decision can be made:

- Provides any further update to the previously submitted SAE of PHL, (report term now "Hy's Law case") ensuring causality assessment is related to study intervention and seriousness criterion is medically important, according to CSP process for SAE reporting.
- Continue follow-up and review according to agreed plan. Once the necessary supplementary information is obtained, repeat the review and assessment to determine whether HL criteria are still met. Update the previously submitted PHL SAE report following CSP process for SAE reporting, according to the outcome of the review and amending the reported term if an alternative explanation for the liver biochemistry elevations is determined.

D 6 Actions Required When Potential Hy's Law Criteria are Met Before and After Starting Study Intervention

This section is only applicable to participants with liver metastases who meet PHL criteria on study intervention, having previously met PHL criteria at a study visit prior to starting study intervention.

At the first on-study intervention occurrence of PHL criteria being met, the investigator will determine if there has been a **significant change** in the participant's condition compared with the last visit where PHL criteria were met.

- If there is no significant change no action is required.
- If there is a significant change, notify the AstraZeneca representative, who will inform the central Study Team, then follow the subsequent process described in Section D 4.2.

D 7 Actions Required for Repeat Episodes of Potential Hy's Law

This section is applicable when a participant meets PHL criteria on study intervention and has already met PHL criteria at a previous on study intervention visit.

The requirement to conduct follow-up, review and assessment of a repeat occurrence(s) of PHL is based on the nature of the alternative cause identified for the previous occurrence.

The investigator should determine the cause for the previous occurrence of PHL criteria being met and answer the following question:

Was the alternative cause for the previous occurrence of PHL criteria being met found to be the disease under study eg, chronic or progressing malignant disease, severe infection or liver disease or did the participant meet PHL criteria prior to starting study intervention and at their first on-study intervention visit, as described in Section D 6 of this Appendix ?

If **No**: follow the process described in Section D 4.2 for reporting PHL as an SAE.

If **Yes**: Determine if there has been a significant change in the participant's condition compared with when PHL criteria were previously met.

- If there is no significant change no action is required.
- If there is a significant change follow the process described in Section D 4.2 for reporting PHL as an SAE.

D 8 Laboratory Tests

The laboratory tests listed in Table 13 may be performed for central laboratories. In this study, a local laboratory will be used for safety tests and the Hy's Law Laboratory Kit is not applicable for this study.

Table 13 Hy's Law Laboratory Kit for Central Laboratories

Additional standard chemistry and coagulation tests	GGT<br>LDH<br>Prothrombin time<br>INR
Viral hepatitis	IgM anti-HAV<br>IgM and IgG anti-HBc<br>HBsAg<br>HCV DNA [a]<br>IgG anti-HCV<br>HCV RNA [a]<br>IgM anti-HEV<br>HEV RNA
Other viral infections	IgM and IgG anti-CMV<br>IgM and IgG anti-HSV<br>IgM and IgG anti-EBV
Autoimmune hepatitis	Antinuclear antibody<br>Anti-liver/kidney microsomal antibody<br>Anti-smooth muscle antibody
Metabolic diseases	Alpha-1-antitrypsin<br>Ceruloplasmin<br>Iron<br>Ferritin<br>Transferrin [b]<br>Transferrin saturation

[a] HCV RNA; HCV DNA are only tested when IgG anti-HCV is positive or inconclusive.
CMV = cytomegalovirus; DNA = deoxyribonucleic acid; EBV = Epstein-Barr virus; GGT = gamma glutamyl transferase; HAV = hepatitis A virus; HBc = hepatitis B core antigen; HBsAg = hepatitis B surface antigen; HCV = hepatitis C virus; HEV = hepatitis E virus; HSV = herpes simplex virus; IgG = immuno-globulin G; IgM = immunoglobulin M; INR = international normalised ratio; LDH = lactate dehydrogenase; RNA = ribonucleic acid.

D 9 References

Aithal et al 2011
Aithal GP, Watkins PB, Andrade RJ, Larrey D, Molokhia M, Takikawa H, et al. Case definition and phenotype standardization in drug-induced liver injury. Clin Pharmacol Ther. 2011;89(6):806-15.

FDA Guidance 2009
Food and Drug Administration. Guidance for industry: Drug-induced liver injury: premarketing clinical evaluation. July 2009. Available from: URL: https://www.fda.gov/downloads/guidances/UCM174090.pdf. Accessed 08 October 2019.

Appendix E Guidelines for Evaluation of Objective Tumour Response Using RECIST 1.1 Criteria (Response Evaluation Criteria in Solid Tumours)

Introduction

This appendix details the implementation of RECIST 1.1 guidelines (Eisenhauer et al 2009). Investigator assessments will use the RECIST 1.1 guidelines described in this appendix.

Imaging modalities and acquisition specifications for RECIST 1.1

A summary of the imaging modalities that can be used for tumour assessment of TLs, NTLs and NLs is provided in Table 14.

Table 14 Summary of Imaging Modalities for Tumour Assessment

Target Lesions	Non-target Lesions	New Lesions
CT<br>MRI	CT<br>MRI<br>Plain X-ray<br>Chest X-ray	CT<br>MRI<br>Plain X-ray<br>Chest X-ray<br>Bone scan (Scintigraphy)<br>^{18}F-fluoro-deoxyglucose-PET/CT

CT = computed tomography; PET/CT = positron emission tomography/CT; MRI = magnetic resonance imaging.

Computed Tomography and Magnetic Resonance Imaging

CT with IV contrast is the preferred imaging modality (although MRI with IV contrast is acceptable if CT is contraindicated) to generate reproducible anatomical images for tumour assessments (ie, for measurement of TLs, assessment of NTLs, and identification of NLs). It is essential that the same correct imaging modality, image acquisition parameters (eg, anatomic coverage, imaging sequences, etc), imaging facility, tumour assessor (eg, radiologist), and method of tumour assessment (eg, RECIST 1.1) are used consistently for each participant throughout the study. The use of the same scanner for serial scans is recommended, if possible. It is important to follow the image collection/tumour assessment schedule as closely as possible (refer to the SoA, Section 1.3), and this on-study imaging schedule MUST be followed regardless of any delays in dosing or missed imaging visits. If an unscheduled assessment is performed (eg, to investigate clinical signs/symptoms of progression) and the participant has not progressed, every attempt should be made to perform the subsequent scan acquisitions at the next scheduled imaging visit.

Due to its inherent rapid acquisition (seconds), CT is the imaging modality of choice. Body scans should be performed with breath-hold scanning techniques, if possible. Therefore, CT of the chest is recommended over MRI due to significant motion artefacts (eg, heart, major blood

vessels, breathing) associated with MRI. MRI has excellent contrast and spatial and temporal resolutions; however, there are many image acquisition variables involved in MRI, which greatly impact image quality, lesion conspicuity, and measurement. Furthermore, the availability of MRI is variable globally. The modality used at follow-up should be the same as was used at baseline, and the lesions should be measured/assessed on the same pulse sequence. In general, local oncology diagnostic imaging parameters are applied for scan acquisition. It is beyond the scope of this appendix to prescribe specific MRI pulse sequence parameters for all scanners, body parts, and diseases.

The most critical CT and MRI image acquisition parameters for optimal tumour evaluation are anatomic coverage, contrast administration, slice thickness, and reconstruction interval.

a. Anatomic coverage: Optimal anatomic coverage for most solid tumours is the chest-abdomen (-pelvis). Coverage should encompass all areas of known predilection for metastases in the disease under evaluation and should additionally investigate areas that may be involved based on signs and symptoms of individual participants. Because a lesion later identified in a body part not scanned at baseline would be considered as a NL representing PD, careful consideration should be given to the extent of imaging coverage at baseline and at subsequent follow-up time points. This will enable better consistency not only of tumour measurements but also identification of new disease.

Required anatomical regions to be imaged for assessment of tumour burden (TLs and/or NTLs) at baseline and follow-up visits vary according to the study, and these time points are specified in the SoA. Examples include the following:

- IV contrast-enhanced CT of chest-abdomen (including the entire liver and both adrenal glands) (-pelvis).
- Non-contrast CT of chest and IV contrast-enhanced abdomen (including the entire liver and both adrenal glands) (-pelvis).
- IV contrast-enhanced CT or MRI of the head and neck.
- IV contrast-enhanced MRI (preferred) or CT of the brain.

For chest-abdomen (-pelvis) imaging, the following are scanning options in decreasing order of preference, with additional options (2 to 4) for consideration when participants have sensitivity to IV contrast or have compromised renal function:

1. Chest-abdomen (-pelvis) CT with IV CT contrast (most preferred).
2. Chest CT without IV-contrast + abdomen (-pelvis) MRI with IV MRI contrast, if CT IV contrast (iodine based) is medically contraindicated at any time during the study.

3. Chest-abdomen (-pelvis) CT without IV contrast, if both IV CT and MRI contrast are medically contraindicated or the participant has compromised renal function.
4. Chest-abdomen (-pelvis) MRI with IV MRI contrast, if CT cannot be performed at any time during the study.

b. IV contrast administration: Optimal visualisation and measurement of metastases in solid tumours require consistent administration (dose and rate) of IV contrast as well as timing of scanning. An adequate volume of a suitable contrast agent should be given so that the tumour lesions are demonstrated to best effect and a consistent method is used on subsequent examinations for any given participant. Oral contrast is recommended to help visualise and differentiate structures in the abdomen and pelvis.

c. Slice thickness and reconstruction interval: It is recommended that CT or MRI scans be acquired/reconstructed as contiguous (no gap) slices with ≤ 5 mm thickness throughout the entire anatomic region of interest for optimal lesion measurements. Exceptionally, particular institutions may perform medically acceptable scans at slice thicknesses > 5 mm. If this occurs, the minimum size of measurable lesions at baseline should be twice the slice thickness of the baseline scans.

For CT scans, all window settings should be included in the assessment, particularly in the thorax where lung and soft tissue windows should be considered. When measuring lesions, the TL should be measured on the same window setting for repeated examinations throughout the study.

Chest X-ray

Chest X-ray assessment will not be used for the assessment of TLs. Chest X-ray can, however, be used to assess NTLs and to identify the presence of NLs. However, there is preference that a higher resolution modality, such as CT, be used to confirm the presence of NLs.

Plain X-ray

Plain X-ray may be used as a method of assessment for bone NTLs and to identify the presence of new bone lesions.

Isotopic bone scan

Bone lesions identified on an isotopic bone scan at baseline and confirmed by CT, MRI, or X-ray at baseline should be recorded as NTLs and followed by the same method per baseline assessment (CT, MRI, or X-ray).

Isotopic bone scans may be used as a method of assessment to identify the presence of new bone lesions at follow-up visits. NLs may be recorded in case positive hot-spots appear on a bone scan that were not present on a previous bone scan; however, a newly observed

equivocal hot-spot on a bone scan that cannot be verified with correlative imaging (CT, MRI, or X-ray) of the same anatomical region shall not be the only trigger for a PD assessment at that time point.

^{18}F-Fluoro-deoxyglucose-PET/CT

^{18}F-fluoro-deoxyglucose positron emission tomography (PET)/CT scans may be used as a method for identifying new extrahepatic lesions (but not intrahepatic lesions) for RECIST 1.1 assessments according to the following algorithm: NLs will be recorded where there is positive ^{18}F-Fluoro-deoxyglucose uptake[1] not present on baseline or prior ^{18}F-fluoro-deoxyglucose-PET scan or in a location corresponding to a NL on a companion CT/MRI collected close in time to the ^{18}F-fluoro-deoxyglucose-PET scan. The PET portion of the PET/CT introduces additional data that may bias an investigator if it is not routinely or serially performed. Therefore, if there is no baseline or prior ^{18}F-fluoro-deoxyglucose-PET scan available for comparison, and no evidence of NLs on companion CT/MRI scans, then follow-up CT/MRI assessments should continue as per the regular imaging schedule to verify the unequivocal presence of NLs.

At present, low-dose or attenuation correction CT portions of a combined ^{18}F-fluoro-deoxyglucose-PET/CT scan are of limited use in anatomically based efficacy assessments, and it is therefore suggested that they should not substitute for dedicated diagnostic contrast-enhanced CT scans for tumour measurements by RECIST 1.1. In exceptional situations, if a site can document that the CT performed, as part of a PET/CT examination, is of identical diagnostic quality (with IV contrast) to a dedicated diagnostic CT scan, then the CT portion of the PET/CT can be used for RECIST 1.1 tumour assessments. Caution that this is not recommended because the PET portion of the CT introduces additional (PET) data that may bias an investigator if it is not routinely or serially performed.

Ultrasound

Ultrasound examination will not be used for RECIST 1.1 assessment of tumours as it is not a reproducible acquisition method (operator dependent), is subjective in interpretation, and may not provide an accurate assessment of the true tumour size. Tumours identified by ultrasound will need to be assessed by correlative CT or MRI anatomical scan.

Other tumour assessments

Clinical examination

Clinical examination of skin/surface lesions (by visual inspection or manual palpation) will not be used for RECIST 1.1 assessments. Tumours identified by clinical examination will

1 A positive ^{18}F-fluoro-deoxyglucose-PET scan lesion should be reported only when an uptake (eg, standard uptake value) greater than twice that of the surrounding tissue or liver is observed.

need to be assessed by correlative CT or MRI anatomical scans.

Endoscopy and laparoscopy

Endoscopy and laparoscopy will not be used for tumour assessments as they are not validated in the context of tumour assessment.

Histology and cytology

Histology or tumour markers on tumour biopsy samples will not be used as part of the tumour response assessment as per RECIST 1.1.

Results of cytological examination for the neoplastic origin of any effusion (eg, ascites, pericardial effusion, and pleural effusion) that appears or worsens during the study will not be used as part of the tumour response assessment as per RECIST 1.1.

Furthermore, an overall assessment of CR (all other disease disappears/reverts to normal) would be changed to PR if an effusion remains present radiologically.

Measurability of tumour lesions at baseline

RECIST 1.1 measurable lesions at baseline

A tumour lesion that can be accurately measured at baseline as ≥ 10 mm in the longest diameter for non-nodal lesions or ≥ 15 mm in short axis[2] diameter for lymph node lesions with IV contrast-enhanced CT or MRI and that is suitable for accurate repeated measurements. Please see additional RECIST 1.1 guidance below on measurability of intrahepatic hepatocellular carcinoma lesions and porta hepatis lymph nodes.

Non-measurable lesions at baseline

- Truly non-measurable lesions include the following:
  - Bone lesions (see exception below for soft tissue component).
  - Leptomeningeal disease.
  - Ascites, pleural effusion, or pericardial effusion.
  - Inflammatory breast disease.
  - Lymphangitic involvement of skin or lung.
- All other lesions, including small lesions (longest diameter < 10 mm or pathological lymph nodes with ≥ 10 mm to < 15 mm short axis diameter at baseline).[3]

2 The short axis is defined as the longest in-plane axis perpendicular to the long axis.
3 Lymph nodes with < 10 mm short axis diameter are considered non-pathological and should not be recorded or followed as NTLs.

- Previously irradiated lesions.[4] However, a previously irradiated lesion that has shown objective progression and meets the other requirements for a measureable lesion may be considered as a TL if it is the only lesion available.
- Brain metastasis.

Special considerations regarding lesion measurability at baseline

- Bone lesions:
  - Bone scan, PET scan, or plain X-ray are not considered adequate imaging techniques to measure bone lesions; however, these techniques can be used to confirm the presence or disappearance of bone lesions.
  - Lytic bone lesions or mixed lytic-blastic lesions, with identifiable soft tissue components, can be considered measurable if the soft tissue component meets the definition of measurability.
  - Blastic lesions are considered non-measurable.
- Cystic lesions thought to represent cystic metastases can be considered measurable lesions if they meet the criteria for measurability from a radiological point of view, but if non-cystic lesions are present in the same participant, these should be selected over cystic lesions as TLs.

RECIST 1.1 TL selection at baseline

A maximum of 5 measurable lesions, with a maximum of 2 lesions per organ (including lymph nodes collectively considered as a single organ), representative of all lesions involved should be identified as TLs at baseline. TLs should be selected on the basis of their size (longest diameter for non-nodal lesions or short axis diameter for nodal lesions), but in addition should be those that lend themselves to reproducible repeated measurements. It may be the case that, on occasion, the largest lesion does not lend itself to reproducible measurement, in which circumstance the next largest lesion that can be measured reproducibly should be selected.

Lymph nodes, in any location (local/regional and distant), are collectively considered as a single organ, with a maximum of 2 lymph nodes as TLs. A bilateral organ (eg, adrenal glands), a segmented organ (eg, liver), or a multilobed organ (eg, lung) is each considered as a single organ.

The site and location of each TL should be documented, as well as the longest axis diameter for non-nodal lesions (or short axis diameter for lymph nodes). All measurements should be

---

4 Localised post-radiation changes that affect lesion size may occur. Therefore, lesions that have been previously irradiated are typically considered non-measurable and as NTL at baseline and followed up as part of the NTL assessment.

recorded in millimetres. At baseline, the sum of the diameters for all TLs will be calculated and reported as the baseline sum of diameters. At follow-up visits, the sum of diameters for all TLs will be calculated and reported as the follow-up sum of diameters.

Special cases for TL assessment at baseline

- For TLs measurable in 2 or 3 dimensions, always report the longest diameter. For pathological lymph nodes measurable in 2 or 3 dimensions, always report the short axis diameter.
- When lymph nodes are coalesced and no longer separable in a conglomerate mass, the vector of the longest diameter should be used to determine the perpendicular vector for the maximal short axis diameter of the coalesced mass. Non-nodal lesions that coalesce should similarly be assessed by the longest axis diameter.
- Tumour lesions selected for newly acquired screening biopsy should not be selected as TLs, unless imaging occurred at least approximately 2 weeks after biopsy, allowing time for healing.
- If the CT/MRI slice thickness used is > 5 mm, the minimum size of measurable disease at baseline should be twice the slice thickness of the baseline scan.
- If a lesion has completely disappeared, the diameter should be recorded as 0 mm. If a lesion appears in the same location on a subsequent scan, it will be recorded as a NL.

RECIST 1.1 NTL selection at baseline

All other lesions, including non-measurable lesions and surplus measurable lesions, not recorded as TLs should be identified as NTLs at baseline. Measurements of these lesions are not required, but the presence or absence of each should be noted throughout follow-up.

Evaluation of tumour response and progression

RECIST 1.1 TL assessment at follow-up

This section defines the criteria used to determine objective tumour visit response for RECIST 1.1-defined TLs. The imaging modality, location, and scan date of each TL identified previously at baseline should be documented at follow-up visits with the long axis diameter for non-nodal lesions or short axis diameter for lymph node lesions. All measurements should be recorded in millimetres. The sum of the diameters for all TLs at each follow-up visit will be compared with the baseline sum of diameters (for response or SD) or to the smallest prior (nadir) sum of diameters (for progression) (Table 15).

Special cases for TL assessment at follow-up:

- If a lesion has completely disappeared, the diameter should be recorded as 0 mm. If a lesion appears in the same location on a subsequent scan, it will be recorded as an NL.

- If a TL splits into 2 or more parts, the sum of the diameters of those parts should be recorded.
- If 2 or more TLs merge, then the sum of the diameters of the combined lesion should be recorded for 1 of the lesions and 0 mm recorded for the other lesion(s). If the merged TLs are non-nodal lesions, record the long axis diameter of the merged lesion. If pathologic lymph nodes coalesce and are no longer individually separable within a conglomerate mass, the vector of the longest diameter of the coalesced mass should be used to determine the perpendicular vector for the maximal short axis diameter.
- If a TL is believed to be present and is faintly seen but too small to measure, a default value of 5 mm should be assigned. If an accurate measure can be given, this should be recorded, even if it is below 5 mm.
- If a TL cannot be measured accurately due to it being too large, provide an estimate of the size of the lesion.
- When a TL has had any intervention (eg, definitive radiotherapy, embolisation, surgery, transarterial chemoembolisation, etc) during the study, the size of the TL should still be provided where possible and the intervention recorded in the RECIST 1.1 eCRF for the current imaging visit and all subsequent visits. If a TL has been completely removed (surgery) or disappears, the longest diameter should be recorded as 0 mm.

Table 15 RECIST 1.1 Evaluation of Target Lesions

CR	Disappearance of all TLs since baseline. Any pathological lymph nodes selected as TLs must have a reduction in short axis diameter to <10 mm.
PR	At least a 30% decrease in the sum of the diameters of TL, taking as reference the baseline sum of diameters.
SD	Neither sufficient decrease in the sum of diameters to qualify for PR nor sufficient increase to qualify for PD.
PD	At least a 20% increase in the sum of diameters of TLs, taking as reference the smallest previous sum of diameters (nadir). This includes the baseline sum if that is the smallest on study. In addition to the relative increase of 20%, the sum must demonstrate an absolute increase of at least 5 mm from nadir.
NE	Only relevant if any of the TLs at follow-up were not assessed or NE (eg, missing anatomy) or had a lesion intervention at this visit. Note: If the sum of diameters meets the PD criteria, PD overrides NE as a TL response.
Not applicable	Only relevant if no TLs present at baseline.

CR = complete response; NE = not evaluable; PD = progression of disease; PR = partial response; SD = stable disease; TL = target lesion.

RECIST 1.1 NTL assessment at follow-up

All other lesions (or sites of disease) not recorded as TLs should be identified as NTLs at

baseline. Measurements are not required for these lesions, but their status should be followed at subsequent visits. At each visit, an overall assessment of the NTL response should be recorded by the investigator (Table 16).

To achieve "unequivocal progression" on the basis of NTLs, there must be an overall level of substantial worsening in non-target disease such that, even in presence of SD or PR in TLs, the overall tumour burden has increased sufficiently to merit unequivocal progression by NTLs. A modest "increase" in the size of 1 or more NTLs is usually not sufficient to qualify for unequivocal progression status. The designation of overall progression solely on the basis of change in non-target disease in the face of SD or PD of target disease will therefore be extremely rare.

Table 16 RECIST 1.1 Evaluation of Non-target Lesions

CR	Disappearance of all NTLs since baseline. All lymph nodes must be non-pathological in size (<10 mm short axis).
Non-CR/non-PD	Persistence of 1 or more NTLs.
PD	Unequivocal progression of existing NTLs. Unequivocal progression may be due to an important progression in 1 lesion only or in several lesions. In all cases, the progression MUST be clinically significant for the physician to consider changing (or stopping) therapy.
NE	Only relevant when 1 or some of the NTLs were not assessed and, in the investigator's opinion, they are not able to provide an evaluable overall NTL assessment at this visit.<br>Note: For participants without TLs at baseline, this is relevant if any of the NTLs were not assessed at this visit and the progression criteria have not been met.
Not applicable	Only relevant if no NTLs present at baseline.

CR = complete response; NE = not evaluable; NTL = non-target lesion; PD = progression of disease; TL = target lesion.

<u>RECIST 1.1 NL identification at follow-up</u>

Details, including the imaging modality, the date of scan, and the location of any NLs will also be recorded in the eCRF. The presence of 1 or more NLs is assessed as progression. The finding of a NL should be unequivocal, ie, not attributable to differences in scanning technique, change in imaging modality, or findings thought to represent something other than tumour. If a NL is equivocal, for example because of its small size, the treatment and tumour assessments should be continued until the previously (pre-existing) NL has been assessed as unequivocal at a follow-up visit, and then the progression date should be declared using the date of the initial scan when the NL first appeared.

A lesion identified at a follow-up assessment in an anatomical location that was not scanned at baseline is considered a NL and will indicate PD.

RECIST 1.1 evaluation of overall visit response at follow-up

Derivation of overall visit response as a result of the combined assessment of TLs, NTLs, and NLs uses the algorithm shown in Table 17.

Table 17 RECIST 1.1 Overall Visit Response

Target Lesions	Non-Target Lesions	New Lesions	Overall Visit Response
CR	CR	No	CR
CR	NA	No	CR
CR	Non-CR/Non-PD	No	PR
CR	NE	No	PR
PR	Non-PD or NE or NA	No	PR
SD	Non-PD or NE or NA	No	SD
NE	Non-PD or NE	No	NE
PD	Any	Yes or No	PD
Any	PD	Yes or No	PD
Any	Any	Yes	PD

Non-CR/Non-PD for overall response if only NTL (no TLs) are present at baseline.
Note: An overall assessment of CR (all other disease disappears/reverts to normal) would be changed to PR if ascites remains present radiologically.
CR = complete response; NA = not applicable (only relevant if there were no TLs or NTLs at baseline), NE = not evaluable; NTL = non-target lesion; PD = progression of disease; PR = partial response; SD = stable disease; TL = target lesion.

The following overall visit responses are possible depending on the extent of tumour disease at baseline:

- For participants with TLs (at baseline): CR, PR, SD, PD, or NE.
- For participants with NTLs only (at baseline): CR, Non-CR/Non-PD, PD, or NE.
- For participants with no disease at baseline: no evidence of disease (available as an option in the eCRF), PD, or NE.

Central imaging

Images, including unscheduled visit scans, will be collected on an ongoing basis and sent to an AstraZeneca-appointed iCRO for quality control, storage, and for ICR. Digital copies of all original scans should be stored at the investigator site as source documents. Electronic image transfer from the sites to the iCRO is strongly encouraged. An ICR of images will be performed at the discretion of AstraZeneca. Results of these independent reviews will not be communicated to investigators, and results of investigator tumour assessments will not be shared with the central reviewers.

The management of participants will be based in part upon the results of the tumour assessments conducted by the investigator. Further details of the ICR will be documented in an Independent Review Charter.

References

Eisenhauer et al 2009
Eisenhauer EA, Therasse P, Bogaerts J, Schwartz LH, Sargent D, Ford R, et al. New response evaluation criteria in solid tumours: revised RECIST guideline (version 1.1). Eur J Cancer. 2009;45(2):228-47.

Appendix F Contraception Requirements

Contraception requirements for this study are as follows.

F 1 Female Participants

Women not of childbearing potential are defined as those who are surgically sterile (ie, bilateral salpingectomy, bilateral oophorectomy, or complete hysterectomy) or who are post-menopausal.

Women will be considered post-menopausal if they have been amenorrhoeic for 12 months without an alternative medical cause.

Females on HRT and whose menopausal status is in doubt will be required to use one of the contraception methods outlined for WOCBP if they wish to continue their HRT during the study. Otherwise, they must discontinue HRT to allow confirmation of post-menopausal status prior to study enrolment. For most forms of HRT, at least 2-4 weeks will elapse between the cessation of therapy and the blood draw; this interval depends on the type and dosage of HRT. Following confirmation of their post-menopausal status, participants can resume use of HRT during the study without use of a contraceptive method.

WOCBP who are not totally sexually abstinent (ie, refraining from heterosexual intercourse during the entire period of risk associated with study interventions) and intend to be sexually active with a non-sterilised male partner must use at least 1 highly effective method of contraception (Table 18). They should have been stable on their chosen method of birth control for a minimum of 3 months before entering the study and continue to use it throughout the total duration of the drug treatment and the drug washout period (7 months after the last dose of study intervention).

Non-sterilised male partners of a WOCBP must use a male condom plus spermicide (condom alone in countries where spermicides are not approved) throughout this period. Cessation of birth control after this point should be discussed with a responsible physician.

Periodic abstinence, the rhythm method, and the withdrawal method are not acceptable methods of contraception.

Total sexual abstinence is an acceptable method provided it is the usual lifestyle of the participant. The reliability of sexual abstinence needs to be evaluated in relation to the duration of the study.

Female participants should refrain from breast-feeding throughout this period.

Women must not donate, or retrieve for their own use, ova from the time of screening and throughout the study treatment period, and for at least 7 months after the final study

intervention administration. Female patients may wish to consider preservation of ova prior to enrolment in the study.

F 2 Male Participants with a Female Partner of Childbearing Potential

Non-sterilised male participants (including males sterilised by a method other than bilateral orchidectomy, eg, vasectomy) who intend to be sexually active with a female partner of childbearing potential must be using an acceptable method of contraception such as male condom plus spermicide (condom alone in countries where spermicides are not approved) from the time of screening throughout the total duration of the study and the drug washout period (4 months after the last dose of study intervention) to prevent pregnancy in a partner.

Not engaging in sexual activity for the duration of the study and drug washout period is an acceptable practice. The reliability of sexual abstinence needs to be evaluated in relation to the duration of the study and the preferred and usual lifestyle of the participant.

Periodic abstinence, the rhythm method, and the withdrawal method are not acceptable methods of contraception.

Male participants should refrain from sperm donation or banking throughout this period. Consideration of preservation of sperm should be made prior to entering the study.

Vasectomised males are considered fertile and should still use a male condom plus spermicide as indicated above during the clinical study.

Even if the female partner is pregnant, male participants should still use a condom plus spermicide (where approved), as indicated above during the clinical study, if there is a concern about damaging the developing foetus from drug in ejaculate.

Female partners (of childbearing potential) of male participants must also use a highly effective method of contraception throughout this period (Table 18).

F 3 Highly Effective Methods of Contraception

Highly effective methods of contraception, defined as those that result in a low failure rate (ie, less than 1% per year) when used consistently and correctly, are described in Table 18. Note that some contraception methods are not considered highly effective (eg, male or female condom with or without spermicide; female cap, diaphragm, or sponge with or without spermicide; non-copper containing intrauterine device; progestogen-only oral hormonal contraceptive pills where inhibition of ovulation is not the primary mode of action [excluding Cerazette/desogestrel which is considered highly effective]; and triphasic combined oral contraceptive pills).

Table 18 Highly Effective Methods of Contraception (< 1% Failure Rate)

Non-hormonal Methods	Hormonal Methods
• Total heterosexual abstinence (evaluate in relation to the duration of the clinical study and the preferred and usual lifestyle choice of the participant)<br>• Vasectomised sexual partner (provided that partner is the sole sexual partner of the trial participant and that the vasectomised partner has received medical assessment of the surgical success)<br>• Bilateral tubal occlusion<br>• Intrauterine device (provided coils are copper-banded)	• Combined (oestrogen and progestogen containing) hormonal contraception associated with inhibition of ovulation<br>◦ oral<br>◦ intravaginal<br>◦ transdermal<br>• Progestogen-only hormonal contraception associated with inhibition of ovulation<br>◦ oral<br>◦ injectable<br>◦ implantable<br>• Intrauterine hormone-releasing system (IUS)

Appendix G Concomitant Medications

G 1 Restricted, Prohibited, and Permitted Concomitant Medications/Therapies

Restricted, prohibited, and permitted concomitant medications/therapies are described in Table 19, Table 20, and Table 21, respectively. Refer also to the dose modification guidelines for management of study intervention-related toxicities in Section 6.6. Participants must be instructed not to take any medications, including over-the-counter products, without first consulting with the investigator.

Table 19 Restricted Medications/Therapies

Medication/class of drug/therapy	Usage (including limits for duration permitted and special situations in which it is allowed)
Tobacco products, e-cigarettes and vaping	Use of tobacco products, e-cigarettes and vaping is strongly discouraged but not prohibited.<br>Any prior or current use of these products should be recorded in the eCRF.
Haematopoietic growth factors	May be used for prophylaxis or treatment based on the clinical judgment of the investigator.
Dietary supplements, medications not prescribed by the investigator, and alternative/complementary treatments	Concomitant use is discouraged, but not prohibited.
Prophylactic or supportive treatment of study-drug induced AEs	As per investigator's discretion and institutional guidelines
Hormonal therapy	For non-cancer-related conditions (eg, HRT) only
Corticosteroids and/or bisphosphonates	For the treatment of bone metastases and for the treatment of specific adverse drug reactions (refer to TMG, Table 23)<br>See Table 20 for restrictions on corticosteroids when used as immunosuppressants

AEs = adverse events; eCRF = electronic Case Report Form; HRT = hormone replacement therapy; TMG = toxicity management guidelines.

With the exception of the study intervention, T-DXd, the medications in Table 20 are considered exclusionary during the study. The sponsor must be notified if a participant receives any of these during the study.

Table 20 Prohibited Medications/Therapies

Prohibited medication/class of drug/therapy	Usage
Chloroquine or hydroxychloroquine	Concomitant treatment with chloroquine or hydroxychloroquine is not allowed during the study treatment. If treatment with chloroquine or hydroxychloroquine treatment is absolutely required for COVID-19, study intervention must be interrupted. If chloroquine or hydroxychloroquine is administered, a washout period of at least 14 days is required before restarting study intervention (see Appendix H for further details).
Any concurrent chemotherapy, anticancer study intervention or biological, radiotherapy (except palliative radiotherapy to areas other than chest, after consultation with the sponsor study physician) or hormonal therapy for cancer treatment.	Must not be given concomitantly while the participant is on study intervention.<br>Concurrent use of hormones for noncancer-related conditions (eg, insulin for diabetes and hormone replacement therapy) is acceptable.
Immunosuppressive medications, including corticosteroids	T-DXd cannot be administered when the participant is taking immunosuppressive medications, including corticosteroids with the exception of:<br>• short-term courses (< 2 weeks) of low to moderate dose (<10mg prednisolone per day or equivalent)<br>• long-term, alternate-day treatment with short-acting preparations; maintenance physiological doses (replacement therapy)<br>• administered topically (skin or eyes), by aerosol, or by intra-articular, bursal, or tendon injection.<br>Treatment with corticosteroids to prevent or treat hypersensitivity reactions to radiographic contrast agents is allowed.<br>A temporary period of steroid treatment will be allowed for different indications after discussion with the sponsor study physician (eg, chronic obstructive pulmonary disease, radiation, nausea, etc).<br>Participants with bronchopulmonary disorders who require intermittent use of bronchodilators (such as albuterol) will not be excluded from this study.<br>Use of immunosuppressive medications for the management of study intervention-related AEs or in participants with contrast allergies is acceptable.<br>Immunosuppressive medications also include drugs like methotrexate, azathioprine, and tumour necrosis factor-alpha blockers.

Table 20 Prohibited Medications/Therapies

Prohibited medication/class of drug/therapy	Usage
Live vaccine	Participants, if assigned study intervention, should not receive live vaccine during the study and up to 30 days after the last dose of study intervention. Participants who have received live, attenuated vaccine within 30 days prior to the first dose of T-DXd will be excluded
Herbal and natural remedies that may interfere with interpretation of study results	Must not be given concomitantly unless agreed by the sponsor

AE = adverse event; COVID-19 = coronavirus 2019-nCoV; T-DXd = trastuzumab deruxtecan.

Table 21 Supportive Medications/Therapies

Supportive medication/class of drug/therapy	Usage
Prophylactic anti-emetic agents	Recommended that participants receive prophylactic anti-emetic agents prior to infusion of T-DXd and on subsequent days.<br>Antiemetics such as 5-HT3 receptor antagonists or NK1 receptor antagonists and/or steroids (eg, dexamethasone) should be considered and administered in accordance with the prescribing information or institutional guidelines.
Concomitant medications or treatments (eg, acetaminophen or diphenhydramine) deemed necessary to provide adequate AE management, except for those medications identified as "prohibited," as listed above	To be administered as prescribed by the investigator except for those medications identified as "prohibited," as listed in Table 20
Best supportive care (including antibiotics, nutritional support, correction of metabolic disorders, optimal symptom control, and pain management [including palliative radiotherapy, etc]) except for those medications identified as "prohibited," as listed above	Should be used, when necessary, for all participants except for those medications identified as "prohibited," as listed in Table 20
Corticosteroids and/or bisphosphonates for the treatment of bone metastases and for the treatment of specific adverse drug reactions (refer to TMG, Table 23)	Permitted
Inactivated viruses, such as those in the influenza vaccine	Permitted
Required for management of other medical conditions	As required except for those identified as "prohibited," as listed in Table 20

5-HT3 = 5-hydroxytryptamine 3; AE = adverse event; NK1 = neurokinin 1; T-DXd = trastuzumab deruxtecan; TMG = toxicity management guidelines.

Appendix H Instructions Related to Severe Acute Respiratory Syndrome Coronavirus 2 (COVID-19)

Benefit Risk Considerations for COVID-19

The emergence of the coronavirus 2019-nCoV (SARS-CoV-2 / COVID-19) presents a potential safety risk for patients. Several risk mitigation factors have been implemented in this study. Notably, the eligibility criteria will exclude participants with COVID-19 infections (see CSP Section 5.2).

Moreover, with the outbreak of COVID-19, there is the potential for increased use of chloroquine and hydroxychloroquine to treat severely symptomatic patients, or even for prophylactic use. Chloroquine and hydroxychloroquine have shown in vitro to substantially affect the pH of the lysosome, a key intracellular compartment involved in the trafficking and payload release of T-DXd. As it is unknown whether chloroquine/hydroxychloroquine may affect the safety and efficacy of T-DXd, to be eligible for this clinical trial, use of chloroquine and hydroxychloroquine treatment must be completed at least 14 days prior to the first dose of T-DXd (see CSP Section 5.1). During study intervention, chloroquine and hydroxychloroquine are considered prohibited concomitant medications. However, in case treatment with chloroquine or hydroxychloroquine treatment is absolutely required for COVID-19, study intervention must be interrupted. After chloroquine or hydroxychloroquine is administered for COVID-19, then a washout period of at least 14 days is required before restarting study intervention.

Lastly, due to the potential overlapping impact of T-DXd and COVID-19 on the lung, the sponsor has also provided in this appendix, a dose modification and management plan for participants with confirmed or suspected COVID-19 who are being treated with T-DXd.

With these measures in place, it is considered the anticipated potential benefits for the participants enrolled in this study outweigh the potential risks.

Inclusion Criteria

- Has adequate treatment washout period prior to initiation of study intervention, defined as:
  - Chloroquine/hydroxychloroquine: ≥ 14 days

Prior and Concomitant Medications

Concomitant treatment with chloroquine or hydroxychloroquine is not allowed during the study intervention. If treatment with chloroquine or hydroxychloroquine is absolutely required for COVID-19, study intervention must be interrupted. If chloroquine or hydroxychloroquine is administered, then a washout period of at least 14 days is required before restarting study intervention.

COVID-19 Assessment(s)

All confirmed or suspected COVID-19 infection events must be recorded in the eCRF.

Serum samples will be used for COVID-19 testing from each participant who provides consent. Samples will be collected prior to the study intervention infusion, shipped to a central laboratory, and stored there until the tests become available.

Serum and PK sample collection, preparation, handling, storage, and shipping instructions are provided in the Study Laboratory Manual.

Dose modification criteria

All confirmed or suspected COVID-19 infection events must be recorded in the eCRF. Dose modifications will be based on the worst CTCAE grade. All interruptions or modifications must be recorded on the AE and drug administration eCRFs. Please use CTCAE v5.0 general grading criteria to evaluate COVID-19. All dose modifications (discontinuation, interruptions or reductions) must be recorded on the AE and drug administration eCRFs.

Dose modification criteria for suspected or confirmed COVID-19

If COVID-19 infection is suspected, delay T-DXd and rule out COVID-19 per local guidance.

- If COVID-19 is ruled out, follow dose modification and management guidelines as outlined in the CSP.
- If COVID-19 is confirmed or diagnosis is suspected after evaluation, follow dose modification as outlined below and manage COVID-19 per local guidance until recovery of COVID-19. Recovery is defined as no signs/symptoms of COVID-19, at least 1 negative RT-PCR test result[5], and nearly or completely resolved chest CT findings. Then follow dose modifications in Table 22.

Table 22 COVID-19 Dose Modification Criteria

COVID-19 Worst Toxicity NCI-CTCAE Version 5.0 Grade (unless otherwise specified)	Schedule Modification for T-DXd
Grade 1	Resume T-DXd at the same dose [a]
Grade 2	Resume T-DXd at the same dose if chest CT findings are completely resolved [a]<br>Reduce by 1 dose level if chest CT findings are nearly resolved

[5] If PCR testing is not available, the participant must not have any sign/symptoms for at least 2 weeks, in addition to meeting the requirement for chest CT imaging.

COVID-19 Worst Toxicity NCI-CTCAE Version 5.0 Grade (unless otherwise specified)	Schedule Modification for T-DXd
Grade 3	Reduce by 1 dose level if chest CT findings are completely resolved<br>Discontinue T-DXd if chest CT findings are **not** completely resolved
Grade 4	Discontinue T-DXd

[a] Closely monitor signs/symptoms after resuming study intervention, initially with a phone call every 3 days for the first week, and then with a weekly phone call thereafter, for a total of 6 weeks.
CT = computed tomography; NCI-CTCAE = National Cancer Institute-Common Terminology Criteria for Adverse Events; COVID-19 = coronavirus 2019-nCoV; T-DX-d = trastuzumab deruxtecan.

- In addition to the recommendations outlined in Table 22, investigators may consider dose modifications of T-DXd according to the participant's condition and after discussion with the study physician or designee.
- If an event is suspected to be drug-related ILD/pneumonitis, manage per protocol ILD/pneumonitis management guideline.

Appendix I Changes Related to Mitigation of Study Disruptions Due to Cases of Civil Crisis, Natural Disaster, or Public Health Crisis

Note: Changes below should be implemented only during study disruptions due to any of or a combination of civil crisis, natural disaster, or public health crisis (eg, during quarantines and resulting site closures, regional travel restrictions and considerations if site personnel or study participants become infected with COVID-19 or similar pandemic infection) during which participants may not wish to or may be unable to visit the study site for study visits. These changes should only be implemented if allowable by local/regional guidelines and following agreement from the sponsor.

Refer to the Study Instruction Manual for Mitigation Due to Civil Crisis, Natural Disaster, or Public Health Crisis for step-by-step guidance.

Reconsent of Study Participants During Study Interruptions

During study interruptions, it may not be possible for the participants to complete study visits and assessments on site and alternative means for carrying out the visits and assessments may be necessary, eg, remote visits. Reconsent should be obtained for the alternative means of carrying out visits and assessments and should be obtained prior to performing the procedures described in Section 1.3. Local and regional regulations and/or guidelines regarding reconsent of study participants should be checked and followed. Reconsent may be verbal if allowed by local and regional guidelines (note, in the case of verbal reconsent the ICF should be signed at the participant's next contact with the study site). Visiting the study sites for the sole purpose of obtaining reconsent should be avoided.

Rescreening of Participants to Reconfirm Study Eligibility

Additional rescreening for screen failure due to study disruption can be performed in previously screened participants. The investigator should confirm this with the designated study physician.

In addition, during study disruption there may be a delay between confirming eligibility of a participant and either enrolment into the study or commencing of dosing with study intervention. If this delay is outside the screening window specified in Section 1.3, the participant will need to be rescreened to reconfirm eligibility before commencing study procedures. This will provide another opportunity to rescreen a participant in addition to that detailed in Section 5.4. The procedures detailed in Section 1.3.1 must be undertaken to confirm eligibility. Rescreened participants should retain the same participant number as for the initial screening.

Appendix J Guidance for Management of Participants with Drug-induced ILD/Pneumonitis

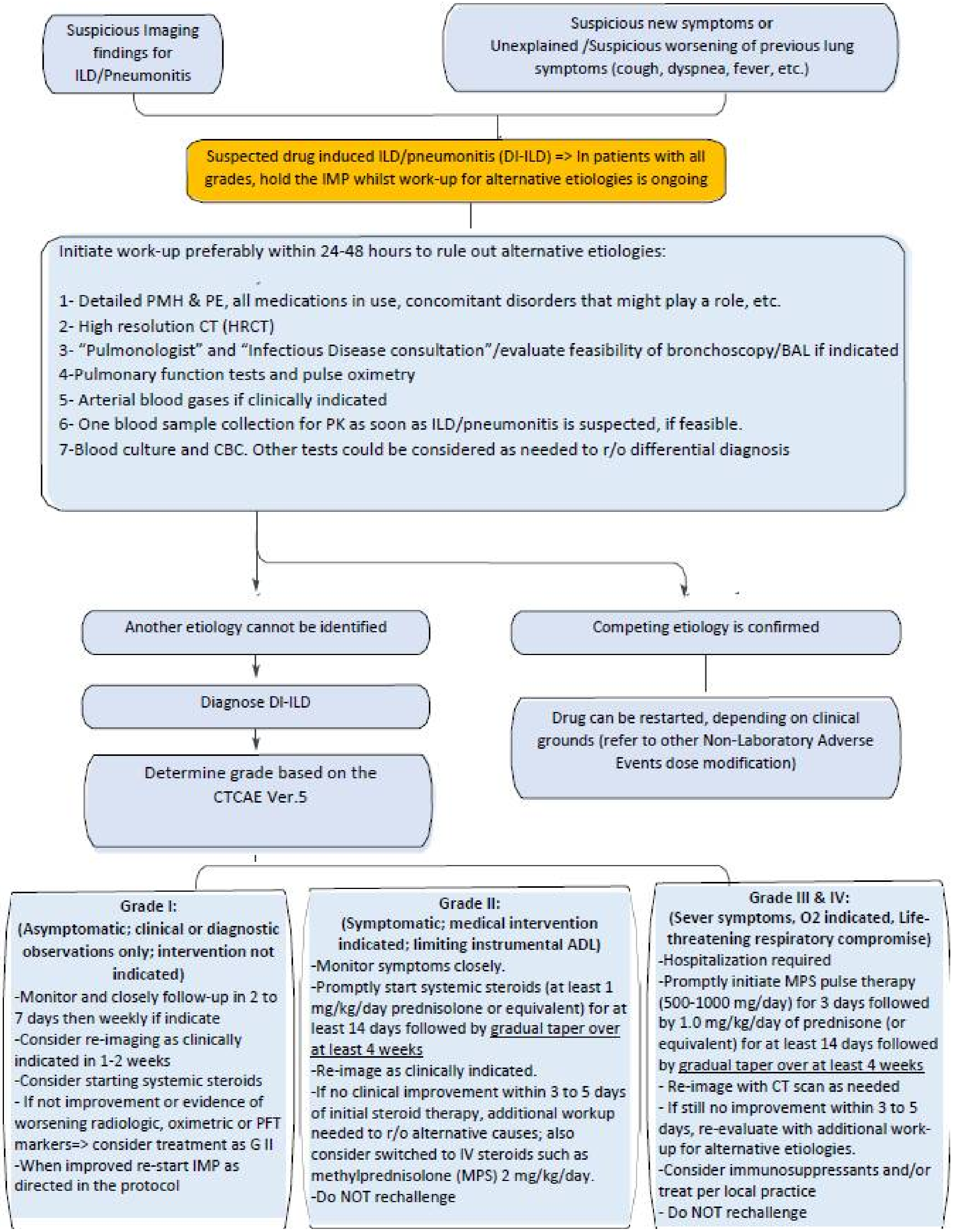

Appendix K Toxicity Management Guidelines

Table 23 Toxicity Management Guidelines for T-DXd

Worst toxicity CTCAE v 5.0 Grade (unless otherwise specified)	Management guidelines for T-DXd
[redacted]	[redacted]
**Infusion-related reaction**	
[redacted]	[redacted]
[redacted]	[redacted]
[redacted]	[redacted]
[redacted]	
[redacted] **decreased**	
[redacted]	[redacted]
[redacted]	[redacted]
[redacted]	[redacted]
[redacted]	
[redacted]	[redacted]

Table 23 Toxicity Management Guidelines for T-DXd

Worst toxicity CTCAE v 5.0 Grade (unless otherwise specified)	Management guidelines for T-DXd
[redacted]	[redacted]
[redacted]	
[redacted]	[redacted]
[redacted]	[redacted]
[redacted]	
[redacted]	[redacted]
[redacted]	[redacted]
[redacted]	
[redacted]	[redacted]
[redacted]	[redacted]
[redacted]	[redacted]
[redacted]	[redacted]
[redacted]	[redacted]
[redacted]	

Table 23 Toxicity Management Guidelines for T-DXd

Worst toxicity CTCAE v 5.0 Grade (unless otherwise specified)	Management guidelines for T-DXd
[redacted]	[redacted]
[redacted]	[redacted]
[redacted]	[redacted]

Table 23 Toxicity Management Guidelines for T-DXd

Worst toxicity CTCAE v 5.0 Grade (unless otherwise specified)	Management guidelines for T-DXd
[redacted]	[redacted]
[redacted]	[redacted]

Table 23 Toxicity Management Guidelines for T-DXd

**Worst toxicity CTCAE v 5.0 Grade (unless otherwise specified)**	**Management guidelines for T-DXd**
[redacted]	[redacted]
[redacted]	
[redacted]	[redacted]
[redacted]	[redacted]
[redacted]	
[redacted]	[redacted]
[redacted]	[redacted]
[redacted]	
[redacted]	
[redacted]	[redacted]
[redacted]	
[redacted]	[redacted]

Table 23 Toxicity Management Guidelines for T-DXd

Worst toxicity CTCAE v 5.0 Grade (unless otherwise specified)	Management guidelines for T-DXd
[redacted]	[redacted]
[redacted]	[redacted]
[redacted]	[redacted]
[redacted]	
[redacted]	[redacted]
[redacted]	[redacted]
[redacted]	[redacted]

Table 23 Toxicity Management Guidelines for T-DXd

Worst toxicity CTCAE v 5.0 Grade (unless otherwise specified)	Management guidelines for T-DXd
[redacted]	
[redacted]<br>[redacted]	[redacted]
[redacted]	
[redacted]<br>[redacted]	[redacted]<br>[redacted]<br>[redacted]<br>[redacted]<br>[redacted]
[redacted]	
[redacted]	
[redacted]	[redacted]<br>[redacted]
[redacted]	
[redacted]	[redacted]<br>[redacted]
[redacted]	[redacted]
[redacted]	

Table 23 Toxicity Management Guidelines for T-DXd

[redacted]	[redacted]
[redacted]	[redacted]
[redacted]	[redacted]
[redacted]	
[redacted]	[redacted]
[redacted]	[redacted]

[redacted]

Appendix L Abbreviations

Abbreviation or Special Term	Explanation
ADA(s)	anti-drug antibody(ies)
ADC	antibody-drug conjugate
AE	adverse event
AESI	adverse event of special interest
ALT	alanine aminotransferase
aPTT	activated partial thromboplastin time
AST	aspartate aminotransferase
BOR	best objective response
CART	cell-free and concentrated ascites reinfusion therapy
CHF	congestive heart failure
CI	confidence interval
COPD	chronic obstructive pulmonary disorder
COVID-19	coronavirus 2019-nCoV, also referred to as severe acute respiratory syndrome coronavirus 2
CR	complete response
CrCL	calculated creatinine clearance
CSP	Clinical Study Protocol
CSR	Clinical Study Report
CT	computed tomography
CTCAE	Common Terminology Criteria for Adverse Events
DAR	drug-antibody ratio
DCO	data cutoff
DCR	disease control rate
DILI	drug-induced liver injury
DLCO	diffusion capacity of the lungs for carbon monoxide
DNA	deoxyribonucleic acid
DoR	duration of response
DXd	MAAA-1181a (deruxtecan)
ECG	electrocardiogram
ECHO	echocardiogram
ECOG	Eastern Cooperative Oncology Group
eCRF	electronic Case Report Form
ED	early discontinuation
EDC	electronic data capture

Abbreviation or Special Term	Explanation
EOT	end of treatment
ER	exposure-response
FAS	Full Analysis Set
FDA	Food and Drug Administration
FEV	forced expiratory volume
FFPE	formalin-fixed and paraffin-embedded
GC	gastric cancer
GCP	Good Clinical Practice
GEJ	gastroesophageal junction
GI	gastrointestinal
HBV	Hepatitis B virus
HCV	hepatitis C virus
HER2	human epidermal growth factor receptor 2
HIV	human immunodeficiency virus
HL	Hy's Law
HR	hazard ratio
HRCT	high-resolution computed tomography
HRT	hormone replacement therapy
5-HT3	5-hydroxytryptamine 3
IATA	International Airline Transportation Association
IB	Investigator's Brochure
ICF	informed consent form
ICH	International Council for Harmonisation
ICR	Independent Central Review
iCRO	imaging Contract Research Organisation
IEC	Independent Ethics Committee
IHC	immunochemistry
ILD	interstitial lung disease
INR	international normalised ratio
IRB	Institutional Review Board
IRR	infusion-related reactions
IRT	interactive response technology
ISH	in situ hybridisation
ITT	Intent to treat
IV	intravenous

Abbreviation or Special Term	Explanation
LVEF	left ventricular ejection fraction
MAAA-1162a	drug-linker, the complex of MAAA-1181a and a maleimide tetrapeptide linker
MAAA-1181a	the drug released from T-DXd, a derivative of exatecan, a topoisomerase I inhibitor
MAAL-9001	the intermediate of T-DXd, a recombinant humanised anti-HER2 IgG1 monoclonal antibody with the same amino acid sequence as trastuzumab
mAb	monoclonal antibody
MedDRA	Medical Dictionary for Regulatory Activities
MI	myocardial infarction
MRI	magnetic resonance imaging
MUGA	multigated acquisition
NCCN	National Comprehensive Cancer Network
NCI	National Cancer Institute
NE	not evaluable
NK1	neurokinin 1
NL	new lesion
NMPA	National Medical Products Administration
NTL	non-target lesion
NYHA	New York Heart Association
ORR	objective response rate
OS	overall survival
PD	progression of disease/progressive disease
PET	positron emission tomography
PFS	progression-free survival
PHL	Potential Hy's Law
PK	pharmacokinetic
popPK	population PK
PR	partial response
PT	prothrombin time
PTT	partial thromboplastin time
QTc	corrected QT interval
QTcF	QT interval corrected by Fridericia's formula
RECIST 1.1	Response Evaluation Criteria in Solid Tumours, Version 1.1
RES	response evaluable set
RNA	ribonucleic acid
SAE	serious adverse event

Abbreviation or Special Term	Explanation
SAP	Statistical Analysis Plan
SD	stable disease
SFU	safety follow-up
SoA	Schedule of Activities
SoC	standard of care
SpO_2	peripheral capillary oxygen saturation
TBL	total bilirubin
T-DM1	trastuzumab emtansine
TEAE	treatment emergent adverse event
TMG	toxicity management guideline
T-DXd	trastuzumab deruxtecan
TL	target lesion
ULN	upper limit of normal
WOCBP	women of childbearing potential
w/v	weight per volume

11 REFERENCES

Bang et al 2010
Bang TJ, Van Cutsem E, Feyereislova A, et al. Trastuzumab in combination with chemotherapy versus chemotherapy alone for treatment of HER2-positive advanced gastric or gastroesopheal junction cancer (ToGA): a phase 3, open-label, randomised controlled trial. Lancet. 2010;376:687-697.

Brookmeyer and Crowley 1982
Brookmeyer R, Crowley J. A Confidence Interval for the Median Survival Time. Biometrics. 1982;38:29–41.

Chen et al 2020
Chen L-T, Satoh T, Ryu M-H, Chao Y, Kato K, Chung HC, et al. A phase 3 study of nivolumab in previously treated advanced gastric or gastroesophageal junction cancer (ATTRACTION 2): 2 year update data. Gastric Cancer. 2020;23(3):510-19.

Chen et al 2016
Chen W, Zheng R, Baade PD, Zhang S, Zeng H, Bray F, et al. Cancer statistics in China, 2015. CA Cancer J Clin. 2016;66:115–32.

Cho et al 2013
Cho EY, Park K, Do I, Cho J, Kim J, Lee J, et al. Heterogeneity of ERBB2 in gastric carcinomas: a study of tissue microarray and matched primary and metastatic carcinomas. Mod Pathol. 2013;26(5):677-84.

Chua and Merrett 2012
Chua TC, Merrett ND. Clinicopathologic factors associated with HER2-positive gastric cancer and its impact on survival outcomes-A systematic review. Int J Cancer. 2012;130:2845-56.

Cutsem et al 2021
Cutsem EV, Bartolomeo MD, Smyth E, et al. Primary analysis of a phase 2 single arm trial of Trastuzumab Deruxtecan (T-DXd) in western patients with HER2 positive (HER2+) unresectable or metastatic gastric or gastroesophageal junction (GEJ) cancer who progressed on or after a Trastuzumab containing regimen. ESMO 2021, LBA 55.

Fan et al 2013
Fan XS, Chen JY, Li CF, et al. Differences in HER2 over-expression between proximal and distal gastric cancers in the Chinese population. World J Gastroenterol. 2013;19(21): 3316-3323.

GLOBOCAN 2018a
GLOBOCAN 2018a. Stomach cancer: estimated incidence, mortality and prevalence worldwide in 2018. Available at: http://gco.iarc.fr/today/data/factsheets/cancers/7-Stomach-

fact-sheet.pdf. Accessed 25 Sep 2018.

GLOBOCAN 2018b
GLOBOCAN 2018b. Population Fact Sheets. Accessed 16 October 2018 from URL: http://gco.iarc.fr/today/data/factsheets/populations/160-china-fact-sheets.pdf. Accessed 25 Sep 2018.

Gomez-Martin et al 2012
Gomez-Martin C, Garralda E, Echarri MJ, Ballesteros A, Arcediano A, Rodriguez-Peralto JL, et al. HER2/neu testing for anti-HER2-based therapies in patients with unresectable and/or metastatic gastric cancer. J Clin Pathol. 2012;65:751-7.

Grabsch et al 2010
Grabsch H, Sivakumar S, Gray S, Gabbert HE, Muller W. HER2 expression in gastric cancer: Rare, heterogeneous and of no prognostic value - conclusions from 924 cases of two independent series. Analyt Cell Pathol. 2010;32:57-65.

Gravalos and Jimeno 2008
Gravalos C, Jimeno A. HER2 in gastric cancer: a new prognostic factor and a novel therapeutic target. Ann Oncol. 2008;19:1523-9.

Hofmann et al 2008
Hofmann M, Stoss O, Shi D, et al. Assessment of a HER2 scoring system for gastric cancer: results from a validation study. Histopathology. 2008;52:797-805.

Huang et al 2013
Huang D, Lu N, Fan Q, Sheng W, Bu H, Jin X, et al. HER2 Status in Gastric and Gastroesophageal Junction Cancer Assessed by Local and Central Laboratories: Chinese Results of the HER-EAGLE Study. PLoS ONE. 2013;8(11):e80290.

Huang et al 2018
Huang D, Li ZS, Fan XS, et al. HER2 status in gastric adenocarcinoma of Chinese: a multicenter study of 40842 patients. Chin J Pathol, 2018, 47: 822-826.

Janjigian et al 2012
Janjigian YY, Werner D, Pauligk C, Steinmetz K, Kelsen DP, Jäger E, et al. Prognosis of metastatic gastric and gastroesophageal junction cancer by HER2 status: a European and USA International collaborative analysis. Ann Oncol. 2012;23(10):2656-62.

Jorgensen and Hersom 2012
Jorgensen JT, Hersom M. HER2 as a prognostic marker in gastric cancer - a systematic analysis of data from the literature. J Cancer. 2012;3:137-44.

Kato et al 2018
Kato S, Okamura R, Baumgartner JM, Patel H, Leichman L, Kelly K, et al. Analysis of circulating tumor DNA and clinical correlates in patients with esophageal, gastroesophageal junction, and gastric adenocarcinoma. Clin Cancer Res. 2018;24:6248-56.

Kim et al 2011
Kim KC, Koh YW, Chang H-M, Kim TH, Yook JH, Kim BS, et al. Evaluation of HER2 protein expression in gastric carcinomas: comparative analysis of 1,414 cases of whole-tissue sections and 595 cases of tissue microarrays. Ann Surg Oncol. 2011;18(10):2833-40.

Klein et al 1997
Klein JP, Moeschberger ML. Survival Analysis: Techniques for Censored and Truncated Data. 1997. New York: Springer-Verlag.

Kunz et al 2012
Kunz PL, Mojtahed A, Fisher GA, Ford JM, Chang DT, Balise RR, et al. HER2 expression in gastric and gastroesophageal junction adenocarcinoma in a US population: clinicopathologic analysis with proposed approach to HER2 assessment. Appl lmmunohistochem Mol Morphol. 2012;20:13-24.

Lee et al 2013
Lee HE, Park KU, Yoo SB, Nam SK, Park DJ, Kim H-H, et al. Clinical significance of intratumoral HER2 heterogeneity in gastric cancer. Eur J Cancer. 2013;49(6):1448-57.

Li et al 2016
Li J, Qin S, Xu J, Xiong J, Wu C, Bai Y, et al. Randomized, double-blind, placebo-controlled phase III trial of apatinib in patients with chemotherapy-refractory advanced or metastatic adenocarcinoma of the stomach or gastroesophageal junction. J Clin Oncol. 2016;34(13):1448–54.

Li et al 2018
Li J, Ying C, Jun G, He J. Chinese Society of Clinical Oncology: clinical guidelines for the diagnosis and treatment of gastric cancer (2018.V1). People's Medical Publishing House.

Meza-Junco and Sawyer 2012
Meza-Junco J, Sawyer MB. Metastatic gastric cancer - focus on targeted therapies. Biologics. 2012:6137-46.

Minashi and Hironaka 2015
Minashi K, Hironaka S. Advances in secondary chemotherapy for gastric cancer. Gan To Kagaku Ryoho. 2015;42(4):398-402.

NCCN 2021
NCCN gastric cancer guideline Version 5. 2021: https://www.nccn.org/guidelines/guidelines-

detail?category=1&id=1434

NCI 2019
PDQ® Adult Treatment Editorial Board. PDQ Gastric Cancer Treatment. Bethesda, MD: National Cancer Institute. Updated 05/07/2019. Available at: https://www.cancer.gov/types/stomach/hp/stomach-treatment-pdq. Accessed 10/28/2020. [PMID: 26389209].

Ogitani et al 2016
Ogitani Y, Aida T, Hagihara K, Yamaguchi J, Ishii C, Harada N, et al. DS-8201a, a novel HER2-targeting ADC with a novel DNA topoisomerase I inhibitor, demonstrates a promising antitumor efficacy with differentiation form T-DM1. Clin Cancer Res. 2016;22(20):5097-108.

Park et al 2006
Park DI, Yun JW, Park JH, Oh SJ, Kim HJ, Cho YK, et al. HER-2/neu amplification is an independent prognostic factor in gastric cancer. Dig Dis Sci. 2006;51(8):1371-9.

Peng et al 2021
Peng Z, Liu TS, Wei J, et al. Efficacy and safety of a novel anti-HER2 therapeutic antibody RC48 in patients with HER2-overexpressing, locally advanced or metastatic gastric or astroesophageal junction cancer: a single-arm phase II study. Cancer Communications. 2021;1-10.

Pietrantonio et al 2016
Pietrantonio F, Caporale M, Morano F, Scartozzi M, Gloghini A, De Vita F, et al. HER2 loss in HER2-positive gastric or gastroesophageal cancer after trastuzumab therapy: Implication for further clinical research. Int J Cancer. 2016;139(12):2859-64.

Ruschoff et al 2010
Roschoff J, Dietel M, Baretton G, et al. HER2 diagnostics in gastric cancer-guideline validation and development of standardized immunohistochemical testing. Virchows Arch. 2010;457:299-307.

Ruschoff et al 2012
Rüschoff J, Hanna W, Bilous M, Hofmann M, Osamura RY, Penault-Llorca F, et al. HER2 testing in gastric cancer: a practical approach. Mod Pathol. 2012;25(5):637-50.

Saeki et al 2018
Saeki H, Oki E, Kashiwada T, Arigami T, Makiyama A, Iwatsuki M, et al. Re-evaluation of HER2 status in patients with HER2-positive advanced or recurrent gastric cancer refractory to trastuzumab (KSCC1604). Eur J Cancer. 2018;105:41-9.

Shan et al 2013
Shan L, Ying J, Lu N. HER2 expression and relevant clinicopathological features in gastric

and gastroesophageal junction adenocarcinoma in a Chinese population. Diagn Pathol. 2013;8:76-82.

Sheng et al 2013
Sheng WQ, Huang D, Ying JM, Lu N, Wu HM, Liu YH, et al. HER2 status in gastric cancers: a retrospective analysis from four Chinese representative clinical centers and assessment of its prognostic significance. Ann Oncol. 2013;24:2360-4.

Shitara et al 2020
Shitara K, Bang YJ, Iwasa S, et al. Trastuzumab deruxtecan in previously treated HER2-positve gastric cancer. N Engl J Med. 2020; 382 (25): 2419-2430.

Thuss-Patience et al 2017
Thuss-Patience PC, Shah MA, Ohtsu A, Van Cutsem E, Ajani JA, Castro H, et al. Trastuzumab emtansine versus taxane use for previously treated HER2-positive locally advanced or metastatic gastric or gastro-oesophageal junction adenocarcinoma (GATSBY): an international randomised, open-label, adaptive, phase 2/3 study. Lancet Oncol. 2017;18(5):640-53.

Torre et al 2012
Torre LA, Bray F, Siegel RL, Ferlay J, Lortet-Tieulent J, Jenal A. Global cancer statistics, 2012. CA Cancer J Clin. 2015;65:87-108.

Treatment Guidelines 2019
Guidelines for the Diagnosis and Treatment of Gastric Cancer (2018 edition).
J Multidisciplinary Cancer Management. 2019;5(1):55-82.

Xuan 2014
Xuan L. Retrospective clinical study of third-line chemotherapy for advanced gastric cancer[D]. Nan Chang: The First Affiliated Hospital of Nanchang University. 2014:1-38.

Yang et al 2018
Yang L, Zheng RS, Wang N, Yuan Y, Liu S, Li H, et al. Incidence and mortality of stomach cancer in China, 2014. Chin J Cancer Res. 2018;30(3):291-8.

Yano et al 2006
Yano T, Doi T, Ohtsu A, Boku N, Hashizume K, Nakanishi M, et al. Comparison of HER2 gene amplication assessed by fluorescence in situ hybridization and HER2 protein expression assessed by immunohistochemistry in gastric cancer. Oncol Rep. 2006;15(1):65-71.

Yoon et al 2018
Yoon HH, Shi Q, Sukov WR, Wiktor AE, Khan M, Sattler CA, et al. Association of HER2/ErbB2 expression and gene amplification with pathological features and prognosis in esophageal adenocarcinomas. Clin Cancer Res. 2012;18(2):546-54.

SIGNATURE PAGE

This is a representation of an electronic record that was signed electronically and this page is the manifestation of the electronic signature

**Document Name:** d9676c00002-csp-v2		
**Document Title:**	D9676C00002 Clinical Study Protocol version 2	
**Document ID:**	[redacted]	
**Version Label:**	[redacted]	
**Server Date (dd-MMM-yyyy HH:mm 'UTC'Z)**	**Signed by**	**Meaning of Signature**
[redacted]	[redacted]	[redacted]
[redacted]	[redacted]	[redacted]
[redacted]	[redacted]	[redacted]
[redacted]	[redacted]	[redacted]
[redacted]	[redacted]	[redacted]

Notes: (1) Document details as stored in ANGEL, an AstraZeneca document management system.
