# Supplementary material for: Trastuzumab deruxtecan in patients from China with previously treated human epidermal growth factor receptor 2–positive locally advanced/metastatic gastric or gastroesophageal junction adenocarcinoma (DESTINY-Gastric06): results from a single-arm, multicenter, phase 2 trial
Source: eClinicalMedicine. 2025 Aug 11;87:103404. doi: 10.1016/j.eclinm.2025.103404 (PMC12359162; doi:10.1016/j.eclinm.2025.103404)
Supplement: Supplementary Appendix [file mmc2.docx]

# Supplementary appendix

**Trastuzumab deruxtecan in patients from China with previously treated human epidermal growth factor receptor 2–positive locally advanced / metastatic gastric or gastroesophageal junction adenocarcinoma (DESTINY-Gastric06 trial): results from a single-arm, multicenter, phase 2 trial**

Zhi Peng; Ping Chen; Jin Lu; Yiye Wan; Yulong Zheng; Feng Ye; Jianwei Yang; Ying Liu; Hongming Pan; Meili Sun; Qingxia Fan; Ying Yuan; Kai Chen; Zhuoer Sun; Han Tian; Ye Xia; Lin Shen

**Table of contents**

List of center numbers, and names and addresses of the ethics committees for the DESTINY-Gastric06 trial 2

**List of center numbers, and names and addresses of the ethics committees for the**

**DESTINY-Gastric06 trial**

| Center number | Name and address of independent ethics committee |
| --- | --- |
| 1301 | EC of Beijing Cancer Hospital,  52 Fucheng Road, Haidian District, Beijing |
| 1302 | EC of Peking University 3rd Hospital,  49 North Garden Road, Haidian District, Beijing |
| 1303 | EC of Sichuan Cancer Hospital,  55 Renmin South Road, Section 4, Wuhou District, Chengdu |
| 1304 | EC of The Second Affiliated Hospital Zhejiang University School of Medicine,  88 Jiefang Road, Shangcheng District, Hangzhou |
| 1305 | EC of Lanzhou University Second Hospital,  82 Cuiyingmen, Chengguan District, Lanzhou |
| 1306 | IEC of General Hospital of Ningxia Medical University,  804 Shengli South Street, Xingqing District, Yinchuan |
| 1307 | EC of Anhui Provincial Cancer Hospital,  107 East Huanhu Road, Shushan District, Hefei |
| 1309 | EC of Hubei Cancer Hospital,  116 Zhuodaoquan South Road, Hongshan District, Wuhan |
| 1310 | EC Anhui Provincial Hospital,  17 Lujiang Road, Luyang District, Hefei |
| 1312 | IEC of The First Affiliated Hospital of Sun Yat-Sen University,  5 Zhusigang 2nd Road, Yuexiu District, Guangzhou |
| 1314 | IEC of Jiangxi Cancer Hospital,  519 East Beijing Road, Qingshanhu District, Nanchang |
| 1315 | EC of Affiliated Hospital of North Sichuan Medical College,  1 Maoyuannan Road, Shunqing District, Nanchong |
| 1316 | EC of The First Affiliated Hospital of Suzhou University,  188 Shizi Road, Gusu District, Suzhou |
| 1317 | IEC of Sir Run Run Shaw Hosp School of Medicine, Zhejia University,  3 East Qingchun Road, Jianggan District, Hangzhou |
| 1318 | EC of First Affiliated Hospital of China Medical University,  155 Nanjing Bei Jie, Heping District, Shenyang |
| 1319 | EC of The First Affiliated Hospital of Zhengzhou University,  1 Jianshe Donglu, Erqi District, Zhengzhou |
| 1320 | IEC of Henan Cancer Hospital,  127 Dongming Road, Jinshui District, Zhengzhou |
| 1322 | IEC of Jinan Central Hospital,  105 Jiefang Road, Lixia District, Jinan |
| 1323 | EC of Xiamen First Hospital,  55 Zhenhai Road, Siming District, Xiamen |
| 1324 | EC Zhongshan Hospital Fudan University,  180 Fenlin Road, Xuhui District, Shanghai |
| 1325 | EC of The First Affiliated Hospital of Zhejiang University,  79 Qingchun Road, Shangcheng District, Hangzhou |
| 1326 | EC Xiangya Hospital of Center-South University,  87 Xiangya Road, Kaifu District, Changsha |
| 1327 | [EC of Fujian Province Cancer Hospital](https://astrazeneca-vcv.veevavault.com/ui/#object/organization__v/0OR000000005H94),  420 Fuma Road, Jinan District, Fuzhou |
| 1328 | [EC of Jiangsu Cancer Hospital](https://astrazeneca-vcv.veevavault.com/ui/#object/organization__v/0OR000000005I28),  42 Baiziting, Xuanwu District, Nanjing |

All independent ethics committees were located in China.
EC, ethics committee; IEC, independent ethics committee
